# Modular, automated synthesis of spirocyclic tetrahydronaphthyridines from primary alkylamines

**DOI:** 10.1038/s42004-023-01012-2

**Published:** 2023-10-04

**Authors:** Qiao Cao, Joshua D. Tibbetts, Gail L. Wrigley, Adam P. Smalley, Alexander J. Cresswell

**Affiliations:** 1 Department of Chemistry, University of Bath, Claverton Down, Bath, BA2 7AY UK; 2 Medicinal Chemistry, Oncology R&D, AstraZeneca, Cambridge, CB4 0WG UK; 3 UCB, 216 Bath Road, Slough, SL1 3WE UK

**Keywords:** Synthetic chemistry methodology, Combinatorial libraries, Automation, Flow chemistry, Photocatalysis

## Abstract

Spirocyclic tetrahydronaphthyridines (THNs) are valuable scaffolds for drug discovery campaigns, but access to this 3D chemical space is hampered by a lack of modular and scalable synthetic methods. We hereby report an automated, continuous flow synthesis of α-alkylated and spirocyclic 1,2,3,4-tetrahydro-1,8-naphthyridines (“1,8-THNs”), in addition to their regioisomeric 1,6-THN analogues, from abundant primary amine feedstocks. An annulative disconnection approach based on photoredox-catalysed hydroaminoalkylation (HAA) of halogenated vinylpyridines is sequenced in combination with intramolecular S_N_Ar *N*-arylation. To access the remaining 1,7- and 1,5-THN isomers, a photoredox-catalysed HAA step is telescoped with a palladium-catalysed C–N bond formation. Altogether, this provides a highly modular access to four isomeric THN cores from a common set of unprotected primary amine starting materials, using the same bond disconnections. The simplifying power of the methodology is illustrated by a concise synthesis of the spirocyclic THN core of Pfizer’s MC4R antagonist PF-07258669.

## Abstract (web summary)

Spirocyclic tetrahydronaphthyridines (THNs) are valuable structural motifs in medicinal chemistry, but the modular and scalable synthesis of this specific motif remains challenging. Here, the authors develop an automated and continuous flow synthesis of 1,8-THN and 1,6-THN analogues based on photoredox-catalysed hydroaminoalkylation, demonstrating the concise synthesis of the spirocyclic THN core of Pfizer’s MC4R antagonist PF-07258669.

## Introduction

**Fig. 1:**
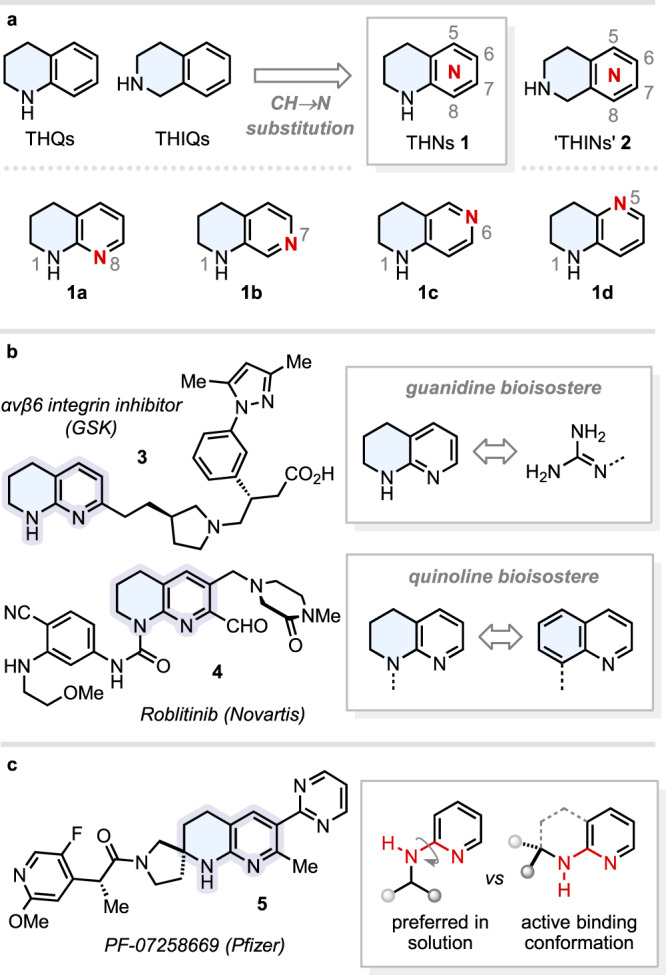
Tetrahydronaphthyridines and their importance. **a** Tetrahydronaphthyridine (THN) isomers. **b** THNs **1** in drug development. **c** Spirocyclic THNs **1** in drug development.

Bicyclic compounds featuring saturated *N*-heterocycles fused to (hetero)aromatic units are highly prized in medicinal chemistry^1–4^, offering a combination of polar functionality, high Fsp^3^-content, and rigidly disposed groups on the aromatic core that can engage in key interactions with a protein target (e.g., H-bonds)^5,6^. Tetrahydronaphthyridines (THNs) are semi-saturated bicycles that ring-fuse a piperidine with a pyridine—these in turn being the two most popular *N*-heterocycles deployed in small-molecule pharmaceuticals^7,8^. Positioning of the two THN nitrogen atoms generates eight different structural isomers: four of which (**1a**–**d**) can be considered as CH → N bioisosteres of tetrahydroquinolines (THQs), and the remaining four (structures **2**) as CH → N bioisosteres of tetrahydroisoquinolines (THIQs) (Fig. 1a). The substitution of CH units for N atoms in (hetero)aromatic systems can impart orders of magnitude improvements in key physicochemical (e.g., solubility) and pharmacological parameters^9^, and synthetic strategies that could provide facile access to any THN isomer (e.g., **1a**–**d**) would be highly enabling. Without a trivial naming convention for THNs, we shall hereafter refer to structures **1** as “THNs” and their isomeric counterparts **2** as “THINs”^10–16^, by analogy to THQs and THIQs. Amongst other applications^17,18^, THNs have found use as guanidine mimetics of the arginine binding motif in RGD-binding integrin inhibitors (e.g., **3**)^19,20^. Scaffold morphing of quinolines to THNs can also be an effective tactic to improve aqueous solubility, as exemplified during the development of the FGFR4 selective inhibitor Roblitinib (FGF401) **4** (Fig. 1b)^21^. Spirocyclisation of fused, semi-saturated *N*-heterocycles is also emerging as a powerful design strategy for medicinal chemistry. When compared to their flat, all-aromatic counterparts, partial saturation and installation of a spirocycle simultaneously increases Fsp^3^, reduces structural flexibility, and introduces alternative exit vectors for access to novel 3D chemical space^22^. In favourable cases, this can lead to greatly enhanced potency, selectivity, solubility, and metabolic stability^23,24^. For instance, Pfizer have exploited a spirocyclic THN as the core of their MC4R antagonist PF-07258669 **5**, which is currently in phase I clinical trials for the treatment of appetite loss (Fig. 1c)^25^. The spirocycle in **5** was rationally designed to enforce a *cis*-relationship between the N–H bond and the adjacent N(sp^2^) lone pair, which is the optimal geometry for target binding but opposite to the (*trans*) conformer favoured in solution for the non-constrained analogue.

**Fig. 2:**
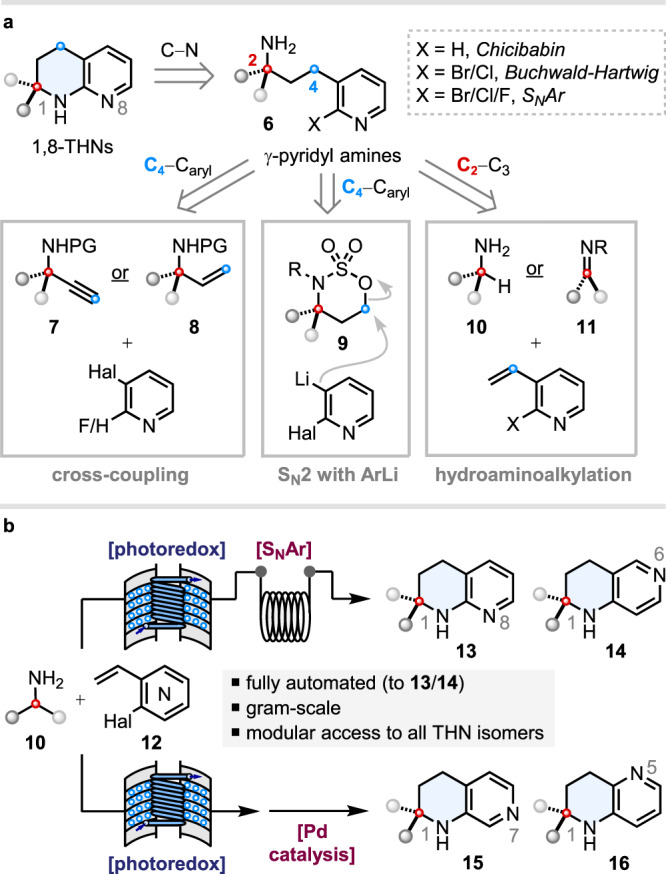
Prior art for THN synthesis and this work. **a** Selected synthetic routes to (spirocyclic) 1,8-THNs. **b** This work.

Despite the many opportunities for structural and physicochemical tuning that THNs can offer, their widespread adoption in early-stage drug discovery has likely been hampered by their poor commercial availability, and the scarcity of THN synthesis approaches that are readily amenable to library generation. Other than the semi-hydrogenation of naphthyridines^26–28^, which can present regio- and chemoselectivity challenges, several routes to THNs have been devised based on the annulation of 2-aminopyridines. These processes tend to be relatively labour-intensive^29,30^, and whilst catalytic annulations do exist^31,32^, their functional group tolerance is low. Inverse electron demand, intramolecular, hetero-Diels-Alder reactions of tethered imidazolyl^33^ or alkynyl^34–36^ triazines sequenced with N_2_ extrusion are also on record for THN synthesis, but the substrate syntheses require multiple steps. Moreover, none of the above approaches are amenable to *spirocyclic* THN synthesis. Another distinct strategy is to form THNs via the *N*-arylative cyclisation of γ-pyridyl amines, either by intramolecular Pd-catalysed C–N coupling^25,37,38^, S_N_Ar reactions^37–39^, or Chichibabin reactions^40,41^. γ-Pyridyl amines **6** can themselves be constructed via Sonogashira-hydrogenation sequences^25,41^, *B*-alkyl Suzuki-Miyaura coupling^40^, or the S_N_2 ring-opening of cyclic sulfamidates **9** with *ortho*-lithiated halopyridines^39^ (Fig. 2a). Whilst these approaches can enable access to spirocyclic THNs, the chemistry is not well suited to library synthesis, given the meagre commercial availability of α-(di)substituted propargylic amines (**7**), allylic amines (**8**), or γ-hydroxy amines as starting materials. Yu and co-workers have developed a Pd-catalysed γ-C(sp^3^)–H arylation of primary alkylamines that can access γ-pyridyl amines, and applied this to a single example of THN synthesis, but the amines amenable to this procedure are largely unfunctionalized and have limited commercial availability^42^. Recently, visible-light photoredox-catalysed approaches have been reported by ourselves and Gaunt et al., respectively, for the modular synthesis of γ-aryl primary amines by the C–C bond-forming coupling of readily available primary alkylamines **10**^37^ or ketone-derived imines **11**^38^ with styrenes. Between these two disclosures, four examples of spirocyclic 1,2,3,4-tetrahydro-1,8-naphthyridine synthesis were showcased, proceeding via the S_N_Ar cyclisation of (isolated) γ-pyridyl amines from the photoredox step.

In this work, we show that photoredox-catalysed hydroaminoalkylation^43–45^ (HAA) of halogenated vinyl pyridines, followed by intramolecular *N*-arylation via S_N_Ar, can be sequenced in continuous flow^46–56^ to enable an automated synthesis of α-alkylated and spirocyclic 1,2,3,4-tetrahydro-1,8-naphthyridines (“1,8-THNs”) **13**, in addition to their regioisomeric 1,6-THN analogues **14** (Fig. 2b). To access the corresponding 1,7- and 1,5-THN isomers—**15** and **16**, respectively—a photoredox-catalysed HAA step can be telescoped with a palladium-catalysed C–N bond formation. Altogether, this provides a highly modular approach to four THN isomers **13**–**16** from a common set of unprotected primary amine starting materials **10**, using the same bond disconnections.

## Results and discussion

### Reaction optimisation

Photoredox-catalysed hydroaminoalkylation (HAA) of 2-fluoro-3-vinylpyridine **17** with cyclohexylamine **10a** gives γ-pyridyl amine **18a** in 97% yield, and subjection of this purified material to DIPEA (1.5 equiv) in DMF at 120 °C for 20 h delivers the corresponding THN **13a** in 92% yield via intramolecular S_N_Ar cyclisation^37^. In order to sequence these reactions together in continuous flow, we transferred the chemistry to a Vapourtec R-series flow system equipped with a Uniqsis PhotoSyn LED photoreactor (420 nm LEDs, ~260 W radiant output power, 5-ml reactor coil) and a high-temperature tube reactor (up to 250 °C). As per the batch procedure, the photoredox-catalysed HAA was initially carried out with 2,4,6-tris(diphenylamino)-3,5-difluorobenzonitrile (3DPA2FBN) as the photocatalyst, and tetrabutylammonium azide (Bu_4_N^+^N_3_^–^) as the hydrogen atom transfer (HAT) catalyst^57,58^. However, we found that Bu_4_N^+^N_3_^–^ could be replaced with cheaper and far less hygroscopic sodium azide (NaN_3_), which is soluble in DMF at 0.06 M. For the S_N_Ar step, a temperature of 180 °C with *t*_R_ = 20 min proved sufficient for complete conversion (see Supplementary Table 1). By running both steps in sequence in continuous flow, an overall yield of 98% of spirocyclic THN **13a** could be obtained from 2-fluoro-3-vinylpyridine **17** and cyclohexylamine **10a** as feedstocks (in a 1:1 ratio). This corresponds to a productivity of 2.20 mmol h^–1^ (445 mg h^–1^).

### Automated continuous flow synthesis of THNs from primary alkylamines

**Fig. 3:**
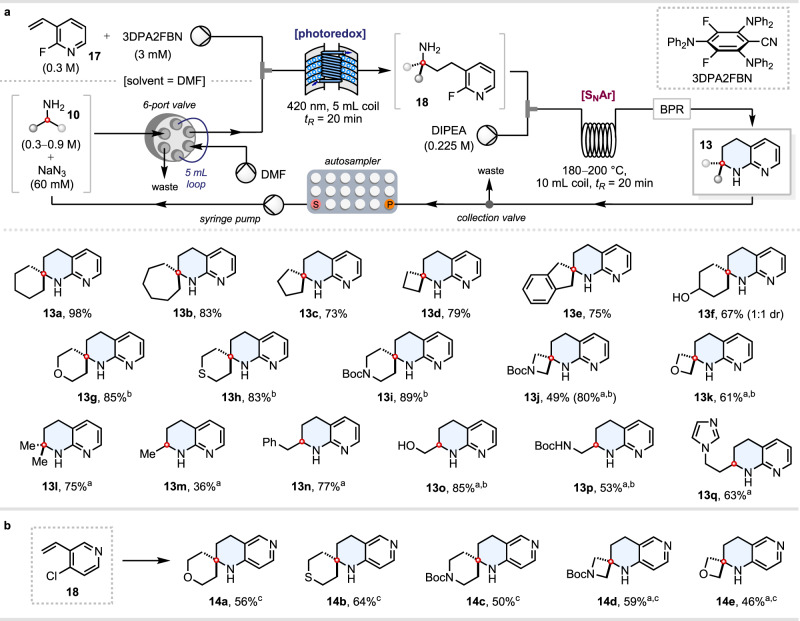
Automated continuous flow synthesis of THNs from primary alkylamines. ^a^With 3.0 equiv of amine. ^b^Second step carried out at 200 °C. ^c^Second step carried out at 220 °C. All reactions were carried out on 1.50 mmol of vinylpyridine **17** or **18**. **a** Synthesis of 1,2,3,4-tetrahydro-1,8-naphthyridines **13**. **b** Synthesis of 1,2,3,4-tetrahydro-1,6-naphthyridines **14**.

With an optimised continuous flow protocol in hand, we next sought to execute an automated library synthesis of ‘lead-like’^5,59^ THN products **13**, using an autosampler to sequentially load different amine substrates into the Vapourtec flow system. The same autosampler also serves as a fraction collector, into which the steady-state solutions of each product **13** are dispensed (Fig. 3a and see Supplementary Data 1 for NMR spectra of all compounds). Each run using 1.50 mmol of the vinylpyridine substrate takes ~90 min, which corresponds to 16 compounds in a 24 h period, or 40 compounds total over 60 h if all rack positions are utilised. Cyclic primary amines **10a**–**d** of varying ring sizes were well tolerated, and amine **10e** bearing benzylic C–H bonds also participated smoothly. Various functionalities including free hydroxyl groups (**13f**, **o**), ethers (**13g**, **k**), thioethers (**13h**), carbamates (**13i**, **j**, **p**), and imidazoles (**13q**) proved compatible with the process. For amines bearing electronegative atoms attached to the β- or γ-carbon (**10g**–**k**, **o**, **p**), a slightly elevated temperature of 200 °C proved necessary in most cases to drive the S_N_Ar step to completion within the 20 min residence time. Strained four-membered ring substrates 3-amino-*N*-Boc-azetidine **10j** and 3-aminooxetane **10k** proved especially challenging for the photoredox step, on account of their α-C–H bonds being strengthened by ring strain and inductive effects^57^; amine **10j** for example gave only 49% yield of **13j**, along with 46% of unreacted **10j**. By increasing the stoichiometry of amines **10j** and **10k** to 3 equivalents, however, the valuable spirocyclic THNs **13j** and **13k** could be obtained in 80% and 61% yields, respectively. Non-spirocyclic THNs are also readily accessible via this methodology; isopropylamine **10l** was used, for example, to generate α,α-dimethyl-substituted THN **13l** in 75% yield. For α-monoalkylated amines (**10m**–**q**), it proved necessary to use 3.0 equivalents of the amine substrate, to mitigate against the formation of undesired dialkylated products during the photoredox α-C–H alkylation step^37^. As ethylamine **10m** is a gas at ambient pressure, it was dispensed as a 2.0 M solution in THF, affording the simple α-methylated THN **13m** in 36% yield. Ethanolamine **10o** and *N*-Boc ethylenediamine **10p** also proved to be effective substrates, generating α-hydroxymethyl- and α-aminomethyl-substituted THNs **13o** and **13p**, respectively.

We next sought to extend our automated synthesis protocol to the formation of isomeric 1,2,3,4-tetrahydro-1,6-naphthyridines (“1,6-THNs”) **14**, using 4-chloro-3-vinylpyridine **18** as a radical acceptor (Fig. 3b). Whilst the chlorinated compound **18** is far easier to access than its 4-fluoropyridine-derived^60^ counterpart, the decreased S_N_Ar reactivity of the C–Cl bond necessitated that the temperature of the flow S_N_Ar step be raised still further to 220 °C. Under these conditions, a small library of spirocyclic 1,6-THNs **14a**–**e** could be prepared in 46–64% yield.

### Gram-scale reaction and resolution of THN enantiomers

**Fig. 4:**
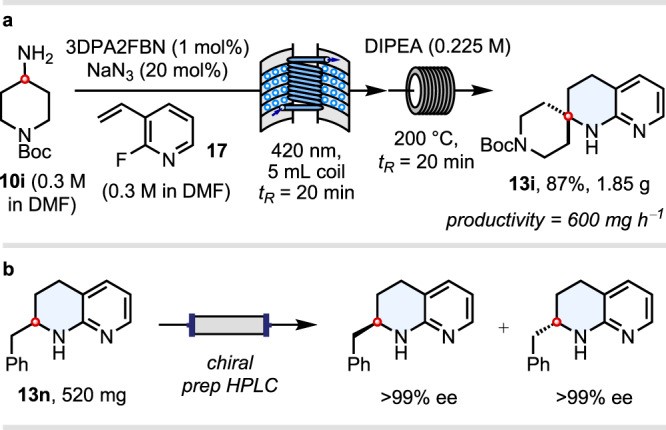
Gram-scale reaction and resolution of THN enantiomers. **a** Gram-scale reaction in flow. **b** Resolution of chiral racemic THNs by prep HPLC.

To demonstrate the scalability of our THN synthesis in flow, we executed the reaction of 4-aminopiperidine substrate **10i** on gram scale on a 5-ml reactor coil, delivering 1.85 g of spirocyclic THN **13i** in 87% yield (equating to a productivity of 600 mg h^–1^) (Fig. 4a). Whilst these reactions inevitably produce racemic materials, resolution of the THNs via chiral preparative HPLC provides convenient access to both enantiomers, as exemplified for THN **13n** on a 520 mg scale (Fig. 4b and see Supplementary Figs. 4–8 for HLPC traces).

### Access to THN derivatives with functional handles on the pyridine ring

**Fig. 5:**
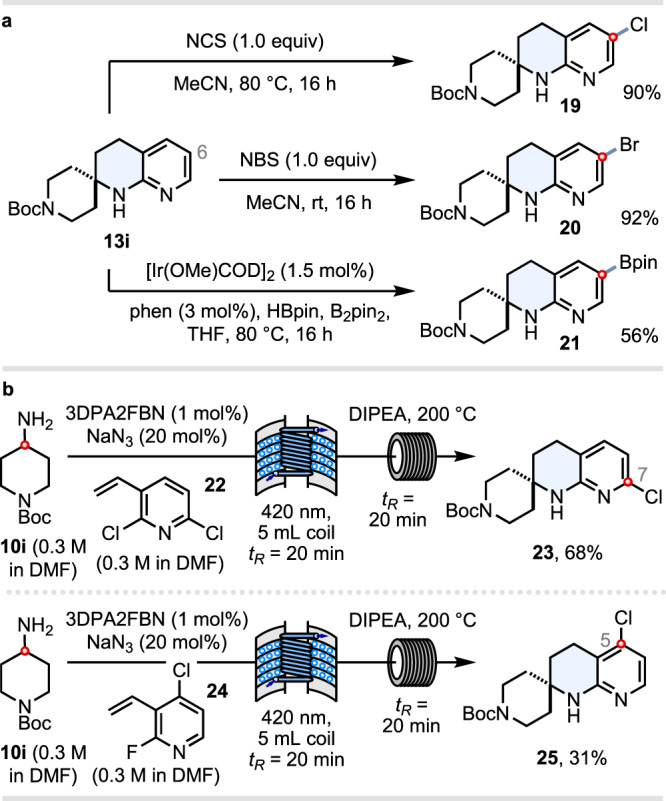
Access to THN derivatives with functional handles on the pyridine ring. **a** C–H functionalisation of the pyridine ring at C(6). **b** Access to other halogenated THN isomers.

Another important objective was to demonstrate further elaboration of the THN products on the pyridine ring. One strategy, which is especially useful for C(6) functionalisation, is to carry out electrophilic halogenation or catalytic C–H borylation^61^ reactions (i.e., **19**–**21**) (Fig. 5a). In order to access THNs **23** and **25** halogenated *ortho* or *para* to the pyridine nitrogen, we utilised vinyl pyridines **22** and **24**, respectively, with the necessary chloro handles preinstalled. Using this strategy, the C(7)-chloro THN **23** was isolated in 68% yield, and the C(5)-chloro THN **25** in 31% yield (Fig. 5b). The latter isomer was anticipated to be the most challenging, requiring the amine nucleophile to distinguish between a *para*-chloro and an *ortho*-fluoro site of attack during the S_N_Ar step^39^. Taken together, these strategies enable vector growth from any ring position on the fused pyridine moiety of 1,8-THNs, which is likely to be of significant value for fragment-based drug discovery^1–4^.

### Stepwise synthesis of other THN isomers

**Fig. 6:**
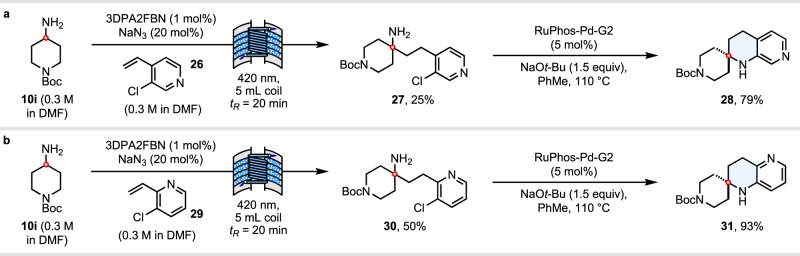
Stepwise synthesis of other THN isomers. **a** Synthesis of 1,2,3,4-tetrahydro-1,7-naphthyridines. **b** Synthesis of 1,2,3,4-tetrahydro-1,5-naphthyridines.

Varying the position of the pyridine nitrogen atom in these spirocyclic THN scaffolds is another highly desirable objective from a medicinal chemistry standpoint^9^. Having already demonstrated an automated flow synthesis of 1,8- and 1,6-THNs **13** and **14** from primary alkylamine feedstocks, we were motivated to develop a practical catalytic solution to access 1,7- and 1,5-THN isomers, based on the same photoredox-catalysed HAA disconnection approach. With intramolecular *N*-arylation via S_N_Ar no longer being feasible, we instead opted to carry out this key step using palladium catalysis. Following a flow photoredox HAA of amine **10i** with 3-chloro-4-vinylpyridine **26**, γ-pyridyl amine **27** was isolated in 25% yield. The low yield in this case was traced to extensive polymerisation side reactions, for which vinylpyridine **26** seems to be particularly prone. Subsequent cyclisation via a Buchwald-Hartwig C–N coupling then gave 1,7-THN **28** in 79% yield (Fig. 6a). An analogous sequence using 3-chloro-2-vinylpyridine **29** gave 1,5-THN in an overall 47% yield over the two steps (Fig. 6b).

### Application to the synthesis of Pfizer’s MC4R antagonist PF-07258669 5

**Fig. 7:**
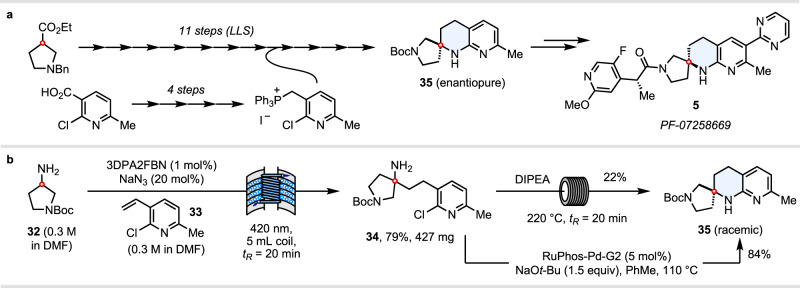
Application to synthesis of the THN core of Pfizer’s melanocortin MC4 receptor antagonist (5). **a** Prior art: Pfizer’s enantioselective synthesis of core (**35**) of melanocortin MC4 receptor antagonist **5**. **b** Our (racemic) synthesis of core (**35**) of melanocortin MC4 receptor antagonist **5**.

Finally, we sought to apply our methodology to a concise synthesis of the spirocyclic THN core (**35**) of Pfizer’s MC4R antagonist PF-07258669 **5**, which was previously synthesised in 15 total steps (11 steps LLS) (Fig. 7a)^25^. In our case, starting from commercially available 3-amino *N*-Boc pyrrolidine **32**, a photocatalytic HAA reaction with vinylpyridine **33** in continuous flow gave γ-pyridyl amine **34** (427 mg) in 79% yield. Attempted thermal S_N_Ar cyclisation of **34** at 220 °C in a high-temperature tubular reactor (*t*_R_ = 20 min) gave only 22% yield of THN **35**, indicating that the methyl substituent α- to the pyridine nitrogen deactivates this pathway. Fortunately, an intramolecular, palladium-catalysed Buchwald-Hartwig *N*-arylation process (as used in the Pfizer route) proved more efficacious, delivering the spirocyclic THN core **35** in 84% yield (Fig. 7b). Taking into account a 3-step synthesis of vinyl pyridine **33**, the longest linear sequence is five steps. The industrial route, whilst 11 steps in the longest linear sequence, is enantioselective, compared to a racemic synthesis in our case. Nevertheless, this illustrates how dramatically the synthesis of complex spirocyclic amines can be streamlined when using a photoredox annulation strategy from unprotected amines^57^.

## Conclusion

In summary, we have developed an automated, continuous flow synthesis of α-alkylated and spirocyclic 1,2,3,4-tetrahydro-1,8-naphthyridines (“1,8-THNs”), in addition to their regioisomeric 1,6-THN analogues, from abundant primary amine feedstocks. An annulative disconnection approach based on photoredox-catalysed hydroaminoalkylation (HAA) of halogenated vinylpyridines is sequenced in combination with intramolecular S_N_Ar *N*-arylation. To access the remaining 1,7- and 1,5-THN isomers, a photoredox-catalysed HAA step is telescoped with a palladium-catalysed C–N bond formation. Altogether, this provides a highly modular access to four isomeric THN cores from a common set of unprotected primary amine starting materials, using the same bond disconnections. The simplifying power of the methodology is illustrated by a concise synthesis of the spirocyclic THN core (**35**) of Pfizer’s MC4R antagonist PF-07258669 (**5**).

## Methods

A general procedure for the flow chemistry protocol described in Fig. 3 can be found in Supplementary Methods (pages S4–5), plus photographs and schematics of the setup in Supplementary Figs. 1–3.

Representative procedure for the automated continuous flow synthesis of 1,2,3,4-tetrahydro-1,8-naphthyridine (13a): following the General Procedure (pages S4–5), 5 ml of reagent feed A [2-fluoro-3-vinylpyridine **17** (185 mg, 1.50 mmol, 1.0 equiv) and 3DPA2FBN (9.6 mg, 15.0 μmol, 1 mol%) in anhydrous DMF], 5 ml of reagent feed B [cyclohexylamine **10a** (149 mg, 1.50 mmol, 1.0 equiv) and NaN_3_ (19.5 mg, 300 μmol, 20 mol%) in anhydrous DMF], and 10 ml of reagent feed C [DIPEA (291 mg, 2.25 mmol, 1.5 equiv) in anhydrous DMF] were reacted in flow, setting the high-temperature tube reactor to 180 °C. The steady-state mixture (10 ml) was collected and concentrated in vacuo on an Asynt spiral evaporator. Purification via automated flash column chromatography on SiO_2_ gel (12 g) in 40–60 °C petroleum ether (5 CV) then 100:0 → 0:100 40–60 °C petroleum ether–EtOAc (over 20 CV) then EtOAc (5 CV) gave **13a** as a colourless, crystalline solid (149 mg, 98%, productivity = 2.20 mmol h^–1^).

### Supplementary information

Supplementary Information

Description of Additional Supplementary Files

Supplementary Data 1

## Data Availability

Detailed experimental procedures and characterisation of compounds can be found in Supplementary Methods in the Supplementary Information. NMR spectra are available as a separate Supplementary Data 1. All original data are available from the authors upon request.
