# Supplementary material for: Modular, automated synthesis of spirocyclic tetrahydronaphthyridines from primary alkylamines
Source: Commun Chem. 2023 Oct 4;6:215. doi: 10.1038/s42004-023-01012-2 (PMC10550966; doi:10.1038/s42004-023-01012-2)
Supplement: Supplementary file 1 — Supplementary Information [file 42004_2023_1012_MOESM1_ESM.pdf]

Modular, Automated Synthesis of Spirocyclic Tetrahydronaphthyridines from Primary Alkylamines

Qiao Cao,[1] Joshua D. Tibbetts,[1] Gail L. Wrigley,[2] Adam P. Smalley,[3] Alexander J. Cresswell*[1]

[1]Department of Chemistry, University of Bath, Claverton Down, Bath, BA2 7AY, U.K., E-mail: a.j.cresswell@bath.ac.uk.

[2]Medicinal Chemistry, Oncology R&D, AstraZeneca, Cambridge, CB4 0WG, U.K.

[3]UCB, 216 Bath Road, Slough, SL1 3WE, U.K.

SUPPLEMENTARY INFORMATION: TABLE OF CONTENTS

Supplementary Methods *S1*

A. General Experimental *S1*

B. General Procedure for Automated, Continuous Flow Synthesis of THNs *S4*

C. Optimisation of S_NAr Step *S6*

D. Preparation of Starting Materials *S6*

E. Reaction Generality *S13*

F. Gram-Scale Synthesis of THN **13i** *S36*

G. Resolution of Chiral, Racemic THN **13n** by Prep HPLC *S37*

H. C–H Functionalisation of the Pyridine Ring of THN **13i** at C(6) *S44*

I. Access to Other Halogenated THN Isomers **23** and **25** *S48*

J. Stepwise Synthesis of 1,7- & 1,5-THNs **28** and **31** *S50*

K. Synthesis of Core (**35**) of Melanocortin MC4 Receptor Antagonist **5** *S54*

Supplementary References *S56*

Supplementary Methods

A. General Experimental

General Setup: Procedures employing oxygen- and/or moisture-sensitive materials were performed with anhydrous solvents (*vide infra*) using standard inert atmosphere techniques (atmosphere of anhydrous nitrogen or argon). Room temperature (rt) typically ranged between 20–25 °C.

Flow Chemistry Apparatus: All flow reactions were conducted on a commercially available Vapourtec RS-400 system comprising an R2C+ pump module, an R2S+ pump module (equipped with blue peristaltic pump tubes), an R4 reactor module, a GX-271 autosampler with GSIOC interface (3 × 204 sample racks, up to 27 vials each), and a touchscreen interface running Flow Commander^{TM} software. Vials used on the autosampler were Wheaton™ 20-mL glass liquid scintillation vials (Fisher, Cat. No. 10698822) equipped with Wheaton™ white polypropylene open-top caps with PTFE faced silicone liners (Fisher, Cat. No. 10119922). For high temperature reactions (up to 250 °C), a commercially available Vapourtec high-temperature tube reactor with a 10-mL stainless steel reactor coil was used.

Photoreactor: All photoredox reactions used a commercially available Uniqsis PhotoSyn HP LED photoreactor with a 420 nm LED array (716 individual diodes), which was cooled (+0.5 °C) using a Julabo FL1203 chiller. This reactor consumes ~600 W at 100% output (1400 mA) and therefore emits ~350 W at 420 nm. It was equipped with a 5-mL PFA tubing coil (1.0 mm ID) and mounted on a Uniqsis cold coil tubing module (cooled to –0.5 °C using a Huber CC-805 chiller).

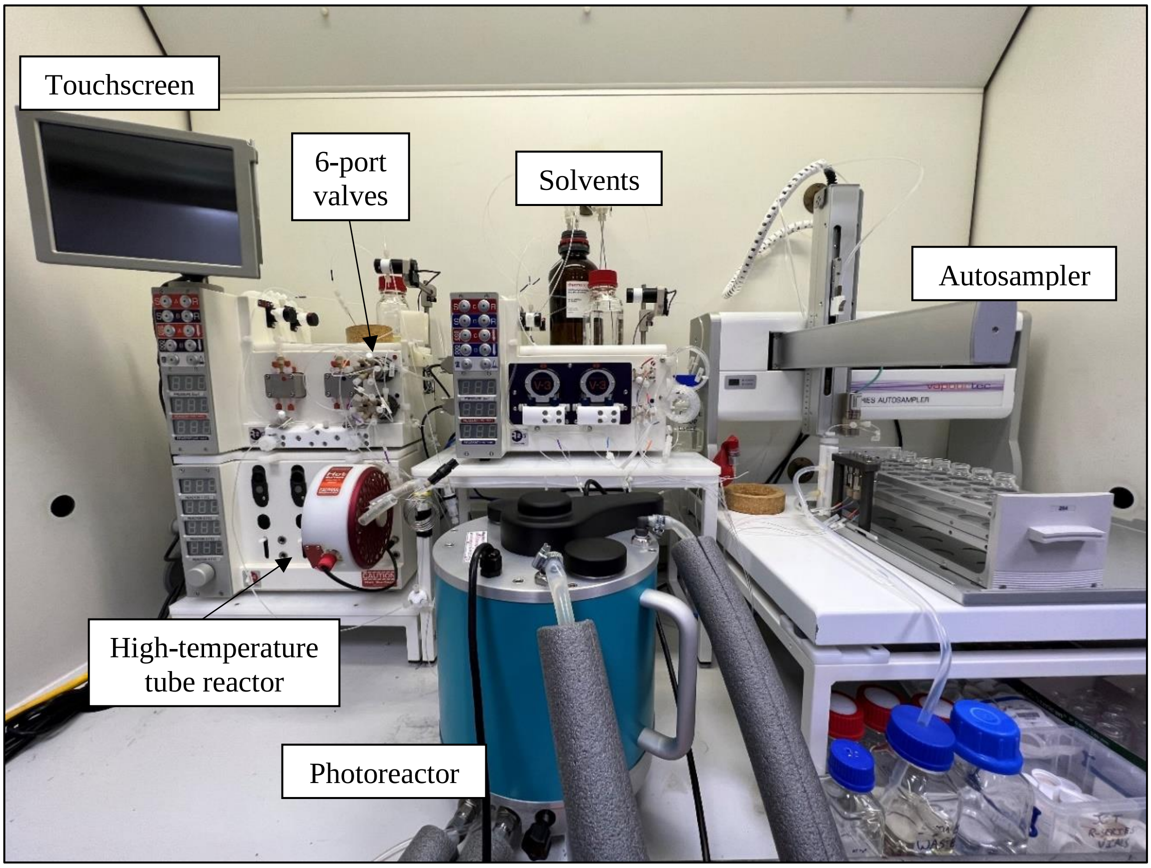

Supplementary Figure 1. Photograph of flow chemistry apparatus

NMR Spectroscopy: ^{1}H, $\{^{1}H\}^{13}C$, $\{^{1}H\}^{19}F$ and ^{13}B spectra were recorded at 400/500 MHz, 101/126 MHz, 376 MHz, and 162 MHz respectively, using a Bruker Avance spectrometer (400 MHz) or an Agilent ProPulse spectrometer equipped with a OneNMR probe (500 MHz). Spectra were recorded at 298 K (25 °C) on the Bruker instrument and 298 K (25 °C) on the Agilent instrument, unless stated otherwise. ^{1}H and $\{^{1}H\}^{13}C$ NMR spectra were referenced to residual solvent peaks. Chemical shifts are reported in parts per million (ppm) relative to residual chloroform (δ = 7.26 ppm, ^{1}H; 77.16 ppm, ^{13}C). All $\{^{1}H\}^{13}C$ resonances are assumed to be singlets, unless stated otherwise. Coupling constants, *J*, reported in Hertz (Hz), were calculated using Mestrenova to the nearest 0.1 Hz. The following abbreviations (and their combinations) are used to label the multiplicities: br (broad), s (singlet), d (doublet), t (triplet), q (quartet), sept (septet), and m (multiplet). ^{1}H and $\{^{1}H\}^{13}C$ assignments for novel compounds are corroborated by 2D NMR experiments (COSY, HSQC, HMBC).

Infrared Spectroscopy: Infrared (IR) spectra of neat compounds were recorded over the range 4000–650 cm^{-1} using a PerkinElmer Spectrum 100 ATR-FTIR spectrometer. Peaks are reported in cm^{-1} with indicated relative intensities: s (strong, 0–33% T); m (medium, 34–66% T), w (weak, 67–100% T), and br (broad).

Mass Spectrometry: Electrospray ionisation (ESI^{+}) spectra were recorded on an Agilent Electrospray Quadrupole Time-of-Flight mass spectrometer (ESI-QTOF). Data are reported in the form of *m*/*z* (intensity relative to the base peak = 100).

Melting Points: Uncorrected melting points (mp) were determined on a Stanford Research Systems OptiMelt automated capillary melting point apparatus in open capillary tubes.

Chromatography: Analytical thin-layer chromatography was performed on Merck silica gel 60 F_{254} aluminium-backed plates. Visualisation was accomplished with UV light (254 nm), iodine (I_2) on silica, aqueous basic potassium permanganate ($KMnO_4$), and/or phosphomolybdic Acid (PMA) solutions. Automated flash column chromatography (on Teledyne-Isco pre-packed SiO_2 or C_{18} reversed phase cartridges) was performed using a Teledyne-Isco CombiFlash NextGen 300+ System equipped with UV-vis and ELSD detectors. Manual flash column chromatography was performed using high-purity grade silica gel (SiO_2), pore size 60 Å, 70–230 mesh particle size (Sigma Aldrich, Cat. No. 288624).

Solvents: Reaction solvent tetrahydrofuran (THF) was dried by percolation through columns packed with neutral alumina under a positive pressure of nitrogen. Dimethylformamide (DMF) (extra dry, with molecular sieves), dichloromethane (CH_2Cl_2) and deuterated chloroform ($CDCl_3$) were used as received. Solvents for filtration, transfers, chromatography, and recrystallisation, including acetonitrile (MeCN), chloroform ($CHCl_3$), dichloromethane (CH_2Cl_2), diethyl ether (Et_2O), ethyl acetate (EtOAc), hexane, methanol (MeOH), and 40–60° petroleum ether (petrol) were used as received.

Chemicals: 4-Aminocyclohexan-1-ol (Fluorochem), 2-aminoindan (Sigma Aldrich), 1-(3-aminopropyl)imidazole (Fluorochem), bis(pinacolato)diboron [B_2pin_2] (Fluorochem), *N*-bromosuccinimide [NBS] (Acros), butyllithium [BuLi] (2.5 M in hexanes) (Sigma Aldrich), 2-chloro-6-methylnicotinic acid (Apollo Scientific), 3-chloro-2-pyridinecarboxaldehyde (Fluorochem),

4-chloro-2-fluoronicotinaldehyde (Fluorochem), 3-chloroisonicotinaldehyde (Fluorochem), 4-chloronicotinic acid (Apollo Scientific), cyclobutylamine (Fluorochem), cycloheptylamine (TCI), cyclohexylamine (Fluorochem), (1,5-cyclooctadiene)(methoxy)iridium(I) dimer {$[Ir(OMe)COD]_2$} (Sigma Aldrich), cyclopentylamine (Fluorochem), 2,6-dichloronicotinaldehyde (Apollo Scientific), *N*,*N*-diisopropylethylamine [DIPEA] (Fisher Scientific), ethanolamine (Acros), ethylamine (2 M in THF) (Sigma Aldrich), 2-fluoro-3-formylpyridine (Manchester Organics), isopropylamine (Sigma Aldrich), methyltriphenylphosphonium bromide (Fluorochem), manganese oxide [MnO_2] (Fluka), *N*-chlorosuccinimide [NCS] (Acros), oxetan-3-amine (Apollo Scientific), pinacolborane [HBpin] (Sigma Aldrich), 2-phenylethylamine (TCI), RuPhos-Pd-G2 (Sigma Aldrich), sodium azide [NaN_3] (Sigma Aldrich), sodium *tert*-butoxide [NaO*t*-Bu] (Sigma Aldrich), *tert*-butyl (aminoethyl)carbamate (Fluorochem), *tert*-butyl 3-aminoazetidine-1-carboxylate (Apollo Scientific), *tert*-butyl 3-aminopyrrolidine-1-carboxylate (Apollo Scientific), *tert*-butyl 4-aminopiperidine-1-carboxylate (Fluorochem), tetrahydro-2*H*-pyran-4-amine (Fluorochem), tetrahydro-2*H*-thiopyran-4-amine (Apollo Scientific), and 3,4,7,8-tetramethyl-1,10-phenanthroline [Me_4phen] (Sigma Aldrich) were purchased from commercial suppliers and were used as received. Photocatalyst 3DPA2FBN was prepared according to a literature procedure.[1]

Risks Associated with Sodium Azide (NaN_3)[2]

Toxicity: Sodium azide is highly toxic (comparable to soluble alkali cyanides) and should only be handled with appropriate PPE, to avoid any contact with the skin, eyes, nose, or mouth. Avoid dust formation and do not inhale (dust, vapor, mist, gas). If ingested, seek immediate medical assistance.

Explosive Potential: Thermal decomposition of NaN_3 occurs at approximately 300 °C, with formation of sodium metal and nitrogen, but other azide compounds (including transition metal azides) can detonate at much lower temperatures and be highly shock-sensitive.

Reactivity with Acids (Generation of HN_3): Treatment, accidental or otherwise, of inorganic azide sources like NaN_3 with strong mineral acids such as H_2SO_4 (even when dilute) will evolve hydrazoic acid (HN_3), a colorless, volatile, highly toxic, and explosive liquid (bp = 37 °C).

Reactivity with Metals: Inorganic azide sources like NaN_3 can react with metallic ions to precipitate metal azides, which can be shock-sensitive and explosive. Avoid (old) metal spatulas when weighing out NaN_3, or exposure of azide to metal surfaces or components (e.g., on storage).

Storage: Sodium azide or its solutions should never be stored in metal containers or with metal caps, to avoid formation of any shock-sensitive and explosive metal azide residues.

Disposal: Sodium azide or its solutions should never be directly disposed of down the drain, where highly sensitive metal azide crystals could slowly accumulate on metal plumbing (plus azide is also highly toxic to aquatic life). Adequate precautions are necessary for the safe and environmentally responsible disposal of azide solution residues. Small amounts of azide-containing waste and azide dust can be decomposed harmlessly with ceric ammonium nitrate. Larger quantities must be destroyed by addition of a sodium nitrite solution followed by a mineral acid solution.

B. General Procedure for Automated, Continuous Flow Synthesis of THNs

The flow system was set up as shown below, with each amine substrate **10** (along with NaN_3 catalyst) pre-dissolved in DMF and loaded in 20-mL scintillation vials in the autosampler, along with the requisite number of empty vials to collect each individual product **13**. Pumps A and B2 are peristaltic pumps (with blue tubing) on the R2S+ pump module, pump B1 is a syringe pump associated with the autosampler, and pump C is a stainless steel HPLC pump on the R2C+ pump module. Using Vapourtec's Flow Commander^{TM} software, it was calculated that an injection volume of at least 3.5 mL of reagent feed B into the sample loop was necessary to ensure collection of a steady-state product, so we opted to inject 5.0 mL per experiment, in order to fill the sample loop to capacity.

The diagram below illustrates the use of 2-fluoro-3-vinylpyridine **17** in the synthesis of THNs **13**, but the set up was identical when using other vinylpyridines (e.g., **18**). For single reactions (without an autosampler), the amine **10** + NaN_3 solution was instead pumped directly from a volumetric flask using pump B2. For reactions without a heated (S_NAr) step, the output of the photoreactor was connected directly to the BPR, and the product was collected using a switch valve.

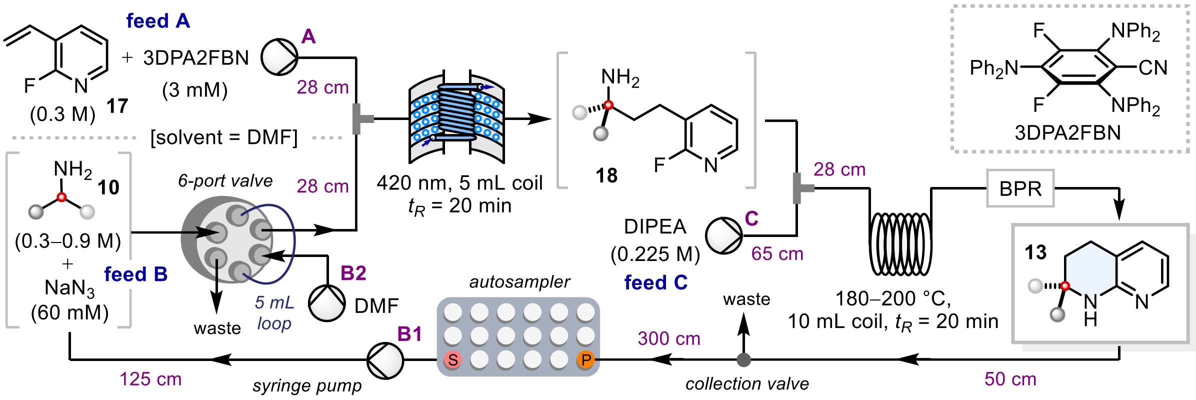

Supplementary Figure 2. Flow chemistry set-up and tubing lengths

The following reagent feeds were used, with all solvent lines for pumps A, B2, and C being fed from a reservoir of DMF:

Feed A: A solution of the requisite vinylpyridine (7.50 mmol, 1.0 equiv, 0.30 M) and 3DPA2FBN (0.075 mmol, 1 mol%, 3 mM) in anhydrous DMF.

Feed B: A 10-mL solution of the requisite amine **10** (3.00 mmol, 1.0 equiv, 0.3 M or 9.00 mmol, 3.0 equiv, 0.9 M) and NaN_3 (39.0 mg, 0.60 mmol, 20 mol%, 0.06 M) in anhydrous DMF was prepared in a 10-mL volumetric flask. Following sonification for 5 min (to dissolve the NaN_3), the solution was transferred to a capped 20-mL scintillation vial and loaded onto the autosampler.

Feed C: A 0.225 M solution of *N*,*N*-diisopropylethylamine (DIPEA) in anhydrous DMF.

With the solution feeds loaded onto the system, as per the above diagram, the reagent lines for feeds A and C were primed and the rest of the system was flushed with DMF. The Uniqsis PhotoSyn HP LED

photoreactor, mounted on the cold coil tubing module, was set at 75% power (~260 W at 420 nm). Using the Flow Commander™ software to program a multi-experiment run, a 6.5 mL portion of reagent feed B was first transferred into the 5-mL sample loop from the autosampler. This was then injected into the 5-mL coil of the photoreactor (*via* the T-mixer) along with a 5-mL portion of reagent feed A, each at a flow rate of 0.125 mL min^{-1} (residence time = 20 min). Under the control of the software, an 8.5-mL portion of reagent feed C was injected at a flow rate of 0.250 mL min^{-1} to intercept the output stream from the photoreactor. After passing through another T-mixer, the combined flow stream entered the 10-mL high-temperature tube reactor at either 180, 200, or 220 °C, depending on the substrates used (residence time = 20 min). The pressure was maintained below 8 bar using a back-pressure regulator (BPR). *[**Note**: A preset BPR containing a wetted metal spring*[3] *should be avoided, due to the risk of accumulating metal azide residues. Instead, we recommend using an adjustable BPR based on constriction of flow with a pressurised diaphragm. In the event of BPR blockage, we recommend cleaning with H_2O and 0.5 M aq. HCl].* Once reagent feeds A and B had fully injected, the system automatically switched to pumping DMF on both solvent lines A and B2. Similarly, pump C switched to pumping DMF once the reaction mixture had been fully injected into the high temperature reactor. Each steady-state product mixture (12.0 mL) was collected in a 20-mL vial by the autosampler (serving now as a fraction collector). After concentration *in vacuo*, each crude product was purified *via* automated flash column chromatography. Each run using 1.50 mmol of the vinylpyridine substrate takes approximately 90 min, which corresponds to 16 compounds in a 24 h period (or 40 compounds total over 60 h if all rack positions are utilised).

For clarity, the 6-port valve was configured as shown below:

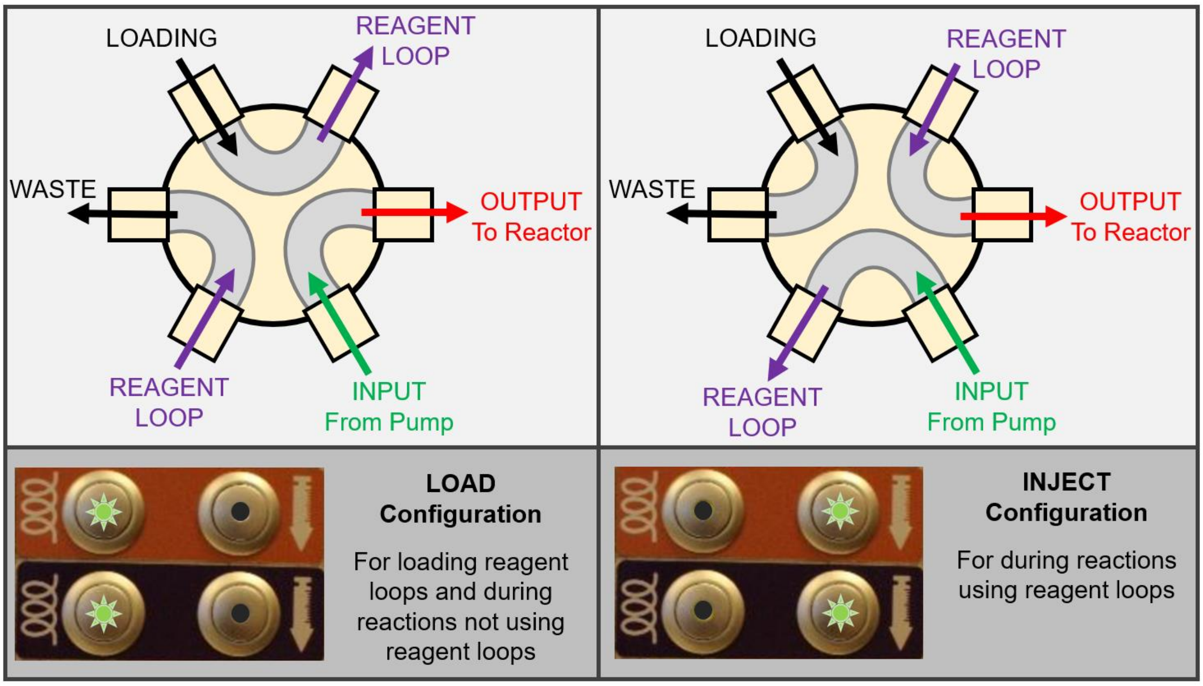

Supplementary Figure 3. Schematic of 6-port valve

C. Optimisation of S_NAr Step

Entry	Temp. (°C)	18a (%)	13a (%)
1	150	44	56
2	160	36	64
3	170	20	80
4	180	<1	>99
5	190	<1	>99
6	200	<1	>99
7	210	<1	>99

Supplementary Table 1. Optimisation of S_NAr step in continuous flow

D. Preparation of Starting Materials

Preparation of 2-fluoro-3-vinylpyridine (17)

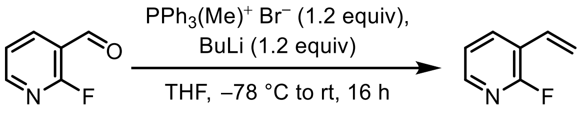

17

A 500-mL, 3-necked RBF equipped with a magnetic stirrer bar, two rubber septa, and a N_2 inlet was flame-dried then left to cool under a stream of N_2. The middle neck was briefly unsealed to charge the flask with methyltriphenylphosphonium bromide (16.40 g, 46.0 mmol, 1.2 equiv), under a flow of N_2 gas, and the septum was replaced. After cycling between N_2 gas and vacuum (three times), anhydrous THF (380 mL) was then added *via* syringe. Stirring was commenced and the colourless solution was cooled in a dry ice/acetone bath (–78 °C), followed by dropwise addition of BuLi (2.5 M in hexanes, 18.4 mL, 46.0 mmol, 1.2 equiv). The colour gradually changed to yellow. The mixture was stirred for 40 min in the dry ice/acetone bath and 2-fluoro-3-formylpyridine (4.80 g, 38.4 mmol, 1.0 equiv) was then added dropwise and a dark orange solution was obtained. The mixture was then allowed to warm to rt over 16 h. H_2O (5 mL) was added to quench the reaction, followed by concentration *in vacuo*. The residue was re-dissolved in EtOAc (50 mL) and washed with sat. brine (2 × 50 mL). The volume of EtOAc was reduced to ca. 5 mL by concentration *in vacuo* and the crude material was cooled in an ice-water bath (0 °C), with rapid stirring provided by a magnetic stirrer bar. Pre-cooled (0 °C) 40–60 °C petroleum ether (450 mL) was added rapidly in one portion *via* a wide-neck funnel to precipitate $Ph_3P{=}O$ residues, which were removed *via* filtration. Following concentration *in vacuo*, purification *via* manual flash column chromatography (12 cm depth SiO_2 gel, 58 mm diameter column) in 97:0:3→68:29:3 40–60 °C petroleum ether–EtOAc–Et_3N gave **17** as a pale yellow oil (2.96 g, 60%). ^1H NMR (400 MHz, $CDCl_3$): 8.06 (dt, J = 5.0, 1.7 Hz, 1H), 7.16 (ddd, J = 7.5, 5.0, 1.7 Hz, 1H), 7.14 (ddd, J = 7.5, 5.0, 1.7 Hz, 1H), 6.74 (dd, J = 17.8, 11.2 Hz, 1H), 5.86 (dd, J = 17.8, 0.8 Hz, 1H), 5.45 (dd, J = 11.2, 0.8 Hz, 1H). {^1H}^{19}F NMR (376 MHz, $CDCl_3$): –71.9. *The obtained analytical data were in full agreement with the literature.*[4]

Preparation of 4-chloro-3-vinylpyridine (18)

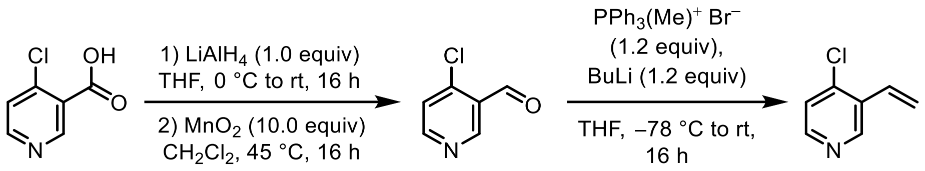

A 250-mL, 3-necked RBF equipped with a magnetic stirrer bar, two rubber septa, and a N_2 inlet was flame-dried then left to cool under a stream of N_2. The middle neck was briefly unsealed to charge the flask with 4-chloronicotinic acid (7.00 g, 44.4 mmol, 1.0 equiv) and THF (100 mL) and the mixture was cooled in an ice-water bath (0 °C), followed by portion-wise addition of $LiAlH_4$ (1.69 g, 44.4 mmol, 1.0 equiv) over 20 min. The resultant orange mixture was stirred at rt for 16 h, then quenched with sat. $NaHCO_3$ (10 mL) and the resultant slurry was filtered through a 5-cm pad of Celite® on a medium porosity frit filter. The filtrate was concentrated *in vacuo* and the residue was dissolved in EtOAc (30 mL) and washed with H_2O (20 mL). The aqueous phase was extracted with EtOAc (3 × 30 mL) and the combined organic phases were dried over $MgSO_4$. Concentration *in vacuo* gave crude (4-chloropyridin-3-yl)methanol as a yellow solid (3.17 g). The material was used directly for the next step without purification. Data for (4-chloropyridin-3-yl)methanol: ^{1}H NMR (400 MHz, $CDCl_3$): 8.66 (s, 1H), 8.42 (d, J = 5.4 Hz, 1H), 7.31 (d, J = 5.4 Hz, 1H), 4.83 (s, 2H). *The obtained analytical data were in full agreement with the literature.*[5]

A 100-mL RBF equipped with a magnetic stirrer bar and a water-cooled condenser was charged with crude (4-chloropyridin-3-yl)methanol from the previous step (3.17 g, 22.1 mmol, 1.0 equiv), MnO_2 (19.2 g, 0.22 mol, 10.0 equiv) and CH_2Cl_2 (50 mL). The mixture was refluxed at 45 °C for 16 h, filtered through a pad of Celite®, and concentrated *in vacuo*. Purification *via* automated flash column chromatography on SiO_2 gel (12 g) in 97:3 40–60 °C petroleum ether–Et_3N (5 CV) then 97:0:3→68:29:3 40–60 °C petroleum ether–EtOAc–Et_3N (over 15 CV) then 68:29:3 40–60 °C petroleum ether–EtOAc–Et_3N (5 CV) gave 4-chloronicotinaldehyde as a colourless solid (1.40 g, 22% over 2 steps). Data for 4-chloronicotinaldehyde: ^{1}H NMR (400 MHz, $CDCl_3$): 10.45 (s, 1H), 8.98 (s, 1H), 8.63 (d, J = 5.4 Hz, 1H), 7.39 (d, J = 5.4 Hz, 1H). *The obtained analytical data were in full agreement with the literature.*[6]

A 250-mL, 3-necked RBF equipped with a magnetic stirrer bar, two rubber septa, and a N_2 inlet was flame-dried then left to cool under a stream of N_2. The middle neck was briefly unsealed to charge the flask with methyltriphenylphosphonium bromide (4.35 g, 12.2 mmol, 1.25 equiv), under a flow of N_2 gas, and the septum was replaced. After cycling between N_2 gas and vacuum (three times), anhydrous THF (125 mL) was then added *via* syringe. Stirring was commenced and the colourless solution was cooled in a dry ice/acetone bath (–78 °C), followed by dropwise addition of BuLi (2.5 M in hexanes,

4.70 mL, 11.7 mmol, 1.2 equiv). The colour gradually changed to yellow. The mixture was stirred for 40 min in the dry ice/acetone bath and 4-chloronicotinaldehyde (1.38 g, 9.75 mmol. 1.0 equiv) was then added dropwise. The mixture was allowed to warm to rt over 16 h and was quenched by addition of H_2O (5 mL), followed by concentration *in vacuo*. The residue was re-dissolved in EtOAc (30 mL), washed with sat. brine (2 × 30 mL), dried over Na_2SO_4, then concentrated *in vacuo*. Purification *via* automated flash column chromatography on SiO_2 gel (12 g) in 97:3 40–60 °C petroleum ether–Et_3N (5 CV) then 97:0:3→68:29:3 40–60 °C petroleum ether–EtOAc–Et_3N over (15 CV) then 68:29:3 40–60 °C petroleum ether–EtOAc–Et_3N (5 CV) gave **18** as a pale yellow oil (642 mg, 48%). Data for **18**: ^{1}H NMR (400 MHz, $CDCl_3$): 8.67 (s, 1H), 8.30 (d, *J* = 5.4 Hz, 1H), 7.23 (d, *J* = 5.4 Hz, 1H), 6.91 (dd, *J* = 17.6, 11.2 Hz, 1H), 5.78 (dd, *J* = 17.6, 1.0 Hz, 1H), 5.43 (dd, *J* = 11.2, 1.0 Hz, 1H). HRMS (ESI$^+$): Calcd for C_7H_6ClN: 139.0189, found 139.0191. *The obtained analytical data were in full agreement with the literature.*[6]

Preparation of 2,6-dichloro-3-vinylpyridine (22)

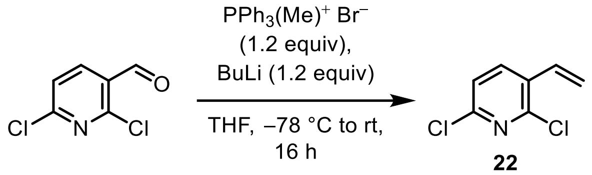

A 250-mL, 3-necked RBF equipped with a magnetic stirrer bar, two rubber septa, and a N_2 inlet was flame-dried then left to cool under a stream of N_2. The middle neck was briefly unsealed to charge the flask with methyltriphenylphosphonium bromide (3.65 g, 10.2 mmol, 1.20 equiv), under a flow of N_2 gas, and the septum was replaced. After cycling between N_2 gas and vacuum (three times), anhydrous THF (125 mL) was then added *via* syringe. Stirring was commenced and the colourless solution was cooled in a dry ice/acetone bath (–78 °C), followed by dropwise addition of BuLi (2.5 M in hexanes, 4.10 mL, 10.2 mmol, 1.2 equiv). The colour gradually changed to yellow. The mixture was stirred for 30 min in the dry ice/acetone bath and 2,6-dichloronicotinaldehyde (1.50 g, 8.52 mmol. 1.0 equiv) was then added dropwise. The mixture was allowed to warm to rt over 16 h and was quenched by addition of H_2O (5 mL), followed by concentration *in vacuo*. The residue was re-dissolved in EtOAc (30 mL), washed with sat. brine (30 mL), dried over Na_2SO_4, then concentrated *in vacuo*. Purification *via* automated flash column chromatography on SiO_2 gel (12 g) in 99:0:1 40–60 °C petroleum ether–EtOAc–Et_3N (5 CV) then 99:9:1→90:9:1 40–60 °C petroleum ether–EtOAc–Et_3N (over 15 CV) then 90:9:1 40–60 °C petroleum ether–EtOAc–Et_3N (5 CV) gave **22** as a colourless, amorphous solid (1.07 g, 72%). Data for **22**: ^{1}H NMR (400 MHz, $CDCl_3$): 7.82 (d, *J* = 7.8 Hz, 1H), 7.30 (d, *J* = 7.8 Hz, 1H), 6.97 (dd, *J* = 17.5, 11.1 Hz, 1H), 5.78 (dd, *J* = 17.5 Hz, 1H), 5.52 (dd, *J* = 11.1 Hz, 1H). $^{13}C\{^1H\}$ NMR (101 MHz, $CDCl_3$): 149.1, 148.8, 137.2, 131.2, 131.0, 123.4, 119.3. HRMS (ESI$^+$): Calcd for $C_7H_5Cl_2N$: 172.9799, found: 172.9805.

Preparation of 4-chloro-2-fluoro-3-vinylpyridine (24)

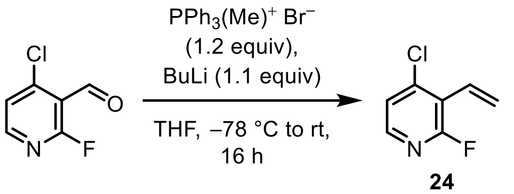

A 250-mL, 3-necked RBF equipped with a magnetic stirrer bar, two rubber septa, and a N_2 inlet was flame-dried then left to cool under a stream of N_2. The middle neck was briefly unsealed to charge the flask with methyltriphenylphosphonium bromide (2.69 g, 7.52 mmol, 1.20 equiv), under a flow of N_2 gas, and the septum was replaced. After cycling between N_2 gas and vacuum (three times), anhydrous THF (125 mL) was then added *via* syringe. Stirring was commenced and the colourless solution was cooled in a dry ice/acetone bath (–78 °C), followed by dropwise addition of BuLi (2.5 M in hexanes, 2.80 mL, 6.89 mmol, 1.1 equiv). The colour gradually changed to yellow. The mixture was stirred for 30 min in the dry ice/acetone bath and 4-chloro-2-fluoronicotinaldehyde (1.00 g, 6.27 mmol. 1.0 equiv) was then added dropwise. The resultant brown mixture was allowed to warm to rt over 16 h and was quenched by addition of H_2O (5 mL), followed by concentration *in vacuo*. The residue was re-dissolved in EtOAc (30 mL), washed with sat. brine (30 mL), dried over Na_2SO_4, then concentrated *in vacuo*. Purification *via* automated flash column chromatography on SiO_2 gel (12 g) in 99:0:1 40–60 °C petroleum ether–EtOAc–Et_3N (5 CV) then 99:9:1→90:9:1 40–60 °C petroleum ether–EtOAc–Et_3N (over 15 CV) then 90:9:1 40–60 °C petroleum ether–EtOAc–Et_3N (5 CV) gave **24** as a pale yellow oil (281 mg, 29%). Data for **24**: ^{1}H NMR (400 MHz, $CDCl_3$): 7.95 (d, J = 5.3 Hz, 1H), 7.22 (d, J = 5.3 Hz, 1H), 6.80 (dd, J = 17.8, 11.9 Hz, 1H), 6.10 (d, J = 17.8 Hz, 1H), 5.72 (d, J = 11.9 Hz, 1H). ^{13}C{^{1}H} NMR (101 MHz, $CDCl_3$): 161.8, 145.2, 145.0, 125.6, 124.0, 123.2, 119.4. {^{1}H}^{19}F NMR (376 MHz $CDCl_3$): –65.2. HRMS (ESI$^+$): Calcd for C_7H_5ClFN: 157.0094, found: 157.0090.

Preparation of 3-chloro-4-vinylpyridine (26)

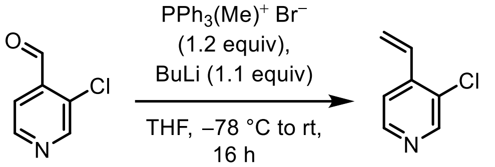

A 250-mL, 3-necked RBF equipped with a magnetic stirrer bar, two rubber septa, and a N_2 inlet was flame-dried then left to cool under a stream of N_2. The middle neck was briefly unsealed to charge the flask with methyltriphenylphosphonium bromide (4.54 g, 12.7 mmol, 1.20 equiv), under a flow of N_2 gas, and the septum was replaced. After cycling between N_2 gas and vacuum (three times), anhydrous THF (125 mL) was then added *via* syringe. Stirring was commenced and the colourless solution was

cooled in a dry ice/acetone bath (–78 °C), followed by dropwise addition of BuLi (2.5 M in hexanes, 4.70 mL, 11.7 mmol, 1.1 equiv). The colour gradually changed to yellow. The mixture was stirred for 30 min in the dry ice/acetone bath and 3-chloroisonicotinaldehyde (1.50 g, 10.6 mmol. 1.0 equiv) was then added dropwise. The resultant brown mixture was allowed to warm to rt over 16 h and was quenched by addition of H_2O (5 mL), followed by concentration *in vacuo*. The residue was re-dissolved in EtOAc (30 mL), washed with sat. brine (30 mL), dried over Na_2SO_4, then concentrated *in vacuo*. Purification *via* automated flash column chromatography on SiO_2 gel (12 g) in 99:0:1 40–60 °C petroleum ether–EtOAc–Et_3N (5 CV) then 99:9:1→90:9:1 40–60 °C petroleum ether–EtOAc–Et_3N (over 15 CV) then 90:9:1 40–60 °C petroleum ether–EtOAc–Et_3N (5 CV) gave **26** as a pale yellow oil (928 mg, 62%). Data for **26**: ^1H NMR (400 MHz, $CDCl_3$): 8.54 (s, 1H), 8.42 (d, J = 5.2 Hz, 1H), 7.40 (d, J = 5.2 Hz, 1H), 7.02 (dd, J = 17.6, 11.0 Hz, 1H), 5.96 (d, J = 17.6 Hz, 1H), 5.59 (d, J = 11.0 Hz, 1H). $^{13}C\{^1H\}$ NMR (101 MHz, $CDCl_3$): 150.2, 147.8, 142.7, 131.2, 130.5, 120.9, 120.3. HRMS (ESI^+): Calcd for C_7H_6ClN: 139.0189, found: 139.0192. *The obtained analytical data were in full agreement with the literature.*[7]

Preparation of 3-chloro-2-vinylpyridine (29)

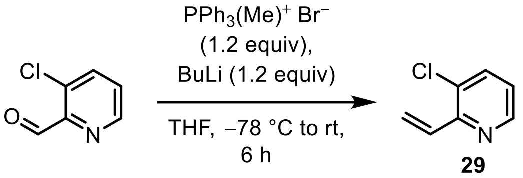

A 250-mL, 3-necked RBF equipped with a magnetic stirrer bar, two rubber septa, and a N_2 inlet was flame-dried then left to cool under a stream of N_2. The middle neck was briefly unsealed to charge the flask with methyltriphenylphosphonium bromide (3.00 g, 8.4 mmol, 1.2 equiv), under a flow of N_2 gas, and the septum was replaced. After cycling between N_2 gas and vacuum (three times), anhydrous THF (60 mL) was then added *via* syringe. Stirring was commenced and the colourless solution was cooled in a dry ice/acetone bath (–78 °C), followed by dropwise addition of BuLi (2.5 M in hexanes, 3.40 mL, 8.40 mmol, 1.2 equiv). The colour gradually changed to yellow. The mixture was stirred for 30 min in the dry ice/acetone bath and 3-chloro-2-pyridinecarboxaldehyde (0.99 g, 7.0 mmol, 1.0 equiv) was then added dropwise. The mixture stirred at –78 °C for 2 h and then allowed to warm to rt over a further 4 h. It was then re-cooled in an ice-water bath (0 °C) and quenched by addition of H_2O (10 mL), then concentrated *in vacuo*. The residue was re-dissolved in EtOAc (30 mL), washed with sat. brine (30 mL), then dried over Na_2SO_4. The volume of EtOAc was reduced to ca. 3 mL by concentration *in vacuo* and the crude material was cooled in an ice-water bath (0 °C), with rapid stirring provided by a magnetic stirrer bar. Pre-cooled (0 °C) 40–60 °C petroleum ether (50 mL) was added rapidly in one portion *via* a wide-neck funnel to precipitate $Ph_3P{=}O$ residues, which were removed *via* filtration. Following

concentration *in vacuo*, purification *via* automated flash column chromatography on SiO_2 gel (12 g) in 99:1:0.025 pentane–EtOAc–Et_3N (5 CV) then 99:1:0.025→80:20:0.5 pentane–EtOAc–Et_3N (over 15 CV) then 80:20:0.5 pentane–EtOAc–Et_3N (5 CV) gave **29** as a pale yellow oil (298 mg, 25%). Data for **29**: ^{1}H NMR (400 MHz, $CDCl_3$) δ 8.48 (d, *J* = 4.4 Hz, 1H), 7.66 (d, *J* = 8.1 Hz, 1H), 7.30–7.20 (dd, *J* = 10.8, 17.0 Hz, 2H), 7.13 (dd, *J* = 8.1, 4.4 Hz, 1H), 6.48 (dd, *J* = 17.0, 2.0 Hz, 1H), 5.60 (dd, *J* = 10.8, 2.0 Hz, 1H). ^{13}C{^{1}H} NMR (101 MHz, $CDCl_3$) δ 152.1, 147.6, 137.5, 131.6, 130.5, 123.5, 121.4. HRMS (ESI$^+$): Calcd for $[M+H]^+$ for C7H6ClN: 140.0262, found: 140.0279. *The obtained analytical data were in full agreement with the literature.*[8]

Preparation of 2-chloro-6-methyl-3-vinylpyridine (33)

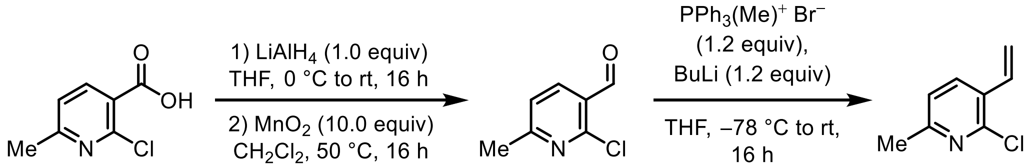

33

A 250-mL, 3-necked RBF equipped with a magnetic stirrer bar, two rubber septa, and a N_2 inlet was flame-dried then left to cool under a stream of N_2. The middle neck was briefly unsealed to charge the flask with 2-chloro-6-methylnicotinic acid (6.40 g, 37.3 mmol, 1.0 equiv) and THF (100 mL) and the mixture was cooled in an ice-water bath (0 °C), followed by portion-wise addition of $LiAlH_4$ (1.42 g, 37.3 mmol, 1.0 equiv) over 20 min. The resultant orange mixture was stirred at rt for 16 h, then quenched with sat. $NaHCO_3$ (10 mL) and the resultant slurry was filtered through a 5-cm pad of Celite® on a medium porosity frit filter. The filtrate was concentrated *in vacuo* and the residue was dissolved in EtOAc (50 mL) and washed with H_2O (40 mL). The aqueous phase was extracted with EtOAc (3 × 50 mL) and the combined organic phases were dried over $MgSO_4$. Concentration *in vacuo* gave crude (2-chloro-6-methylpyridin-3-yl)methanol as a yellow solid (4.52 g, 77%). The material was used directly for the next step without purification. ^{1}H NMR (400 MHz, $CDCl_3$): 7.74 (d, *J* = 7.8 Hz, 1H), 7.09 (d, *J* = 7.8 Hz, 1H), 4.72 (s, 2H), 2.50 (s, 1H). ^{13}C NMR (101 MHz, $CDCl_3$): 157.9, 148.3, 137.5, 132.0, 122.4, 61.3, 23.7. HRMS (ESI$^+$): Calcd for C_7H_8ClNO: 157.0294, found: 157.0298.

A 100-mL RBF equipped with a magnetic stirrer bar and a water-cooled condenser was charged with crude (2-chloro-6-methylpyridin-3-yl)methanol (2.00 g, 12.7 mmol, 1.0 equiv), MnO_2 (11.1 g, 0.13 mol, 10.0 equiv) and CH_2Cl_2 (50 mL). The mixture was refluxed at 45 °C for 16 h, filtered through a pad of Celite®, and concentrated *in vacuo*. Purification *via* automated flash column chromatography on SiO_2 gel (12 g) (40–60 °C petroleum ether–Et_3N 97:3 (5 CV) then 97:0:3→90:9:1 40–60 °C petroleum ether–EtOAc–Et_3N (over 15 CV) then 90:9:1 40–60 °C petroleum ether–EtOAc–Et_3N (5 CV)) gave 2-chloro-6-methylnicotinaldehyde as a colourless solid (1.32 g, 67%). Data for 2-chloro-6-methylnicotinaldehyde: ^{1}H NMR (400 MHz, $CDCl_3$): 10.39 (s, 1H), 8.11 (d, *J* = 7.8 Hz, 1H), 7.24 (d,

J = 7.8 Hz, 1H), 2.62 (s, 3H). ^{13}C NMR (101 MHz, $CDCl_3$): 189.2, 165.4, 153.2, 138.3, 126.6, 123.9, 24.9. HRMS (ESI$^+$): Calcd for C_7H_6ClNO: 155.0138, found: 155.0138.

A 250-mL, 3-necked RBF equipped with a magnetic stirrer bar, two rubber septa, and a N_2 inlet was flame-dried then left to cool under a stream of N_2. The middle neck was briefly unsealed to charge the flask with methyltriphenylphosphonium bromide (3.64 g, 10.2 mmol, 1.20 equiv), under a flow of N_2 gas, and the septum was replaced. After cycling between N_2 gas and vacuum (three times), anhydrous THF (75 mL) was then added *via* syringe. Stirring was commenced and the colourless solution was cooled in a dry ice/acetone bath (–78 °C), followed by dropwise addition of BuLi (2.5 M in hexanes, 4.1 mL, 10.2 mmol, 1.2 equiv). The colour gradually changed to yellow. The mixture was stirred for 30 min in the dry ice/acetone bath and 2-chloro-6-methylnicotinaldehyde (1.32 g, 8.50 mmol. 1.00 equiv) was then added dropwise. The mixture was allowed to warm to rt over 16 h and was quenched by addition of H_2O (10 mL), followed by concentration *in vacuo*. The residue was re-dissolved in EtOAc (30 mL), washed with sat. brine (30 mL), dried over Na_2SO_4, then concentrated *in vacuo*. Purification *via* automated flash column chromatography on SiO_2 gel (12 g) in 97:3 40–60 °C petroleum ether–Et_3N (5 CV) then 97:0:3→90:9:1 40–60 °C petroleum ether–EtOAc–Et_3N (over 15 CV) then 90:9:1 40–60 °C petroleum ether–EtOAc–Et_3N (5 CV) gave **33** as a pale yellow oil (919 mg, 71%). ^{1}H NMR (400 MHz, $CDCl_3$): 7.75 (d, *J* = 7.8 Hz, 1H), 7.07 (d, *J* = 7.8 Hz, 1H), 6.99 (dd, *J* = 17.6, 11.0 Hz, 1H), 5.72 (dd, *J* = 17.6, 1.0 Hz, 1H), 5.42 (dd, *J* = 11.2, 1.0 Hz, 1H), 2.52 (s, 3H). ^{13}C NMR (101 MHz, $CDCl_3$): 158.5, 149.0, 135.3, 132.0, 129.3, 122.5, 117.7, 24.0. HRMS (ESI$^+$): Calcd for C_8H_8ClN: 153.0345, found: 153.0352.

E. Reaction Generality

Preparation of 3',4'-dihydro-1'*H*-spiro[cyclohexane-1,2'-[1,8]naphthyridine] (13a)

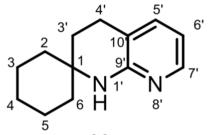

13a

Following the **General Procedure** (section B), 5 mL of reagent feed A [2-fluoro-3-vinylpyridine **17** (185 mg, 1.50 mmol, 1.0 equiv) and 3DPA2FBN (9.6 mg, 15.0 µmol, 1 mol%) in anhydrous DMF], 5 mL of reagent feed B [cyclohexylamine **10a** (149 mg, 1.50 mmol, 1.0 equiv) and NaN_3 (19.5 mg, 300 µmol, 20 mol%) in anhydrous DMF], and 10 mL of reagent feed C [DIPEA (291 mg, 2.25 mmol, 1.5 equiv) in anhydrous DMF] were reacted in flow, setting the high-temperature tube reactor to 180 °C. The steady state mixture (10 mL) was collected and concentrated *in vacuo* on an Asynt spiral evaporator. Purification *via* automated flash column chromatography on SiO_2 gel (12 g) in 40–60 °C petroleum ether (5 CV) then 100:0→0:100 40–60 °C petroleum ether–EtOAc (over 20 CV) then EtOAc (5 CV) gave **13a** as a colourless, crystalline solid (149 mg, 98%, 2.20 mmol h^{-1}). *The obtained analytical data were in full agreement with the literature.*[1]

Data for **13a**:

mp: 118–120 °C

^{1}H NMR: (400 MHz, $CDCl_3$)

7.86 (dd, *J* = 5.0, 1.7 Hz, 1H, C(7′)*H*), 7.16 (dd, *J* = 7.2, 1.7 Hz, 1H, C(5′)*H*), 6.47 (dd, *J* = 7.2, 5.0 Hz, 1H. C(6′)*H*), 5.00 (s, 1H, N*H*), 2.70 (t, *J* = 6.7 Hz, 2H, C(4′)H_2), 1.70 (t, *J* = 6.7 Hz, 2H, C(3′)H_2), 1.59–1.35 (m, 10H, C(2)H_2, C(3)H_2, C(4)H_2, C(5)H_2, C(6)H_2)

^{13}C NMR: (101 MHz $CDCl_3$)

155.7 (*C*(9′)), 146.2 (*C*(7′)), 136.2 (*C*(5′)), 115.6 (*C*(10′)), 112.5 (*C*(6′)), 51.2 (*C*(1)), 37.8 (*C*(2), *C*(6)), 32.4 (*C*(3′)), 26.0 (*C*(4)), 22.9 (*C*(4′)), 21.9 (*C*(3), *C*(5))

IR: (neat)

3248 (w), 2927 (m), 2850 (w), 1601 (m), 1581 (w), 1518 (m), 1451 (m), 1435 (m), 1354 (w), 1342 (w), 1324 (w), 1291 (w), 1278 (w), 1263 (w), 1247 (w), 1233 (w), 1193 (w), 1178 (w), 1168 (w), 1125 (w), 1105 (w), 1092 (w), 912 (w), 899 (w), 760 (m), 732 (w)

HRMS: (ESI^+)

Calcd for $C_{13}H_{19}N_2$: 203.1548, found: 203.1547

Preparation of 3′,4′-dihydro-1′*H*-spiro[cycloheptane-1,2′-[1,8]naphthyridine] (13b)

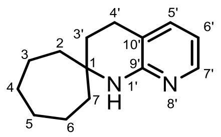

13b

Following the **General Procedure** (section B), 5 mL of reagent feed A [2-fluoro-3-vinylpyridine **17** (185 mg, 1.50 mmol, 1.0 equiv) and 3DPA2FBN (9.6 mg, 15.0 µmol, 1 mol%) in anhydrous DMF], 5 mL of reagent feed B [cycloheptylamine **10b** (170 mg, 1.50 mmol, 1.0 equiv) and NaN_3 (19.5 mg, 300 µmol, 20 mol%) in anhydrous DMF], and 10 mL of reagent feed C [DIPEA (291 mg, 2.25 mmol, 1.5 equiv) in anhydrous DMF] were reacted in flow, setting the high-temperature tube reactor to 180 °C. The steady state mixture (10 mL) was collected and concentrated *in vacuo* on an Asynt spiral evaporator. Purification *via* automated flash column chromatography on SiO_2 gel (12 g) in 40–60 °C petroleum ether (5 CV) then 100:0→0:100 40–60 °C petroleum ether–EtOAc (over 20 CV) then EtOAc (5 CV) gave **13b** as a pale yellow, crystalline solid (163 mg, 83%, 1.86 mmol h^{-1}).

Data for **13b**:

mp: 116–118 °C

^{1}H NMR: (400 MHz, $CDCl_3$)

7.86 (dd, *J* = 5.0, 1.9 Hz, 1H, C(7′)*H*), 7.17 (dd, 1H, *J* = 7.2, 1.9 Hz, C(5′)*H*), 6.48 (dd, *J* = 7.2, 5.0 Hz, 1H, C(6′)*H*), 4.94 (s, 1H, N*H*), 2.70 (t, *J* = 6.4 Hz, 2H, C(4′)H_2), 1.75–1.41 (m, 14H, C(3′)H_2, C(2)H_2, C(3)H_2, C(4)H_2, C(5)H_2, C(6)H_2, C(7)H_2)

^{13}C NMR: (101 MHz $CDCl_3$)

155.6 (*C*(9′)), 146.0 (*C*(7′)), 136.3 (*C*(5′)), 115.7 (*C*(10′)), 112.5 (*C*(6′)), 55.0 (*C*(1)), 41.2 (*C*(2), *C*(7)), 33.6 (*C*(3′)), 30.2 (*C*(3), *C*(6)), 23.3 (*C*(4′)), 22.3 (*C*(4), *C*(5))

IR: (neat)

3258 (w), 2922(w), 2849 (w), 1599 (w), 1578 (w), 1508 (w), 1449 (m), 1354 (w), 1341 (w), 1329 (w), 1289 (w), 1272 (w), 1234 (w), 1195 (w), 1180 (w), 1140 (w), 1100 (w), 1086 (w), 1012 (w), 972 (w), 910 (w), 841 (w), 761 (m), 641 (w).

HRMS: (ESI^{+})

Calcd for $C_{14}H_{20}N_2$: 216.1632, found: 216.1626

Preparation of 3′,4′-dihydro-1′*H*-spiro[cyclopentane-1,2′-[1,8]naphthyridine] (13c)

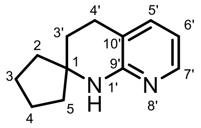

13c

Following the **General Procedure** (section B), 5 mL of reagent feed A [2-fluoro-3-vinylpyridine **17** (185 mg, 1.50 mmol, 1.0 equiv) and 3DPA2FBN (9.6 mg, 15.0 μmol, 1 mol%) in anhydrous DMF], 5 mL of reagent feed B [cyclopentylamine **10c** (128 mg, 1.50 mmol, 1.0 equiv) and NaN_3 (19.5 mg, 300 μmol, 20 mol%) in anhydrous DMF], and 10 mL of reagent feed C [DIPEA (291 mg, 2.25 mmol, 1.5 equiv) in anhydrous DMF] were reacted in flow, setting the high-temperature tube reactor to 180 °C. The steady state mixture (12 mL) was collected and concentrated *in vacuo* on an Asynt spiral evaporator. Purification *via* automated flash column chromatography on SiO_2 gel (12 g) in 40–60 °C petroleum ether (5 CV) then 100:0→0:100 40–60 °C petroleum ether–EtOAc (over 20 CV) then EtOAc (5 CV) gave **13c** as a pale yellow, crystalline solid (123 mg, 73%, 1.63 mmol h^{-1}).

Data for **13c**:

mp: 113–115 °C

^{1}H NMR: (400 MHz, $CDCl_3$)

7.85 (dd, *J* = 5.0, 1.9 Hz, 1H, C(7′)*H*), 7.18 (dd, *J* = 7.2, 1.9 Hz, 1H, C(5′)*H*), 6.48 (dd, *J* = 7.2, 5.0 Hz, 1H, C(6′)*H*), 4.91 (s, 1H, N*H*), 2.74 (t, *J* = 6.4 Hz, 2H, C(4′)H_2), 1.65–1.57 (m, 10H, C(3′)H_2, C(2)H_2, C(3)H_2, C(4)H_2, C(5)H_2).

^{13}C NMR: (101 MHz $CDCl_3$)

155.7 (*C*(9′)), 146.0 (*C*(7′)), 136.2 (*C*(5′)), 115.7 (*C*(10′)), 112.5 (*C*(6′)), 61.3 (*C*(1)), 40.1, (*C*(2), *C*(5)), 32.0 (*C*(3′)), 24.7 (*C*(4′)), 23.6 (*C*(3), *C*(4))

IR: (neat)

3230 (w), 2925 (m), 2864(w), 1599 (m), 1581 (w), 1509 (m), 1446 (s), 1428 (m), 1331 (m), 1308 (m), 1291 (m), 1273 (m), 1249 (m), 1238 (m), 1194 (m), 1184 (m), 1158 (m), 1095 (w), 1070 (w), 1005 (w), 946 (w), 920 (w), 853(w), 760 (s), 666 (m).

HRMS: (ESI^+)

Calcd for $C_{12}H_{16}N_2$: 188.1320, found: 188.1313

Preparation of 3′,4′-dihydro-1′*H*-spiro[cyclobutane-1,2′-[1,8]naphthyridine] (13d)

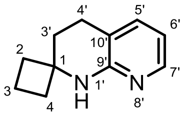

13d

Following the **General Procedure** (section B), 5 mL of reagent feed A [2-fluoro-3-vinylpyridine **17** (185 mg, 1.50 mmol, 1.0 equiv) and 3DPA2FBN (9.6 mg, 15.0 μmol, 1 mol%) in anhydrous DMF], 5 mL of reagent feed B [cyclobutylamine **10d** (107 mg, 1.50 mmol, 1.0 equiv) and NaN_3 (19.5 mg, 300 μmol, 20 mol%) in anhydrous DMF], and 10 mL of reagent feed C [DIPEA (291 mg, 2.25 mmol, 1.5 equiv) in anhydrous DMF] were reacted in flow, setting the high-temperature tube reactor to 180 °C. The steady state mixture (12 mL) was collected and concentrated *in vacuo* on an Asynt spiral evaporator. Purification *via* automated flash column chromatography on SiO_2 gel (12 g) in 40–60 °C petroleum ether (5 CV) then 100:0→0:100 40–60 °C petroleum ether–EtOAc (over 20 CV) then EtOAc (5 CV) gave **13d** as a pale yellow, crystalline solid (123 mg, 79%, 1.78 mmol h^{-1}).

Data for **13d**:

mp: 112–113 °C

^{1}H NMR: (400 MHz, $CDCl_3$)

7.85 (dd, *J* = 5.0, 1.7 Hz, 1H, C(7′)*H*), 7.13 (dd, *J* = 7.2, 1.7 Hz, 1H, C(5′)*H*), 6.45 (dd, *J* = 7.2, 5.0 Hz, 1H, C(6′)*H*), 5.58 (s, 1H, N*H*), 2.67 (t, *J* = 6.4 Hz, 2H, C(4′)H_2), 1.70 (t, *J* = 6.4 Hz, 2H, C(3′)H_2), 2.17–1.95 (m, 4H, C(2)H_2, C(4)H_2), 1.76–1.67 (m, 2H, C(3)H_2)

^{13}C NMR: (101 MHz $CDCl_3$)

155.3 (*C*(9′)), 145.9 (*C*(7′)), 135.7 (*C*(5′)), 115.8 (*C*(10′)), 112.5 (*C*(6′)), 54.7 (*C*(1)), 37.3 (*C*(2), *C*(4)), 30.2 (*C*(3)), 23.6 (*C*(4′)), 12.7 (*C*(3))

IR: (neat)

3235 (w), 2951 (m), 2823 (w), 1600 (m), 1585 (w), 1520 (m), 1451 (m), 1439 (m), 1345 (w), 1327 (m), 1296 (m), 1280 (mw), 1260 (m), 1243 (m), 1210 (w), 1189 (m), 1172 (m), 1117 (m), 1091 (w), 984 (w), 921 (w), 890 (w), 769 (s), 679 (m)

HRMS: (ESI^+)

Calcd for $C_{11}H_{14}N_2$: 174.1157, found: 174.1163

Preparation of 1,3,3′,4′-tetrahydro-1′*H*-spiro[indene-2,2′-[1,8]naphthyridine] (13e)

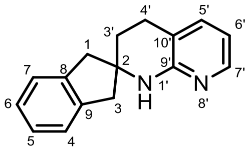

13e

Following the **General Procedure** (section B), 5 mL of reagent feed A [2-fluoro-3-vinylpyridine **17** (185 mg, 1.50 mmol, 1.0 equiv) and 3DPA2FBN (9.6 mg, 15.0 μmol, 1 mol%) in anhydrous DMF], 5 mL of reagent feed B [2-aminoindan **10e** (200 mg, 1.50 mmol, 1.0 equiv) and NaN_3 (19.5 mg, 300 μmol, 20 mol%) in anhydrous DMF], and 10 mL of reagent feed C [DIPEA (291 mg, 2.25 mmol, 1.5 equiv) in anhydrous DMF] were reacted in flow, setting the high-temperature tube reactor to 180 °C. The steady state mixture (10 mL) was collected and concentrated *in vacuo* on an Asynt spiral evaporator. Purification *via* automated flash column chromatography on SiO_2 gel (12 g) in 40–60 °C petroleum ether (5 CV) then 100:0→0:100 40–60 °C petroleum ether–EtOAc (over 20 CV) then EtOAc (5 CV) gave **13e** as a colourless, crystalline solid (133 mg, 75%, 1.68 mmol h^{-1}).

Data for **13e**:

mp: 145–147 °C

^{1}H NMR: (400 MHz, $CDCl_3$)

7.85 (dd, *J* = 5.0, 1.9 Hz, 1H, C(7′)*H*), 7.25–7.38 (m, 5H, C(5′)*H*, C(4)*H*, C(5)*H*, C(6)*H*, C(7)*H*), 6.48 (dd, *J* = 7.2, 5.0 Hz, 1H, C(5′)*H*), 5.13 (s, 1H, N*H*), 3.05 (d, *J* = 16.0 Hz, 2H, C(4′)H_2), 3.04 (d, *J* = 1.9 Hz, 4H, C(1)H_2, C(3)H_2), 2.84 (t, *J* = 6.6 Hz, 2H, C(4′)H_2), 1.97 (t, *J* = 6.6 Hz, 2H, C(3′)H_2)

^{13}C NMR: (101 MHz $CDCl_3$)

155.6 (*C*(9′)), 146.2 (*C*(7′)), 140.1 (*C*(8), *C*(9)), 136.4(*C*(5′)), 126.9 (*C*(4), *C*(7)), 125.1 ((*C*(5), *C*(6)), 115.5 (*C*(10′)), 113.1 (*C*(6′)), 62.1 (*C*(2)), 47.0 (*C*(1), *C*(3)), 31.4 (*C*(3′)), 24.4 (*C*(4′))

IR: (neat)

3261 (m), 3065 (w), 2929 (m), 2839(w), 1596 (m), 1575 (w), 1504 (m), 1481 (s), 1443 (m), 1432 (m), 1420 (m), 1334 (m), 1302 (m), 1283 (m), 1256 (m), 1233 (w), 1209 (m), 1183 (m), 1153 (w), 1158 (m), 1122 (w), 1097 (m), 1073 (w), 1055 (m), 1025 (w), 1013 (m), 964 (m), 950 (w), 919 (m), 897 (m), 852 (w), 803 (w), 765 (s), 739 (s), 699 (m), 675 (m), 616 (m), 600 (m), 586 (m), 567 (m), 559 (m)

HRMS: (ESI^+)

Calcd for $C_{16}H_{16}N_2$: 236.1313, found: 236.1320

Preparation of 3′,4′-dihydro-1′*H*-spiro[cyclohexane-1,2′-[1,8]naphthyridin]-4-ol (13f)

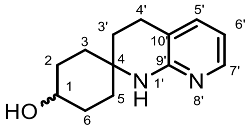

13f

Following the **General Procedure** (section B), 5 mL of reagent feed A [2-fluoro-3-vinylpyridine **17** (185 mg, 1.50 mmol, 1.0 equiv) and 3DPA2FBN (9.6 mg, 15.0 μmol, 1 mol%) in anhydrous DMF], 5 mL of reagent feed B [4-aminocyclohexan-1-ol **10f** (173 mg, 1.50 mmol, 1.0 equiv) and NaN_3 (19.5 mg, 300 μmol, 20 mol%) in anhydrous DMF], and 10 mL of reagent feed C [DIPEA (291 mg, 2.25 mmol, 1.5 equiv) in anhydrous DMF] were reacted in flow, setting the high-temperature tube reactor to 180 °C. The steady state mixture (12 mL) was collected and concentrated *in vacuo* on an Asynt spiral evaporator. Purification *via* automated flash column chromatography on SiO_2 gel (12 g) in CH_2Cl_2 (5 CV) then 100:0:0→95:4.5:0.5 CH_2Cl_2–MeOH–aq. NH_4OH (over 20 CV) then 95:4.5:0.5 CH_2Cl_2–MeOH–aq. NH_4OH (5 CV) gave **13f** as a colourless, amorphous solid (130 mg, 67%, 1:1 dr, 1.50 mmol h^{-1}).

Data for **13f** (diastereomeric mixture):

IR: (neat)

3545 (w), 3390 (w), 3247 (w), 2930 (2), 2854 (w), 1600 (m), 1585 (m), 1520 (w), 1500 (m), 1453 (m), 1369 (w), 1337 (w), 1318 (w), 1298 (w), 1282 (m), 1240 (w), 1192 (w), 1165 (m), 1121 (w), 1104 (w), 1084 (w), 1051 (m), 1028 (w), 1007 (w), 955 (w), 929 (w), 906 (w), 882(w), 758 (m), 652 (m), 608 (m), 590 (w), 564 (w)

HRMS: (ESI^+)

Calcd for $C_{13}H_{18}N_2O$: 218.1431, found: 218.1419

Data for **13f** (diastereomer 1):

^{1}H NMR: (400 MHz, $CDCl_3$)

7.83 (dd, *J* = 5.0, 1.9 Hz, 1H, C(7′)*H*), 7.17 (dd, 1H, *J* = 7.2, 1.9 Hz, C(5′)*H*), 6.47 (dd, *J* = 7.2, 5.0 Hz, 1H, C(6′)*H*), 5.46 (s, 1H, N*H*), 3.71 (tt, *J* = 7.6, 3.3 Hz, 1H, C(1)*H*), 3.15 (br s, 1H, O*H*), 2.70 (t, *J* = 6.7 Hz, 2H, C(4′)H_2), 1.93–1.84 (m, 2H, C(2)H_A, C(6)H_A), 1.83–1.72 (m, 2H, C(3)H_A, C(5)H_A), 1.70–1.56 (m, 4H, C(2)H_B, C(6)H_B, C(3′)H_2), 1.46–1.36 (m, 2H, C(3)H_B, C(5)H_B)

<u>^{13}C NMR:</u> (101 MHz $CDCl_3$)

155.1 (*C*(9′)), 145.5 (*C*(7′)), 136.3 (*C*(5′)), 115.5 (*C*(10′), 112.5 (*C*(6′)), 69.4 (*C*(1′)), 50.1 (*C*(4)), 34.9 (*C*(3), *C*(5)), 32.9 (*C*(3′)), 30.4 (*C*(2), *C*(6)), 23.1 (*C*(4′))

<u>Data for **13f** (diastereomer 2):</u>

<u>^{1}H NMR:</u> (400 MHz, $CDCl_3$)

7.83 (dd, *J* = 5.0, 1.9 Hz, 1H, C(7′)*H*), 7.17 (dd, 1H, *J* = 7.2, 1.9 Hz, C(5′)*H*), 6.48 (dd, *J* = 7.2, 5.0 Hz, 1H, C(6′)*H*), 5.01 (s, 1H, N*H*), 3.87 (tt, *J* = 7.6, 3.3 Hz, 1H, C(1)*H*), 2.70 (t, *J* = 6.7 Hz, 2H, C(4′)H_2), 2.51 (br s, 1H, O*H*), 1.90–1.74 (m, 6H, C(2)H_A, C(3)H_A, C(5)H_A, C(6)H_A, C(3′)H_2), 1.65–1.53 (m, 2H, C(2)H_B, C(6)H_B), 1.52–1.40 (m, 2H, C(3)H_B, C(5)H_B)

<u>^{13}C NMR:</u> (101 MHz $CDCl_3$)

155.4 (*C*(9′)), 145.6 (*C*(7′)), 136.5 (*C*(5′)), 115.6 (*C*(10′), 112.6 (*C*(6′)), 68.0 (*C*(1′)), 51.1 (*C*(4)), 34.0 (*C*(3), *C*(5)), 30.2 (*C*(3′)), 30.1, (*C*(2), *C*(6)), 22.8 (*C*(4′))

Preparation of 2,3,3′,4′,5,6-hexahydro-1′*H*-spiro[pyran-4,2′-[1,8]naphthyridine] (13g)

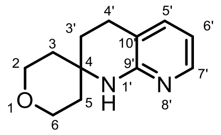

13g

Following the **General Procedure** (section B), 5 mL of reagent feed A [2-fluoro-3-vinylpyridine **17** (185 mg, 1.50 mmol, 1.0 equiv) and 3DPA2FBN (9.6 mg, 15.0 μmol, 1 mol%) in anhydrous DMF], 5 mL of reagent feed B [tetrahydro-2*H*-pyran-4-amine **10g** (152 mg, 1.50 mmol, 1.0 equiv) and NaN_3 (19.5 mg, 300 μmol, 20 mol%) in anhydrous DMF], and 10 mL of reagent feed C [DIPEA (291 mg, 2.25 mmol, 1.5 equiv) in anhydrous DMF] were reacted in flow, setting the high-temperature tube reactor to 200 °C. The steady state mixture (12 mL) was collected and concentrated *in vacuo* on an Asynt spiral evaporator. Purification *via* automated flash column chromatography on SiO_2 gel (12 g) in 40–60 °C petroleum ether (5 CV) then 100:0→0:100 40–60 °C petroleum ether–EtOAc (over 20 CV) then EtOAc (5 CV) gave **13g** as a colourless, crystalline solid (155 mg, 85%, 1.93 mmol h^{-1}).

<u>Data for **13g**:</u>

<u>mp:</u> 137–138 °C

<u>^{1}H NMR:</u> (400 MHz, $CDCl_3$)

7.89 (dd, *J* = 5.0, 1.7 Hz, 1H, C(7′)*H*), 7.20 (dd, *J* = 7.2, 1.7 Hz, 1H, C(5′)*H*), 6.53 (dd, *J* = 7.2, 5.0 Hz, 1H, C(6′)*H*), 5.19 (s, 1H, N*H*), 3.85–3.69 (m, 4H, C(2)H_2, C(6)H_2), 2.74 (t, *J* = 7.0 Hz, 2H, C(4′)H_2), 1.82–1.60 (m, 6H, C(3′)H_2, C(3)H_2, C(5)H_2)

<u>^{13}C NMR:</u> (101 MHz $CDCl_3$)

155.1 (*C*(9′)), 146.0 (*C*(7′)), 136.6 (*C*(5′)), 115.5 (*C*(10′)), 113.1 (*C*(6′)), 63.8 (*C*(2), *C*(6)), 49.1 (*C*(4)), 37.9 (*C*(3), *C*(5)), 32.4 (*C*(3′)), 22.5 (*C*(4′))

<u>IR:</u> (neat)

3232 (w), 3098 (w), 3022 (w), 2953 (w), 2934 (m), 2848 (m), 1603 (m), 1585 (m), 1521 (m), 1468 (w), 1452 (m), 1438 (m), 1382 (m), 1352 (m), 1341 (m), 1280 (m), 1244 (m), 1218 (m), 1192 (w), 1170 (m), 1134 (m), 1105 (m), 1096 (s), 1081 (m), 1036 (m), 1024 (w), 1011 (m), 1002 (m), 990 (m), 961 (w), 935 (w), 908 (m), 881 (w), 837 (m), 805 (w), 763 (s), 716 (m), 668 (m)

<u>HRMS:</u> (ESI^+)

Calcd for $C_{12}H_{16}N_2O$: 204.1263, found: 204.1268

Preparation of 2,3,3′,4′,5,6-hexahydro-1′*H*-spiro[thiopyran-4,2′-[1,8]naphthyridine] (13h)

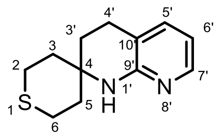

13h

Following the **General Procedure** (section B), 5 mL of reagent feed A [2-fluoro-3-vinylpyridine **17** (185 mg, 1.50 mmol, 1.0 equiv) and 3DPA2FBN (9.6 mg, 15.0 μmol, 1 mol%) in anhydrous DMF], 5 mL of reagent feed B [tetrahydro-2*H*-thiopyran-4-amine **10h** (176 mg, 1.50 mmol, 1.0 equiv) and NaN_3 (19.5 mg, 300 μmol, 20 mol%) in anhydrous DMF], and 10 mL of reagent feed C [DIPEA (291 mg, 2.25 mmol, 1.5 equiv) in anhydrous DMF] were reacted in flow, setting the high-temperature tube reactor to 200 °C. The steady state mixture (12 mL) was collected and concentrated *in vacuo* on an Asynt spiral evaporator. Purification *via* automated flash column chromatography on SiO_2 gel (12 g) in 40–60 °C petroleum ether (5 CV) then 100:0→0:100 40–60 °C petroleum ether–EtOAc (over 20 CV) then EtOAc (5 CV) gave **13h** as a colourless, crystalline solid (162 mg, 83%, 1.86 mmol h^{-1}).

<u>Data for **13h**:</u>

<u>mp:</u> 141–143 °C

<u>^{1}H NMR:</u> (400 MHz, $CDCl_3$)

7.89 (dd, J = 5.0, 1.7 Hz, 1H, C(7′)H), 7.19 (dd, J = 7.2, 1.7 Hz, 1H, C(5′)H), 6.52 (dd, J = 7.2, 5.0 Hz, 1H, C(6′)H), 4.98 (s, 1H, NH), 2.85–2.75 (m, 2H, C(2)H_A, C(6)H_A), 2.72 (t, J = 6.7 Hz, 2H, C(4′)H_2), 2.68–2.57 (m, 2H, C(2)H_B, C(6)H_B), 1.97–1.80 (m, 4H, C(3)H_2, C(5)H_2), 1.74 (t, J = 6.7 Hz, 2H, C(3′)H_2)

<u>^{13}C NMR:</u> (101 MHz $CDCl_3$)

155.0 (C(9′)), 146.0 (C(7′)), 136.7 (C(5′)), 115.4 (C(10′)), 113.1 (C(6′)), 50.1 (C(4)), 38.6 (C(3), C(5)), 32.0 (C(3′)), 24.0 (C(2), C(6)), 22.3 (C(4′))

<u>IR:</u> (neat)

3234 (m), 3096 (w), 3019 (w), 2928 (w), 2900 (m), 2836 (m), 1598 (m), 1582 (m), 1517 (m), 1451 (m), 1429 (m), 1354 (m), 1339 (m), 1324 (m), 1307 (m), 1267 (m), 1242 (m), 1200 (m), 1190 (w), 1171 (w), 1114 (m), 1099 (m), 1058 (m), 1016 (m), 998 (w), 973 (w), 956 (w), 941 (m), 935 (m), 917 (m), 888 (w), 867 (w), 775 (m), 762 (s), 717 (m), 701 (m), 677 (m), 660 (m), 624 (m), 570 (m)

<u>HRMS:</u> (ESI^+)

Calcd for $C_{12}H_{16}N_2S$: 220.1034, found: 220.1034

Preparation of *tert*-butyl 3′,4′-dihydro-1′*H*-spiro[piperidine-4,2′-[1,8]naphthyridine]-1-carboxylate (13i)

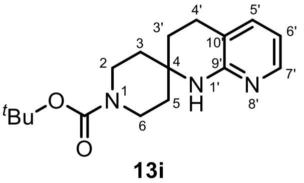

13i

Following the **General Procedure** (section B), 5 mL of reagent feed A [2-fluoro-3-vinylpyridine **17** (185 mg, 1.50 mmol, 1.0 equiv) and 3DPA2FBN (9.6 mg, 15.0 µmol, 1 mol%) in anhydrous DMF], 5 mL of reagent feed B [*tert*-butyl 4-aminopiperidine-1-carboxylate **10i** (300 mg, 1.50 mmol, 1.0 equiv) and NaN_3 (19.5 mg, 300 µmol, 20 mol%) in anhydrous DMF], and 10 mL of reagent feed C [DIPEA (291 mg, 2.25 mmol, 1.5 equiv) in anhydrous DMF] were reacted in flow, setting the high-temperature tube reactor to 200 °C. The steady state mixture (12 mL) was collected and concentrated *in vacuo* on an Asynt spiral evaporator. Purification *via* automated flash column chromatography on SiO_2 gel (12 g) in 40–60 °C petroleum ether (5 CV) then 100:0→0:100 40–60 °C petroleum ether–EtOAc (over 20 CV) then EtOAc (5 CV) gave **13i** as a colourless, crystalline solid (241 mg, 89%, 1.99 mmol h^{-1}).

Data for **13i**:

mp: 121–122 °C

^{1}H NMR: (400 MHz, $CDCl_3$)

7.88 (dd, *J* = 5.1, 1.7 Hz, 1H, C(7′)*H*), 7.19 (dd, 1H, *J* = 7.2, 1.7 Hz, C(5′)*H*), 6.52 (dd, *J* = 7.2, 5.1 Hz, 1H, C(6′)*H*), 5.05 (s, 1H, N*H*), 3.73–3.62 (m, 2H, C(2)H_A, C(6)H_A), 3.36–3.25 (m, 2H, C(2)H_B, C(6)H_B), 2.73 (t, *J* = 6.4 Hz, 2H, C(4′)H_2), 1.75 (t, *J* = 6.4 Hz, 2H, C(3′)H_2), 1.67–1.58 (m, 4H, C(3)H_2, C(5)H_2), 1.46 (s, 9H, OC(CH_3)$_3$)

^{13}C NMR: (101 MHz $CDCl_3$)

155.0 (*C*=O), 154.8 (*C*(9′)), 145.9 (*C*(7′)), 136.5 (*C*(5′)), 115.3 (*C*(10′)), 113.0 (*C*(6′)), 79.7 (O*C*(CH_3)$_3$), 49.9 (*C*(4)), 39.5 (br s, *C*(2), *C*(6)), 36.9 (*C*(3), *C*(5)), 31.8 (*C*(3′)), 28.5 (OC(*C*H_3)$_3$), 22.6 (*C*(4′))

IR: (neat)

3239 (w), 3097 (w), 2997 (w), 2931 (w), 1687 (s), 1602 (m), 1582 (m), 1523 (m), 1473 (w), 1455 (m), 1426 (m), 1385 (w), 1361 (m), 1324 (w), 1280 (m), 1246 (m), 1232 (m), 1162 (m), 1123 (m), 1103 (m), 1070 (m), 1027 (w), 991 (m), 981 (w), 950 (w), 934 (w), 915 (w), 905 (w), 879 (w), 867 (w), 825 (w), 761 (s), 717 (w), 682 (w)

HRMS: (ESI^+)

Calcd for $C_{17}H_{25}N_3O_2$: 303.1947, found: 303.1954

Preparation of *tert*-butyl 3′,4′-dihydro-1′*H*-spiro[azetidine-3,2′-[1,8]naphthyridine]-1-carboxylate (13j)

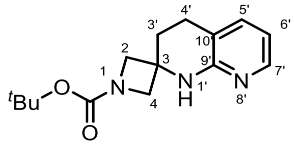

13j

Following the **General Procedure** (section B), 5 mL of reagent feed A [2-fluoro-3-vinylpyridine **17** (185 mg, 1.50 mmol, 1.0 equiv) and 3DPA2FBN (9.6 mg, 15.0 μmol, 1 mol%) in anhydrous DMF], 5 mL of reagent feed B [*tert*-butyl 3-aminoazetidine-1-carboxylate **10j** (775 mg, 4.50 mmol, 3.0 equiv) and NaN_3 (19.5 mg, 300 μmol, 20 mol%) in anhydrous DMF], and 10 mL of reagent feed C [DIPEA (291 mg, 2.25 mmol, 1.5 equiv) in anhydrous DMF] were reacted in flow, setting the high-temperature tube reactor to 200 °C. The steady state mixture (12 mL) was collected and concentrated *in vacuo* on an Asynt spiral evaporator. Purification *via* automated flash column chromatography on SiO_2 gel (12 g)

in 40–60 °C petroleum ether (5 CV) then 100:0→0:100 40–60 °C petroleum ether–EtOAc (over 20 CV) then EtOAc (5 CV) gave **13j** as a colourless, crystalline solid (194 mg, 80%, 1.79 mmol h^{-1}).

Data for **13j**:

mp: 148–150 °C

^{1}H NMR: (400 MHz, $CDCl_3$)

7.89 (dd, *J* = 5.0, 1.8 Hz, 1H, C(7′)*H*), 7.19 (dd, 1H, *J* = 7.3, 1.8 Hz, C(5′)*H*), 6.55 (dd, *J* = 7.3, 5.1 Hz, 1H, C(6′)*H*), 6.08 (s, 1H, N*H*), 3.92 (d, *J* = 8.9 Hz, 2H, C(2)H_A, C(4)H_A), 3.86 (d, *J* = 8.9 Hz, 2H, C(2)H_B, C(4)H_B), 2.73 (t, *J* = 6.4 Hz, 2H, C(4′)H_2), 2.04 (t, *J* = 6.4 Hz, 2H, C(3′)H_2), 1.45 (s, 9H, OC(CH_3)$_3$).

^{13}C NMR: (101 MHz $CDCl_3$)

156.6 (*C*=O), 154.7 (*C*(9′)), 146.1 (*C*(7′)), 136.3 (*C*(5′)), 115.5 (*C*(10′)), 113.8 (*C*(6′)), 80.0 (O*C*(CH_3)$_3$), 63.3 (br s, *C*(2), *C*(4)), 50.8 (*C*(3)), 30.4 (*C*(3′)), 28.5 (OC(*C*H_3)$_3$), 23.3 (*C*(4′))

IR: (neat)

3217 (w), 3101 (w), 3012 (w), 2973 (w), 2951 (w), 2937 (w), 2877 (w), 1698 (m), 1603 (w), 1531 (w), 1474 (w), 1455 (m), 1443 (m), 1429 (w), 1404 (m), 1362 (m), 1316 (w), 1284 (w), 1248 (w), 1217 (w), 1180 (w), 1140 (m), 1106 (m), 1081(m), 958 (w), 934 (w), 917 (w), 885 (w), 860 (w), 765 (s), 702 (w), 668 (w), 634 (w), 591 (w), 567 (w)

HRMS: (ESI^+)

Calcd for $C_{15}H_{21}N_3O_2$: 275.1634, found: 275.1641

Preparation of 3′,4′-dihydro-1′*H*-spiro[oxetane-3,2′-[1,8]naphthyridine] (13k)

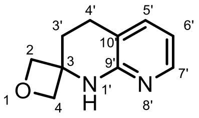

13k

Following the **General Procedure** (section B), 5 mL of reagent feed A [2-fluoro-3-vinylpyridine **17** (185 mg, 1.50 mmol, 1.0 equiv) and 3DPA2FBN (9.6 mg, 15.0 μmol, 1 mol%) in anhydrous DMF], 5 mL of reagent feed B [oxetan-3-amine **10k** (329 mg, 4.50 mmol, 3.0 equiv) and NaN_3 (19.5 mg, 300 μmol, 20 mol%) in anhydrous DMF], and 10 mL of reagent feed C [DIPEA (291 mg, 2.25 mmol, 1.5 equiv) in anhydrous DMF] were reacted in flow, setting the high-temperature tube reactor to 200 °C. The steady state mixture (12 mL) was collected and concentrated *in vacuo* on an Asynt spiral evaporator. Purification *via* automated flash column chromatography on SiO_2 gel (12 g) in 40–60 °C petroleum

ether (5 CV) then 100:0→0:100 40–60 °C petroleum ether–EtOAc (over 20 CV) then EtOAc (5 CV) gave **13k** as a colourless, crystalline solid (96.0 mg, 61%, 1.38 mmol h^{-1}).

Data for **13k**:

mp: 160–161°C

^{1}H NMR: (400 MHz, $CDCl_3$)

7.88 (dd, *J* = 5.0, 1.7 Hz, 1H, C(7′)*H*), 7.19 (dd, 1H, *J* = 7.2, 1.7 Hz, C(5′)*H*), 6.52 (dd, *J* = 7.2, 5.1 Hz, 1H, C(6′)*H*), 6.52 (s, 1H, N*H*), 3.73–3.62 (m, 2H, C(2)H_A, C(4)H_A), 3.36–3.25 (m, 2H, C(2)H_B, C(4)H_B), 2.73 (t, *J* = 6.7 Hz, 2H, C(4′)H_2), 2.19 (t, *J* = 6.7 Hz, 2H, C(3′)H_2)

^{13}C NMR: (101 MHz $CDCl_3$)

154.7 (*C*(9′)), 146.3 (*C*(7′)), 136.1 (*C*(5′)), 115.6, (*C*(10′)), 113.7 (*C*(6′)), 85.0 (*C*(2), *C*(4)), 55.5 (*C*(3)), 30.0 (*C*(3′)), 23.3 (*C*(4′))

IR: (neat)

3215 (m), 3171 (w), 3100 (w), 3016 (w), 2936 (m), 2858 (m), 1603 (s), 1591 (m), 1526 (m), 1478 (m), 1453 (s), 1443 (s), 1333 (m), 1290 (m), 1257 (m), 1220 (w), 1190 (m), 1132 (m), 1080 (w), 1010 (w), 964 (s), 923 (m), 891 (m), 813 (m), 779 (m), 763 (s), 714 (m), 622 (m), 601 (m), 581 (m), 560 (m)

HRMS: (ESI$^+$)

Calcd for $C_{10}H_{12}N_2O$: 176.0950, found: 176.0955

Preparation of 2,2-dimethyl-1,2,3,4-tetrahydro-1,8-naphthyridine (13l)

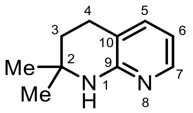

13l

Following the **General Procedure** (section B), 5 mL of reagent feed A [2-fluoro-3-vinylpyridine **17** (185 mg, 1.50 mmol, 1.0 equiv) and 3DPA2FBN (9.6 mg, 15.0 μmol, 1 mol%) in anhydrous DMF], 5 mL of reagent feed B [isopropylamine **10l** (266 mg, 4.50 mmol, 3.0 equiv) and NaN_3 (19.5 mg, 300 μmol, 20 mol%) in anhydrous DMF], and 10 mL of reagent feed C [DIPEA (291 mg, 2.25 mmol, 1.5 equiv) in anhydrous DMF] were reacted in flow, setting the high-temperature tube reactor to 180 °C. The steady state mixture (5.5 mL) was collected and concentrated *in vacuo* on an Asynt spiral evaporator. Purification *via* automated flash column chromatography on SiO_2 gel (12 g) in 40–60 °C

petroleum ether (5 CV) then 100:0→0:100 40–60 °C petroleum ether–EtOAc (over 20 CV) then EtOAc (5 CV) gave **13l** as an off-white, crystalline solid (49.1 mg, 75%, 1.79 mmol h^{-1}).

Data for **13l**:

mp: 90–92 °C

^{1}H NMR: (400 MHz, $CDCl_3$)

7.86 (dd, *J* = 5.0, 1.7 Hz, 1H, C(7)*H*), 7.17 (dd, *J* = 7.2, 1.7 Hz, 1H, C(5)*H*), 6.48 (dd, *J* = 7.2, 5.0 Hz, 1H, C(6)*H*), 4.78 (s, 1H, N*H*), 2.73 (t, *J* = 6.7 Hz, 2H, C(4)H_2), 1.67 (t, *J* = 6.7 Hz, 2H, C(3)H_2), 1.24 (s, 5H, C(CH_3)$_2$)

^{13}C NMR: (101 MHz $CDCl_3$)

155.8 (*C*(9)), 146.2 (*C*(7)), 136.2 (*C*(5)), 115.0 (*C*(10)), 112.5 (*C*(6)), 49.7 (*C*(2)), 33.9 (*C*(3)), 29.6 (C(*C*H_3)$_2$), 23.7 (*C*(4))

IR: (neat)

3241 (m), 3091 (w), 3018 (w), 2970 (w), 2922 (m), 1599 (m), 1579 (m), 1443 (m), 1371 (m), 1364 (m), 1333 (m), 1280 (m), 1251 (m), 1232 (w), 1182 (w), 1154 (s), 1129 (m), 1098 (m), 1006 (m), 1010 (m), 757 (s), 728 (m), 639 (m), 589 (m)

HRMS: (ESI^{+})

Calcd for $C_{10}H_{14}N_2$: 162.1157, found: 162.1160

Preparation of 2-methyl-1,2,3,4-tetrahydro-1,8-naphthyridine (13m)

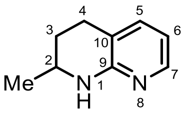

13m

Following the **General Procedure** (section B), 5 mL of reagent feed A [2-fluoro-3-vinylpyridine **17** (185 mg, 1.50 mmol, 1.0 equiv) and 3DPA2FBN (9.6 mg, 15.0 µmol, 1 mol%) in anhydrous DMF], 5 mL of reagent feed B [ethylamine **10m** (2 M in THF, 266 mg, 4.50 mmol, 3.0 equiv) and NaN_3 (19.5 mg, 300 µmol, 20 mol%) in anhydrous DMF], and 10 mL of reagent feed C [DIPEA (291 mg, 2.25 mmol, 1.5 equiv) in anhydrous DMF] were reacted in flow, setting the high-temperature tube reactor to 180 °C. The steady state mixture (9.2 mL) was collected and concentrated *in vacuo* on an Asynt spiral evaporator. Purification *via* automated flash column chromatography on SiO_2 gel (12 g) in 40–60 °C petroleum ether (5 CV) then 100:0→0:100 40–60 °C petroleum ether–EtOAc (over 20 CV) then EtOAc (5 CV) gave **13m** as a colourless, crystalline solid (36.6 mg, 36%, 0.86 mmol h^{-1}). *The obtained analytical data were in full agreement with the literature.*[9]

Data for **13m**

mp: 59–61 °C (lit.[9]: 57–59 °C)

^{1}H NMR: (400 MHz, $CDCl_3$)

7.85 (dd, *J* = 5.0, 1.7 Hz, 1H, C(7)*H*), 7.14 (dd, *J* = 7.2, 1.7 Hz, 1H, C(5)*H*), 6.47 (dd, *J* = 7.2, 5.0 Hz, 1H, C(6)*H*), 4.78 (s, 1H, N*H*), 3.55 (dqdd, *J* = 9.6, 6.4, 3.2, 1.2 Hz, 1H, C(2)*H*), 2.84–2.64 (m, 2H, C(4)H_2), 1.93 (dddd, 1H, *J* = 12.9, 5.3, 4.2, 3.2 Hz C(3)H_A), 1.55 (dddd, 1H, *J* = 12.9, 11.0, 9.6, 5.3 Hz, C(3)H_B), 1.24 (d, *J* = 6.4 Hz, 2H, CH_3)

^{13}C NMR: (101 MHz $CDCl_3$)

156.3 (*C*(9)), 146.0 (*C*(7)), 136.1 (*C*(5)), 115.9 (*C*(10)), 112.8 (*C*(6)), 47.2 (*C*(2)), 29.3 (*C*(3)), 26.0 (*C*(4)), 22.6 (*C*H_3)

IR: (neat)

3241 (m), 3099 (w), 3019 (w), 2960 (w), 2924 (m), 2849 (w), 1600 (m), 1582 (m), 1519 (m), 1440 (m), 1429 (m), 1371 (m), 1346 (m), 1328 (m), 1303 (m), 1278 (m), 1250 (m), 1219 (w), 1189 (m), 1161 (w), 1112 (m), 1092 (m), 1061 (m), 1010 (m), 970 (w), 957 (w), 916 (m), 872 (w), 760 (s), 735 (m), 668 (m), 632 (m), 587 (m), 562 (m)

HRMS: (ESI^+)

Calcd for $C_9H_{12}N_2$: 148.1000, found: 148.1005

Preparation of (*RS*)-2-benzyl-1,2,3,4-tetrahydro-1,8-naphthyridine (13n)

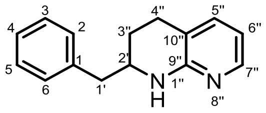

13n

Following the **General Procedure** (section B), 25 mL of reagent feed A [2-fluoro-3-vinylpyridine **17** (923.5 mg, 7.50 mmol, 1.0 equiv), 3DPA2FBN (48.2 mg, 75.0 µmol, 1 mol%) in anhydrous DMF], 25 mL of reagent feed B [2-phenylethylamine **10n** (2.73 g, 22.5 mmol, 3.0 equiv) and NaN_3 (998 mg, 1.50 mmol, 20 mol%) in anhydrous DMF], and 50 mL of reagent feed C [DIPEA (1.46 g, 11.25 mmol, 1.5 equiv) in anhydrous DMF] were reacted in flow, setting the high-temperature tube reactor to 180 °C. The steady state mixture (94 mL) was collected and concentrated *in vacuo*. Purification *via* automated flash column chromatography on SiO_2 gel (24 g) in 40–60 °C petroleum ether (5 CV) then 100:0→0:100 40–60 °C petroleum ether–EtOAc (over 20 CV) then EtOAc (5 CV) gave **13n** as a colourless, crystalline solid (1.21 g, 77%, 1.73 mmol h^{-1}).

Data for **13n:**

mp: 99–100 °C

^{1}H NMR: (400 MHz, $CDCl_3$)

7.85 (dd, *J* = 5.0, 1.8 Hz, 1H, C(7″)*H*), 7.40–7.31 (m, 2H, C(3)*H*, C(5)*H*), 7.30–7.22 (m, 3H, C(2)*H*, C(4)*H*, C(6)*H*), 7.17 (dd, *J* = 7.2, 1.8 Hz, 1H, C(5″)*H*), 6.50 (dd, *J* = 7.2, 5.0 Hz, 1H, C(6″)*H*), 4.81 (s, 1H, N*H*), 3.73–3.62 (m, 1H, C(2′)*H*), 2.92 (dd, *J* = 13.3, 5.2 Hz, 1H, C(1′)H_A), 2.80–2.70 (m, 3H, C(4″)H_2, C(1′)H_B), 2.07–1.98 (m, 1H, C(3″)H_A), 1.77–1.64 (m, 1H, C(3″)H_B)

^{13}C NMR: (101 MHz $CDCl_3$)

156.1 (*C*(9″)), 146.1 (*C*(7″)), 138.0 (*C*(1)), 136.2 (*C*(5″)), 129.4 (*C*(2), *C*(6)), 128.9 (*C*(3), *C*(5)), 126.8 (*C*(4)), 116.1 (*C*(10″)), 113.1 (*C*(6″)), 52.9 (*C*(2′)), 43.1 (*C*(1′)), 27.8 (*C*(3″)), 25.8 (*C*(4″))

IR: (neat)

3220 (m), 3092 (w), 3026 (w), 2945 (w), 2926 (w), 2849 (w), 1603 (m), 1583 (m), 1523 (m), 1454 (m), 1439 (m), 1351 (m), 1318 (m), 1302 (m), 1282 (m), 1254 (m), 1221 (w), 1191 (m), 1134 (w), 1107 (m), 1080 (m), 1062 (w), 1053 (w), 1034 (m), 951 (w), 929 (w), 914 (w), 879 (w), 841 (w), 821 (w), 773 (m), 762 (m), 745 (s), 695 (s), 641 (m), 613 (m), 577 (m), 568 (m), 560 (m)

HRMS: (ESI$^+$)

Calcd for $C_{15}H_{16}N_2$: 224.1313, found: 224.1319

Preparation of *tert*-butyl ((1,2,3,4-tetrahydro-1,8-naphthyridin-2-yl)methyl)carbamate (13o)

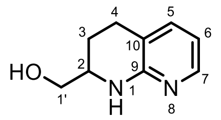

13o

Following the **General Procedure** (section B), 5 mL of reagent feed A [2-fluoro-3-vinylpyridine **17** (185 mg, 1.50 mmol, 1.0 equiv) and 3DPA2FBN (9.6 mg, 15.0 µmol, 1 mol%) in anhydrous DMF], 5 mL of reagent feed B [ethanolamine **10o** (212 mg, 4.50 mmol, 3.0 equiv) and NaN_3 (19.5 mg, 300 µmol, 20 mol%) in anhydrous DMF], and 10 mL of reagent feed C [DIPEA (291 mg, 2.25 mmol, 1.5 equiv) in anhydrous DMF] were reacted in flow, setting the high-temperature tube reactor to 180 °C. The steady state mixture (12 mL) was collected and concentrated *in vacuo* on an Asynt spiral evaporator. Purification *via* automated flash column chromatography on SiO_2 gel (12 g) in CH_2Cl_2 (5 CV) then

100:0:0→95:4.5:0.5 CH_2Cl_2–MeOH–aq. NH_4OH (over 20 CV) then 95:4.5:0.5 CH_2Cl_2–MeOH–aq. NH_4OH (5 CV) gave **13o** as a yellow oil (125 mg, 85%, 2.02 mmol h^{-1}).

Data for **13o**:

^{1}H NMR: (400 MHz, $CDCl_3$)

7.80 (dd, *J* = 5.0, 1.7 Hz, 1H, C(7)*H*), 7.15 (dd, *J* = 7.2, 1.7 Hz, 1H, C(5)*H*), 6.48 (dd, *J* = 7.2, 5.0 Hz, 1H, C(6)*H*), 5.59 (s, 1H, N*H*), 3.77 (dd, *J* = 10.2, 3.3 Hz, 1H, C(1′)H_A), 3.64–3.56 (m, 1H, C(2)*H*), 3.51 (dd, *J* = 10.2, 8.7 Hz, 1H, C(1′)H_B), 2.84–2.64 (m, 2H, C(4)H_2), 1.94–1.83 (m, 1H, C(3)H_A), 1.65–1.53 (m, 1H, C(3)H_B)

^{13}C NMR: (101 MHz $CDCl_3$)

156.2 (*C*(9)) , 145.5 (*C*(7)), 136.4 (*C*(5)), 116.7 (*C*(10)), 112.7 (*C*(6)), 66.0 ((*C*1′)), 52.8 (*C*(2)), 25.7 (*C*(4)), 23.7 (*C*(3))

IR: (neat)

3306 (br, w), 2924 (m), 2845 (w), 1599 (m), 1498 (s), 1455 (s), 1353 (m), 1332 (m), 1293 (m), 1191 (m), 1113 (w), 1065 (m), 995 (w), 915 (w), 762 (m), 613 (m)

HRMS: (ESI^+)

Calcd for $C_9H_{12}N_2O$: 164.0950, found: 164.0955

Preparation of *tert*-butyl ((1,2,3,4-tetrahydro-1,8-naphthyridin-2-yl)methyl)carbamate (13p)

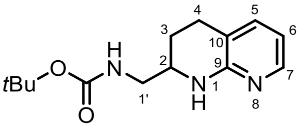

13p

Following the **General Procedure** (section B), 5 mL of reagent feed A [2-fluoro-3-vinylpyridine **17** (185 mg, 1.50 mmol, 1.0 equiv) and 3DPA2FBN (9.6 mg, 15.0 μmol, 1 mol%) in anhydrous DMF], 5 mL of reagent feed B [*tert*-butyl (aminoethyl)carbamate **10p** (658 mg, 4.50 mmol, 3.0 equiv) and NaN_3 (19.5 mg, 300 μmol, 20 mol%) in anhydrous DMF], and 10 mL of reagent feed C [DIPEA (291 mg, 2.25 mmol, 1.5 equiv) in anhydrous DMF] were reacted in flow, setting the high-temperature tube reactor to 180 °C. The steady state mixture (12 mL) was collected and concentrated *in vacuo* on an Asynt spiral evaporator. Purification *via* automated flash column chromatography on SiO_2 gel (12 g) in 40–60 °C petroleum ether (5 CV) then 100:0→0:100 40–60 °C petroleum ether–EtOAc (over 20 CV) then EtOAc (5 CV) gave **13p** as a colourless, crystalline solid (125 mg, 53%, 1.26 mmol h^{-1}).

Data for **13p**:

mp: 122–124 °C

^{1}H NMR: (400 MHz, $CDCl_3$)

7.87 (dd, *J* = 5.0, 1.7 Hz, 1H, C(7)*H*), 7.16 (dd, *J* = 7.2, 1.7 Hz, 1H, C(5)*H*), 6.51 (dd, *J* = 7.2, 5.0 Hz, 1H, C(6)*H*), 5.05 (s, 1H, N*H*), 4.89 (br s, 1H, N*H*Boc), 3.67–3.53 (m, 1H, C(2)*H*), 3.38–3.27 (m, 1H, C(1′)H_A), 3.19–3.10 (m, 1H, C(1′)H_B), 2.83–2.65 (m, 2H, C(4)H_2), 1.99–1.87 (m, 1H, C(3)H_A), 1.72–1.58 (m, 1H, C(3)H_B), 1.44 (s, 9H, OC(CH_3)$_3$)

^{13}C NMR: (101 MHz $CDCl_3$)

156.3 (*C*(9)), 156.0 (C=O), 146.2 (*C*(7)), 136.3 (*C*(5)), 116.1 (*C*(10)), 113.5 (*C*(6)), 79.8 (O*C*(CH_3)$_3$), 51.1 (*C*(2)), 45.9 (*C*(1′)), 28.5 (OC(*C*H_3)$_3$), 25.2 (*C*(4)), 24.7 (*C*(3))

IR: (neat)

3370 (w), 3264 (w), 3101 (w), 3019 (w), 2979 (w), 2924 (m), 2955 (w), 2870 (w), 2830 (w), 1685 (s), 1600 (m), 1582 (m), 1519 (s), 1455 (m), 1437 (m), 1387 (m), 1365 (m), 1322 (m), 1269 (s), 1246 (s), 1166 (s), 1115 (m), 1086 (m), 1067 (w), 1035 (w), 996 (m), 937 (m), 886 (w), 859 (w), 781 (w), 767 (m), 716 (w), 668 (w), 612 (m), 570 (m)

HRMS: (ESI^+)

Calcd for $C_{14}H_{21}N_3O_2$: 263.1634, found: 263.1638

Preparation of (*RS*)-2-(2-(1*H*-imidazol-1-yl)ethyl)-1,2,3,4-tetrahydro-1,8-naphthyridine (13q)

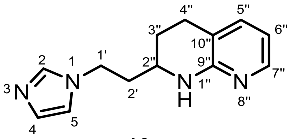

13q

Following the **General Procedure** (section B), 5 mL of reagent feed A [2-fluoro-3-vinylpyridine **17** (185 mg, 1.50 mmol, 1.0 equiv) and 3DPA2FBN (9.6 mg, 15.0 μmol, 1 mol%) in anhydrous DMF], 5 mL of reagent feed B [1-(3-aminopropyl)imidazole (**10q**) (500 mg, 4.50 mmol, 3.0 equiv) and NaN_3 (19.5 mg, 300 μmol, 20 mol%) in anhydrous DMF], and 10 mL of reagent feed C [DIPEA (291 mg, 2.25 mmol, 1.5 equiv) in anhydrous DMF] were reacted in flow, setting the high-temperature tube reactor to 180 °C. The steady state mixture (12 mL) was collected and concentrated *in vacuo* on an Asynt spiral evaporator. Purification *via* automated flash column chromatography on SiO_2 gel (12 g) in CH_2Cl_2 (5 CV) then 100:0:0→95:4.5:0.5 CH_2Cl_2–MeOH–aq. NH_4OH (over 20 CV) then 95:4.5:0.5

CH_2Cl_2–MeOH–aq. NH_4OH (5 CV) gave **13q** as an off-white, crystalline solid (136 mg, 63%, 1.50 mmol h^{-1}).

Data for **13q**:

mp: 89–90 °C

^{1}H NMR: (400 MHz, $CDCl_3$)

7.88 (dd, *J* = 5.0, 1.7 Hz, 1H, C(7″)*H*), 7.49 (s, 1H, C(2)*H*), 7.17 (dd, *J* = 7.2, 1.7 Hz, 1H, C(5″)*H*), 7.10–7.05 (m, 1H, C(4)*H*), 6.95–6.89 (m, 1H, C(5)*H*), 6.48 (dd, *J* = 7.2, 5.0 Hz, 1H, C(6″)*H*), 4.93 (s, 1H, N*H*), 4.15–4.04 (m, 2H, C(1′)*H*), 3.48–3.40 (m, 1H, C(2″)*H*), 2.79–2.66 (m, 2H, C(4″)H_2), 2.10–1.85 (m, 3H, C(2′)H_2, C(3)H_A), 1.72–1.59 (m, 1H, C(3)H_B)

^{13}C NMR: (101 MHz $CDCl_3$)

155.7 (*C*(9″)), 146.3 (*C*(7″)), 137.2 (*C*(2)), 136.5 (*C*(5″)), 130.1 (*C*(4)), 118.8 (*C*(5)), 115.8 (*C*(10″)), 113.6 (*C*(6″)), 48.6 (*C*(2″)), 43.5 (*C*(1′)), 37.8 (*C*(2′)), 26.8 (*C*(3″)), 25.1 (*C*(4″))

IR: (neat)

3233 (w), 3157 (w), 3109 (w), 3022 (w), 3018 (w), 2953 (w), 2932 (w), 2853 (w), 1600 (m), 1579 (m), 1520 (m), 1507 (m), 1444 (m), 1348 (m), 1332 (m), 1276 (m), 1224 (m), 1103 (w), 1074 (m), 906 (w), 819 (m), 776 (m), 758 (m), 747 (s), 663 (m), 626 (m), 590 (m), 570 (m)

HRMS: (ESI^+)

Calcd for $C_{13}H_{16}N_4$: 228.1375, found: 162.1160

Preparation of 2,3,3′,4′,5,6-hexahydro-1′*H*-spiro[pyran-4,2′-[1,6]naphthyridine] (14a)

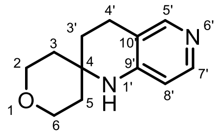

14a

Following the **General Procedure** (section B), 5 mL of reagent feed A [4-chloro-3-vinylpyridine **18** (209 mg, 1.50 mmol, 1.0 equiv) and 3DPA2FBN (9.6 mg, 15.0 µmol, 1 mol%) in anhydrous DMF], 5 mL of reagent feed B [tetrahydro-2*H*-pyran-4-amine **10g** (152 mg, 1.50 mmol, 1.0 equiv) and NaN_3 (19.5 mg, 300 µmol, 20 mol%) in anhydrous DMF], and 10 mL of reagent feed C [DIPEA (291 mg, 2.25 mmol, 1.5 equiv) in anhydrous DMF] were reacted in flow, setting the high-temperature tube reactor to 220 °C. The steady state mixture (8.8 mL) was collected and concentrated *in vacuo* on an

Asynt spiral evaporator. Purification *via* automated flash column chromatography on SiO_2 gel (12 g) in CH_2Cl_2 (5 CV) then 100:0:0→95:4.5:0.5 CH_2Cl_2–MeOH–aq. NH_4OH (over 20 CV) then 95:4.5:0.5 CH_2Cl_2–MeOH–aq. NH_4OH (5 CV), followed by automated flash column chromatography on C_{18} reversed-phase silica gel (50 g) in H_2O (5 CV) then 100:0→0:100 H_2O–MeOH (over 20 CV) then MeOH (10 CV) gave **14a** as a colourless, crystalline solid (75.3 mg, 46%, 1.25 mmol h^{-1}).

Data for **14a**:

mp: 165–167 °C

^{1}H NMR: (400 MHz, $CDCl_3$)

8.05 (s, 1H, C(5′)*H*), 8.01 (d, *J* = 5.6 Hz, 1H, C(7′)*H*), 6.36 (d, *J* = 5.6 Hz, 1H, C(6′)*H*), 4.47 (s, 1H, N*H*), 3.84–3.68 (m, 4H, C(2)H_2, C(6)H_2), 2.73 (t, *J* = 6.7 Hz, 2H, C(4′)H_2), 1.82 (t, *J* = 6.7 Hz, 2H, C(3′)H_2), 1.78–1.70 (m, 2H, C(3)H_A, C(5)H_A), 1.66–1.58 (m, 2H, C(3)H_B, C(5)H_B)

^{13}C NMR: (101 MHz $CDCl_3$)

149.5 (*C*(5′)), 149.1 (*C*(9′)), 147.7 (*C*(7′)), 115.7 (*C*(10′)), 108.7 (*C*(6′)), 63.7 (*C*(2), *C*(6)), 48.8 (*C*(4)), 37.8 (*C*(3), *C*(5)), 31.5 (*C*(3′)), 20.1 (*C*(4′))

IR: (neat)

3221 (w), 3156 (w), 3049 (w), 2934 (m), 2858 (m), 2840 (m), 1607 (m), 1586 (m), 1514 (m), 1486 (w), 1470 (w), 1439 (w), 1427 (m), 1384 (m), 1338 (m), 1305 (m), 1288 (m), 1254 (m), 1246 (m), 1219 (m), 1180 (m), 1172 (m), 1135 (m), 1102 (s), 1085 (m), 1053 (s), 1026 (m), 1026 (m), 1007 (m), 979 (m), 947 (w), 912 (w), 900 (m), 875 (w), 842 (m), 821 (w), 762 (w), 714 (m), 645 (m), 628 (m), 597 (m), 569 (m), 559 (m)

HRMS: (ESI^+)

Calcd for $C_{12}H_{16}N_2O$: 204.1263, found: 204.1272

Preparation of 2,3,3′,4′,5,6-hexahydro-1′*H*-spiro[thiopyran-4,2′-[1,6]naphthyridine] (14b)

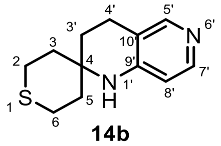

14b

Following the **General Procedure** (section B), 5 mL of reagent feed A [4-chloro-3-vinylpyridine **18** (209 mg, 1.50 mmol, 1.0 equiv) and 3DPA2FBN (9.6 mg, 15.0 μmol, 1 mol%) in anhydrous DMF], 5 mL of reagent feed B [tetrahydro-2*H*-thiopyran-4-amine **10h** (176 mg, 1.50 mmol, 1.0 equiv) and NaN_3 (19.5 mg, 300 μmol, 20 mol%) in anhydrous DMF], and 10 mL of reagent feed C [DIPEA (291

mg, 2.25 mmol, 1.5 equiv) in anhydrous DMF] were reacted in flow, setting the high-temperature tube reactor to 220 °C. The steady state mixture (10.5 mL) was collected and concentrated *in vacuo* on an Asynt spiral evaporator. Purification *via* automated flash column chromatography on SiO_2 gel (12 g) in CH_2Cl_2 (5 CV) then 100:0:0→95:4.5:0.5 CH_2Cl_2–MeOH–aq. NH_4OH (over 20 CV) then 95:4.5:0.5 CH_2Cl_2–MeOH–aq. NH_4OH (5 CV), followed by automated flash column chromatography on C_{18} reversed-phase silica gel (50 g) in H_2O (5 CV) then 100:0→0:100 H_2O–MeOH (over 20 CV) then MeOH (10 CV) gave **14b** as a pale yellow, crystalline solid (110 mg, 46%, 1.44 mmol h^{-1}).

Data for **14b**:

mp: 170–171 °C

^{1}H NMR: (400 MHz, $CDCl_3$)

8.04 (s, 1H, C(5′)*H*), 8.00 (d, *J* = 5.6 Hz, 1H, C(7′)*H*), 6.37 (d, *J* = 5.6 Hz, 1H, C(8′)*H*), 4.47 (s, 1H, N*H*), 2.77–2.63 (m, 6H, C(4′)H_2, C(2)H_2, C(6)H_2), 1.94–1.81 (m, 4H, C(3)H_2, C(5)H_2), 1.77 (t, *J* = 6.7 Hz, 2H, C(3′)H_2)

^{13}C NMR: (101 MHz $CDCl_3$)

149.3 (*C*(5′)), 149.1 (*C*(9′)), 147.5 (*C*(7′)), 115.5 (*C*(10′)), 108.7 (*C*(8′)), 49.8 (*C*(4)), 38.4 (*C*(3), *C*(5)), 32.0 (*C*(3′)), 24.2 (*C*(2), *C*(6)), 19.8 (*C*(4′))

IR: (neat)

3224 (w), 3154 (w), 2942 (m), 2926 (m), 2906 (m), 2840 (m), 1603 (s), 1586 (m), 1509 (m), 1486 (m), 1447 (m), 1425 (m), 1337 (m), 1302 (m), 1269 (m), 1242 (m), 1197 (m), 1180 (w), 1165 (w), 1108 (m), 1070 (m), 1049 (m), 1028 (m), 1010 (m), 980 (m), 965 (m), 953 (m), 923 (m), 901 (m), 861 (m), 826 (m), 819 (m), 788 (m), 760 (m), 731 (m), 634 (s), 611 (s), 579 (m), 562 (m), 555 (m)

HRMS: (ESI^+)

Calcd for $C_{12}H_{16}N_2S$: 220.1034, found: 220.1040

Preparation of *tert*-butyl 3′,4′-dihydro-1′*H*-spiro[piperidine-4,2′-[1,6]naphthyridine]-1-carboxylate (14c)

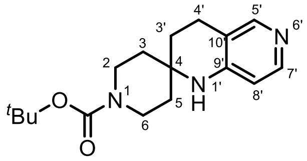

14c

Following the **General Procedure** (section B), 5 mL of reagent feed A [4-chloro-3-vinylpyridine **18** (209 mg, 1.50 mmol, 1.0 equiv) and 3DPA2FBN (9.6 mg, 15.0 μmol, 1 mol%) in anhydrous DMF], 5 mL of reagent feed B [*tert*-butyl 4-aminopiperidine-1-carboxylate **10i** (300 mg, 1.50 mmol, 1.0 equiv) and NaN_3 (19.5 mg, 300 μmol, 20 mol%) in anhydrous DMF], and 10 mL of reagent feed C [DIPEA (291 mg, 2.25 mmol, 1.5 equiv) in anhydrous DMF] were reacted in flow, setting the high-temperature tube reactor to 220 °C. The steady state mixture (12 mL) was collected and concentrated *in vacuo* on an Asynt spiral evaporator. Purification *via* automated flash column chromatography on SiO_2 gel (12 g) in CH_2Cl_2 (5 CV) then 100:0:0→95:4.5:0.5 CH_2Cl_2–MeOH–aq. NH_4OH (over 20 CV) then 95:4.5:0.5 CH_2Cl_2–MeOH–aq. NH_4OH (5 CV), followed by automated flash column chromatography on C_{18} reversed-phase silica gel (50 g) in H_2O (5 CV) then 100:0→0:100 H_2O–MeOH (over 20 CV) then MeOH (10 CV) gave **14c** as a colourless, crystalline solid (134 mg, 50%, 1.12 mmol h^{-1}).

Data for **14c**:

mp: 143–145 °C

^{1}H NMR: (400 MHz, $CDCl_3$)

8.03 (s, 1H, C(5′)*H*), 7.99 (d, *J* = 5.6 Hz, 1H, C(7′)*H*), 6.36 (d, *J* = 5.6 Hz, 1H, C(8′)*H*), 4.55 (s, 1H, N*H*), 3.66–3.53 (m, 2H, C(2)H_A, C(6)H_A), 3.42–3.34 (m, 2H, C(2)H_A, C(6)H_A), 2.72 (t, *J* = 6.6 Hz, 2H, C(4′)H_2), 1.78 (t, *J* = 6.6 Hz, 2H, C(4′)H_2), 1.69–1.53 (m, 4H, C(3)H_2, C(5)H_2), 1.46 (s, 9H, OC(CH_3)$_3$)

^{13}C NMR: (101 MHz $CDCl_3$)

154.9 (*C*=O), 149.5 (*C*(9′)), 148.8 (*C*(5′)), 147.1 (*C*(7′)), 115.6 (*C*(10′)), 108.7 (*C*(6′)), 79.9 (O*C*(CH_3)$_3$), 49.7 (*C*(4)), 39.6 (br s, *C*(2), *C*(6)), 36.8 (*C*(3), *C*(5)), 30.8 (*C*(3′)), 28.6 (OC(*C*H_3)$_3$), 20.2 (*C*(4′))

IR: (neat)

3232 (w), 2932 (w), 1670 (m), 1603 (m), 1510 (m), 1477 (w), 1422 (m), 1364 (m), 1340 (w), 1302 (w), 1277 (m), 1263 (m), 1249 (m), 1213 (w), 1152 (s), 1079 (m), 1054 (m), 1025 (m), 998 (w), 981 (w), 959 (w), 908 (w), 873 (m), 822 (m), 748 (m), 728 (m), 694 (w), 643 (m), 609 (w), 601 (w), 585 (w), 578 (m), 562 (m)

HRMS: (ESI+)

Calcd for $C_{17}H_{25}N_3O_2$: 303.1947, found: 303.1945

Preparation of *tert*-butyl 3′,4′-dihydro-1′*H*-spiro[azetidine-3,2′-[1,6]naphthyridine]-1-carboxylate (14d)

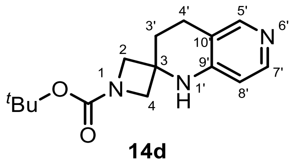

14d

Following the **General Procedure** (section B), 5 mL of reagent feed A [4-chloro-3-vinylpyridine **18** (209 mg, 1.50 mmol, 1.0 equiv) and 3DPA2FBN (9.6 mg, 15.0 μmol, 1 mol%) in anhydrous DMF], 5 mL of reagent feed B [*tert*-butyl 3-aminoazetidine-1-carboxylate **10j** (775 mg, 4.50 mmol, 3.0 equiv) and NaN_3 (19.5 mg, 300 μmol, 20 mol%) in anhydrous DMF], and 10 mL of reagent feed C [DIPEA (291 mg, 2.25 mmol, 1.5 equiv) in anhydrous DMF] were reacted in flow, setting the high-temperature tube reactor to 220 °C. The steady state mixture (12 mL) was collected and concentrated *in vacuo* on an Asynt spiral evaporator. Purification *via* automated flash column chromatography on SiO_2 gel (12 g) in CH_2Cl_2 (5 CV) then 100:0:0→95:4.5:0.5 CH_2Cl_2–MeOH–aq. NH_4OH (over 20 CV) then 95:4.5:0.5 CH_2Cl_2–MeOH–aq. NH_4OH (5 CV), followed by automated flash column chromatography on C_{18} reversed-phase silica gel (50 g) in H_2O (5 CV) then 100:0→0:100 H_2O–MeOH (over 20 CV) then MeOH (10 CV) gave **14d** as a colourless, crystalline solid (145 mg, 59%, 1.32 mmol h^{-1}).

Data for **14d**:

mp: 150–151 °C

^{1}H NMR: (400 MHz, $CDCl_3$)

8.04 (s, 1H, C(5′)*H*), 8.02 (d, *J* = 5.6 Hz, 1H, C(7′)*H*), 6.36 (d, *J* = 5.6 Hz, 1H, C(8′)*H*), 6.08 (s, 1H, N*H*), 3.87 (d, *J* = 8.9 Hz, 2H, C(2)H_A, C(4)H_A), 3.86 (d, *J* = 8.9 Hz, 2H, C(2)H_B, C(4)H_B), 2.73 (t, *J* = 6.4 Hz, 2H, C(4′)H_2), 2.05 (t, *J* = 6.4 Hz, 2H, C(3′)H_2), 1.44 (s, 9H, OC(CH_3)$_3$)

^{13}C NMR: (101 MHz $CDCl_3$)

156.5 (*C*=O), 149.1 (*C*(5′)), 148.7 (*C*(9′)), 148.0 149.1 (*C*(7′)), 115.8 (*C*(10′)), 108.5 (*C*(8′)), 80.1 (O*C*(CH_3)$_3$), 63.2 (br s, *C*(2), *C*(4)), 50.5 (*C*(3)), 30.1 (*C*(3′)), 28.5 (OC(*C*H_3)$_3$) , 23.8 (*C*(4′))

<u>IR:</u> (neat)

3335 (w), 2976 (w), 2874 (w), 1683 (m), 1603 (m), 1511 (w), 1476 (w), 1403 (m), 1366 (m), 1334 (w), 1280 (w), 1250 (w), 1225 (w), 1143 (m), 1083 (m), 1053 (m), 999 (w), 911 (w), 856 (w), 820 (w), 770 (w), 727 (s), 644 (m), 603 (w), 569 (m)

<u>HRMS:</u> (ESI$^+$)

Calcd for $C_{15}H_{21}N_3O_2$: 275.1634, found: 275.1636

Preparation of 3′,4′-dihydro-1′*H*-spiro[oxetane-3,2′-[1,6]naphthyridine] (14e)

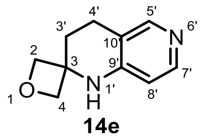

14e

Following the **General Procedure** (section B), 5 mL of reagent feed A [4-chloro-3-vinylpyridine **18** (209 mg, 1.50 mmol, 1.0 equiv) and 3DPA2FBN (9.6 mg, 15.0 μmol, 1 mol%) in anhydrous DMF], 5 mL of reagent feed B [oxetan-3-amine **10k** (329 mg, 4.50 mmol, 3.0 equiv) and NaN_3 (19.5 mg, 300 μmol, 20 mol%) in anhydrous DMF], and 10 mL of reagent feed C [DIPEA (291 mg, 2.25 mmol, 1.5 equiv) in anhydrous DMF] were reacted in flow, setting the high-temperature tube reactor to 220 °C. The steady state mixture (5.6 mL) was collected and concentrated *in vacuo* on an Asynt spiral evaporator. Purification *via* automated flash column chromatography on SiO_2 gel (12 g) in CH_2Cl_2 (5 CV) then 100:0:0→95:4.5:0.5 CH_2Cl_2–MeOH–aq. NH_4OH (over 20 CV) then 95:4.5:0.5 CH_2Cl_2–MeOH–aq. NH_4OH (5 CV), followed by automated flash column chromatography on C_{18} reversed-phase silica gel (50 g) in H_2O (5 CV) then 100:0→0:100 H_2O–MeOH (over 20 CV) then MeOH (10 CV) gave **14e** as a colourless, crystalline solid (40.8 mg, 46%, 1.03 mmol h^{-1}).

<u>Data for **14d**:</u>

<u>mp:</u> 153–155 °C

<u>^{1}H NMR:</u> (400 MHz, $CDCl_3$)

8.04 (s, 1H, C(5′)*H*), 8.03 (d, *J* = 5.6 Hz, 1H, C(7′)*H*), 6.38 (d, *J* = 5.6 Hz, 1H, C(8′)*H*), 5.03 (s, 1H, N*H*), 4.66–4.60 (m, 2H, C(2)H_A, C(4)H_A), 4.56–4.49 (m, 2H, C(2)H_B, C(4)H_B), 2.72 (t, *J* = 6.7 Hz, 2H, C(4′)H_2), 2.20 (t, *J* = 6.4 Hz, 2H, C(3′)H_2)

<u>^{13}C NMR:</u> (101 MHz $CDCl_3$)

148.7 (*C*(9′)), 148.4 (*C*(5′)), 147.5 (*C*(7′)), 115.9 (*C*(10′)), 108.3 (*C*(8′)), 55.0 (*C*(2), *C*(4)), 29.1 (*C*(3′)), 20.6 (*C*(4′))

IR: (neat)

3338 (m), 3160 (w), 3018 (w), 2944 (m), 2831 (m), 1609 (s), 1595 (m), 1523 (m), 1466 (m), 1450 (s), 1401 (s), 1359 (m), 1288 (m), 1267 (m), 1192 (m), 1132 (m), 1032 (w), 975 (s), 925 (m), 880 (m), 779 (m), 743 (s), 715 (m), 630 (m), 595 (m), 567 (m)

HRMS: (ESI^+)

Calcd for $C_{10}H_{12}N_2O$: 176.0950, found: 176.0955

F. Gram-Scale Synthesis of THN 13i

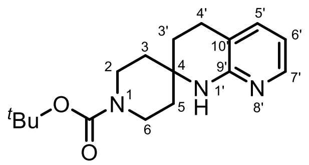

13i

Following the **General Procedure** (section B), 25 mL of reagent feed A [2-fluoro-3-vinylpyridine **17** (924 mg, 7.50 mmol, 1.0 equiv) and 3DPA2FBN (48.2 mg, 7.50 mmol, 1 mol%) in anhydrous DMF], 25 mL of reagent feed B [*tert*-butyl 4-aminopiperidine-1-carboxylate **10i** (1.50 g, 22.5 mmol, 1.0 equiv) and NaN_3 (998 mg, 1.50 mmol, 20 mol%) in anhydrous DMF], and 50 mL of reagent feed C [DIPEA (1.45 g, 22.5 mmol, 1.5 equiv) in anhydrous DMF] were reacted in flow, setting the high-temperature tube reactor to 200 °C. The steady state mixture (94 mL) was collected and concentrated *in vacuo*. Purification *via* automated flash column chromatography on SiO_2 gel (24 g) in 40–60 °C petroleum ether (5 CV) then 100:0→0:100 40–60 °C petroleum ether–EtOAc (over 20 CV) then EtOAc (5 CV) gave **13i** as a colourless, crystalline solid (1.85 g, 87%, 1.98 mmol h^{-1}).

G. Resolution of Chiral, Racemic THN 13n by Prep HPLC

Analysis

The sample of THN **13n** was prepared at approximately 1 mg/mL in MeOH. The chiral SFC screen was run at AstraZeneca on UPC2 (11 stationary phases and 1 solvent – MeOH-NH_3).

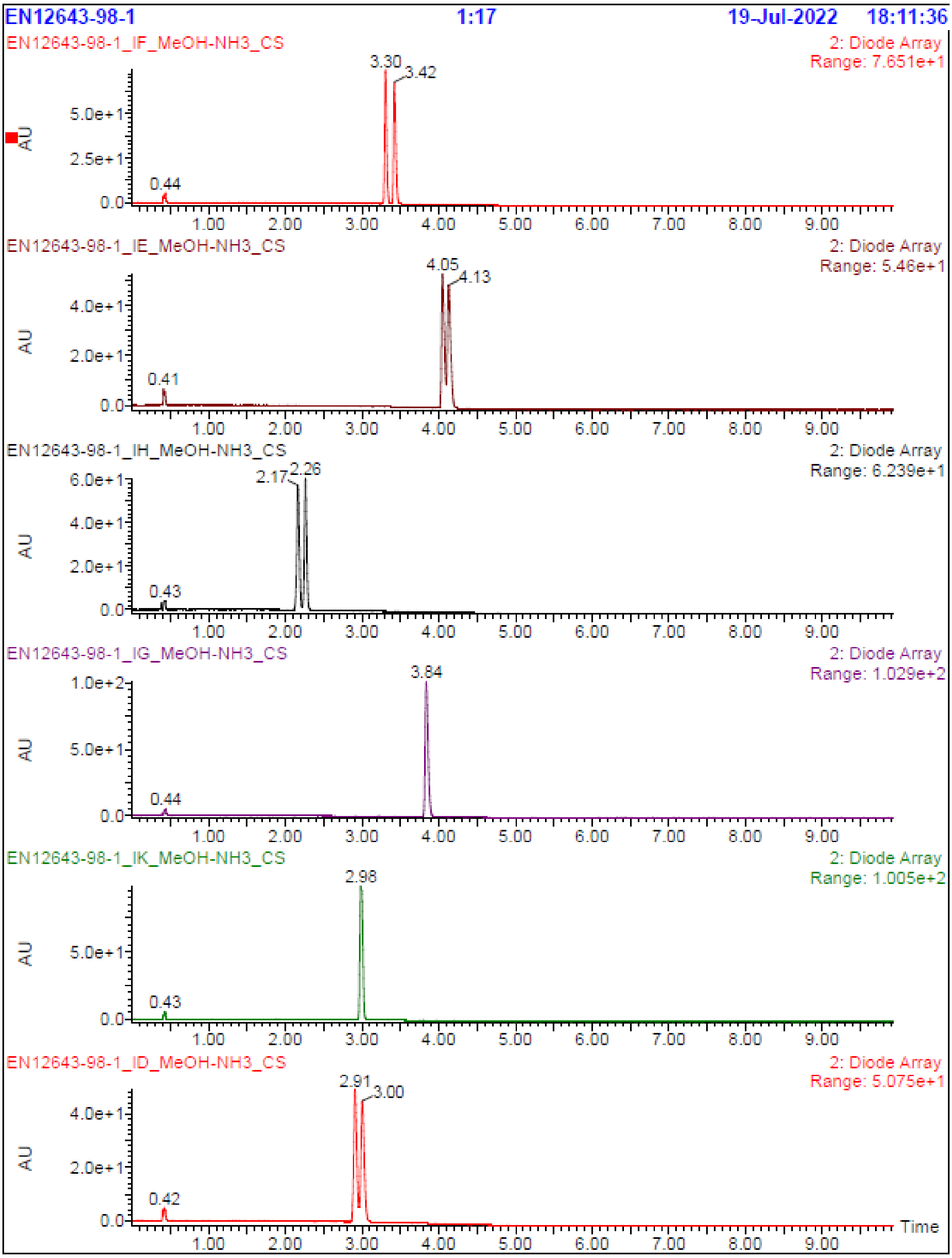

Supplementary Figure 4. Screening chiral SFC stationary phases for racemate separation

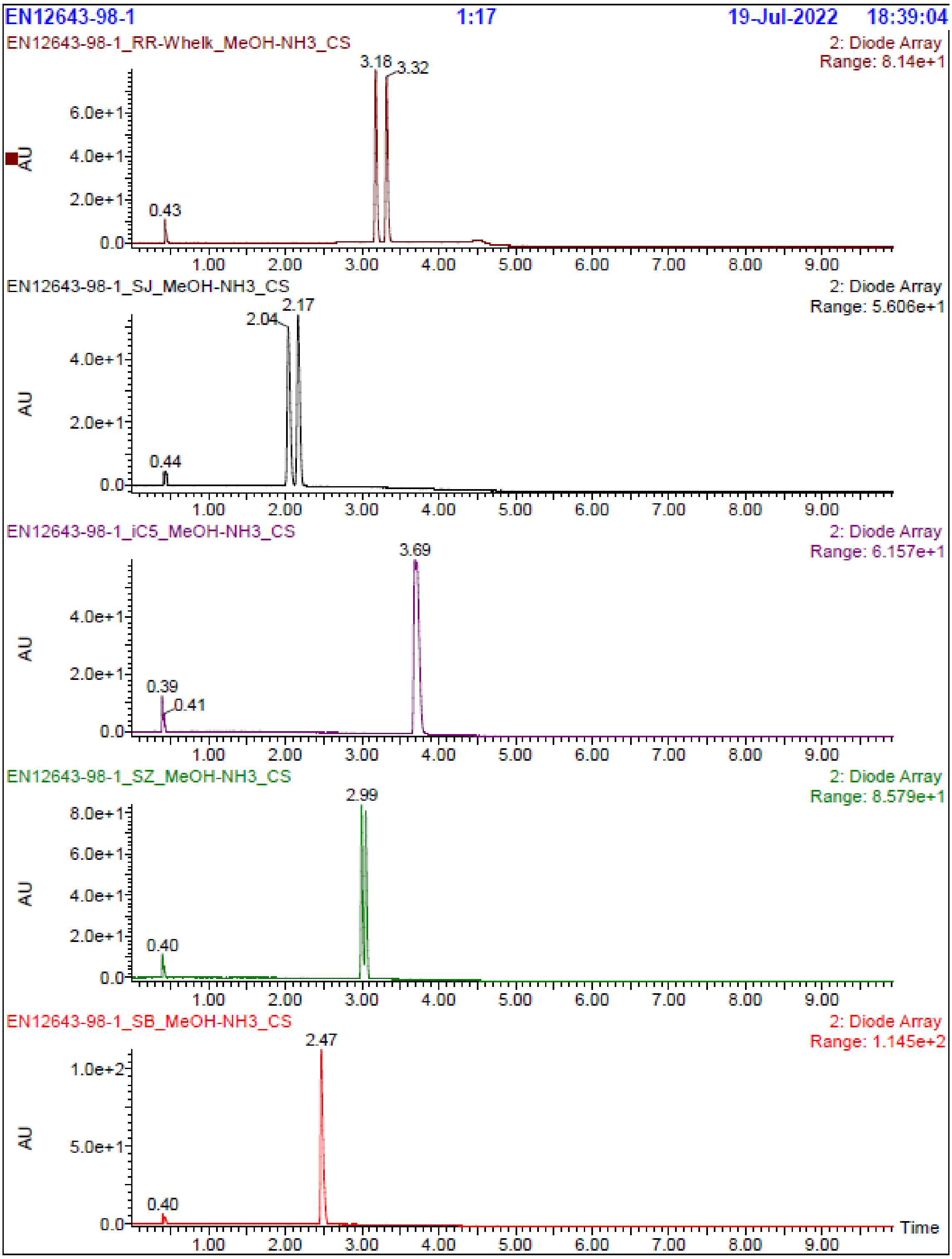

Supplementary Figure 4 (cont). Screening chiral SFC stationary phases for racemate separation

Prep SFC Report

**Samplename:**	en12643-98_02		
**Run Started:**	8/17/2022 2:00:59 PM	**Reported:**	8/17/2022 4:20:37 PM

**Method:**	Whelk_RR_15_MeOH		
**Injection:**	1 / 1	**Wavelength [nm]:**	220
**Injectionvolume [ml]:**	0.25	**Modifier:**	C
**Flow [ml/min]:**	60	**Modifierpercent:**	15
**Column:**	Whelk-O RR 20mm		

System Settings

**Backpressure [bar]:**	120	**Temp Pumphead [°C]:**	10
**Temp Fraction Module [°C]:**	20	**Temp Columndepartment [°C]:**	40

Chromatogram

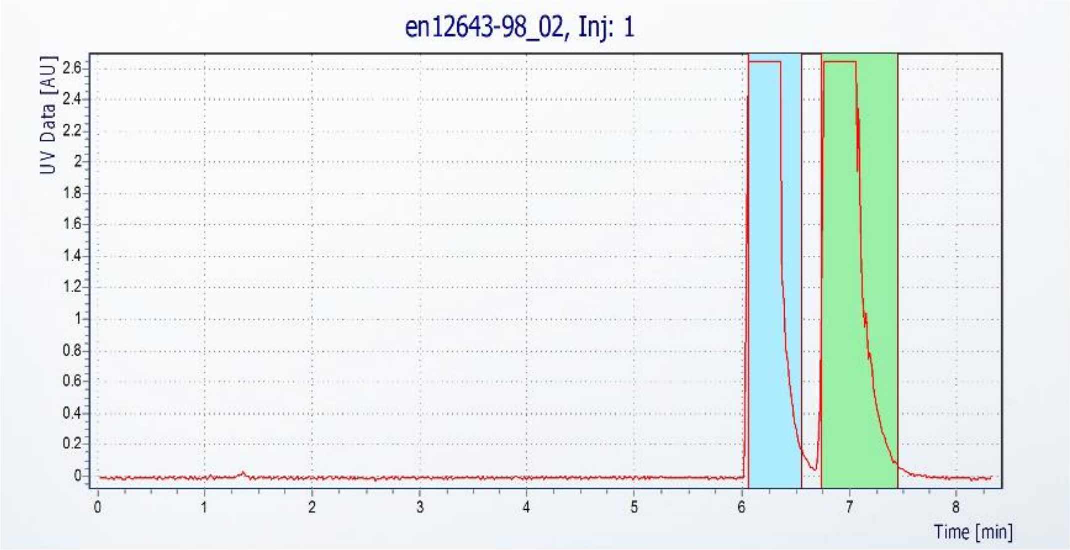

Peak Number	Peak Start	Peak Stop	Vial No.
1	6.06	6.56	2
2	6.74	7.46	3

Supplementary Figure 5. Prep chiral SFC report

sepiatec

Prep SFC Report

**Samplename:**	en12643-98_04		
**Run Started:**	8/17/2022 2:33:15 PM	**Reported:**	8/17/2022 4:18:57 PM
**Method:**	Whelk_RR_15_MeOH		
**Injection:**	1 / 20	**Wavelength [nm]:**	220
**Injectionvolume [ml]:**	0.25	**Modifier:**	C
**Flow [ml/min]:**	60	**Modifierpercent:**	15
**Column:**	Whelk-O RR 20mm		

System Settings

**Backpressure [bar]:**	120	**Temp Pumphead [°C]:**	10
**Temp Fraction Module [°C]:**	20	**Temp Columndepartment [°C]:**	40

Chromatogram

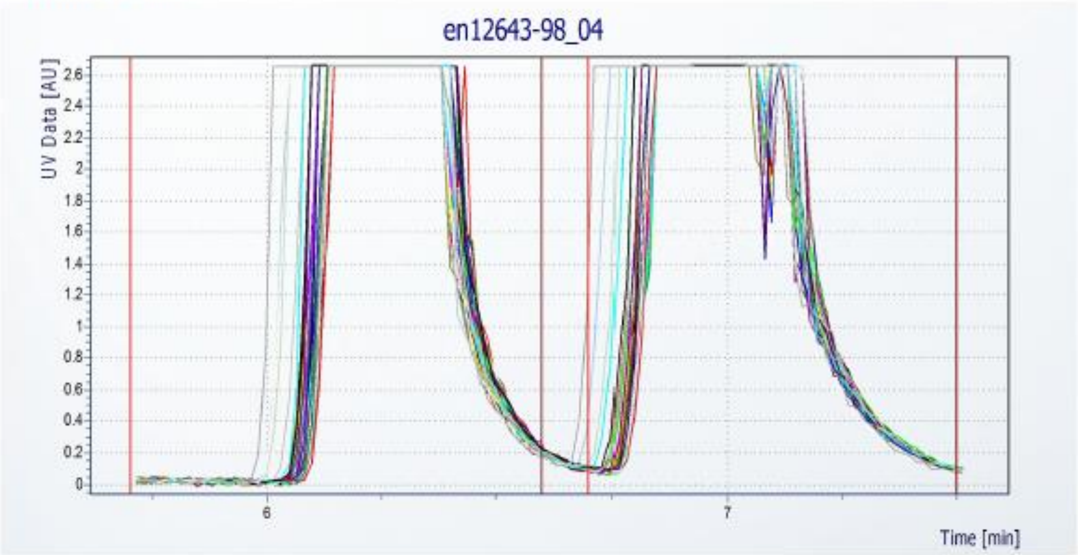

Peak Number	Peak Start	Peak Stop	Vial No.
1	5.70	6.60	2
2	6.70	7.50	3

Supplementary Figure 6. Prep chiral SFC report

Resolution

The sample of THN **13n** (520 mg) was dissolved in CH_2Cl_2:MeOH (8 mL) and filtered. The solution was then purified using the SFC conditions detailed below (retention times: P1 = 6.25 and P2 = 7.0 min):

Column: Regis (R,R) Whelk, 30 × 250 mm, 5 micron
Mobile phase: 15% MeOH + 0.1% NH_3, 85% $scCO_2$
Flow rate: 60 mL/min
BPR: 120 bar
Column temperature: 40 °C
UV max 220 nm
Stacked injections

The pooled fractions were concentrated *in vacuo* on a Genevac Rocket Synergy™ 2 evaporator system (low boiling point method) into vials followed by evaporation on a Biotage® V-10 Touch evaporator system (high boiling point method).

QC analysis was carried using the following conditions:

Column: Regis (R,R) Whelk, 3 × 150 mm, 3.5 micron
Mobile phase: A = $scCO_2$, B MeOH + 0.1% NH_3. Gradient 0-1 min 5% B, 1-5 min 5-50% B, 5-10
Flow rate: 2.0 mL/min
BPR: 120 bar
Temperature: 40 °C

<u>Peak 1</u>:

Final weight: 7545.9 mg
Tare weight: 7286.3 mg
Weight of material isolated: 260 mg
Purity (SFC): >99% pure, >99% ee

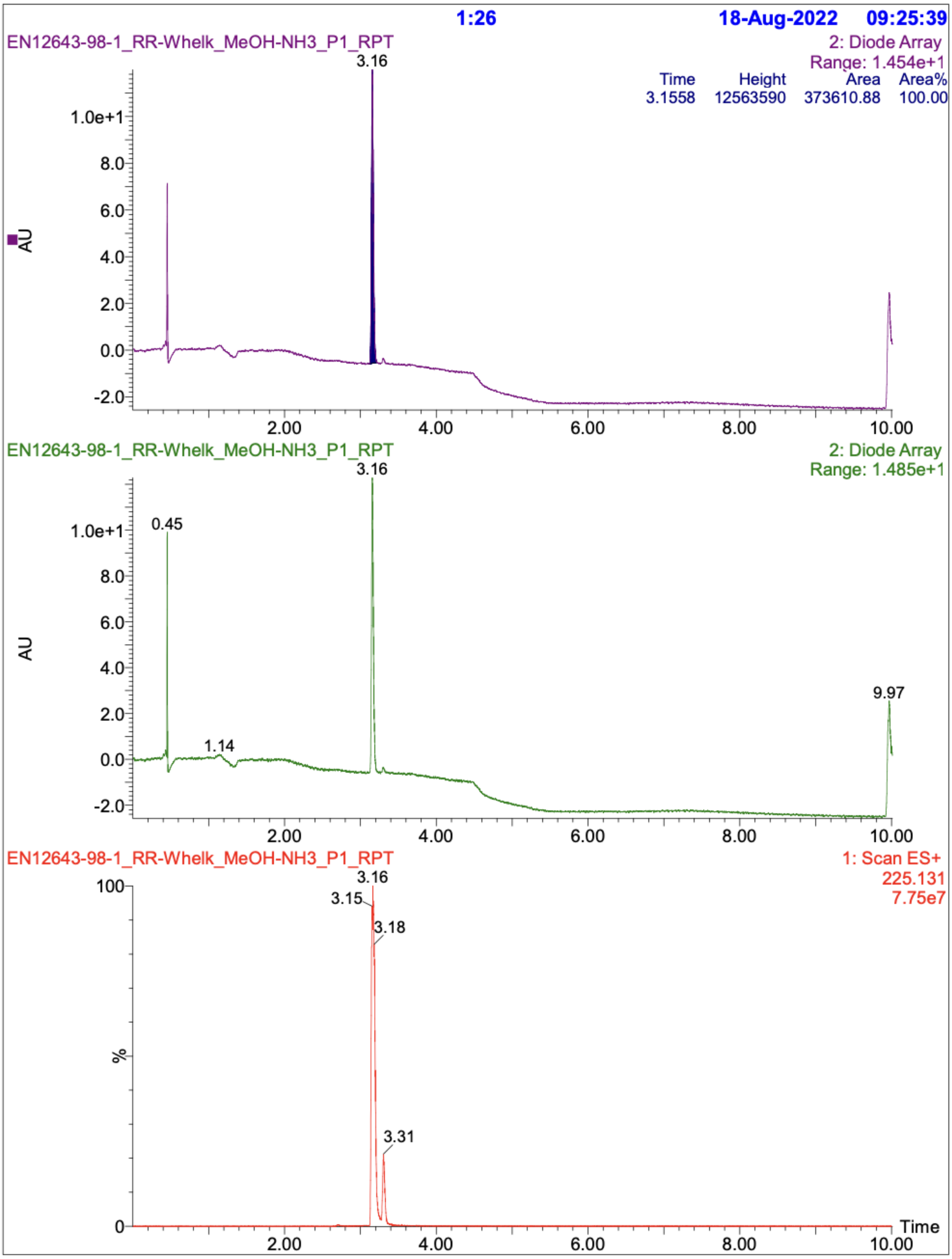

Supplementary Figure 7. Prep chiral SFC traces for peak 1

Peak 2:

Final weight: 7655.7 mg
Tare weight: 7403.6 mg
Weight of material isolated: 252 mg
Purity (SFC): >99% pure, >99% ee

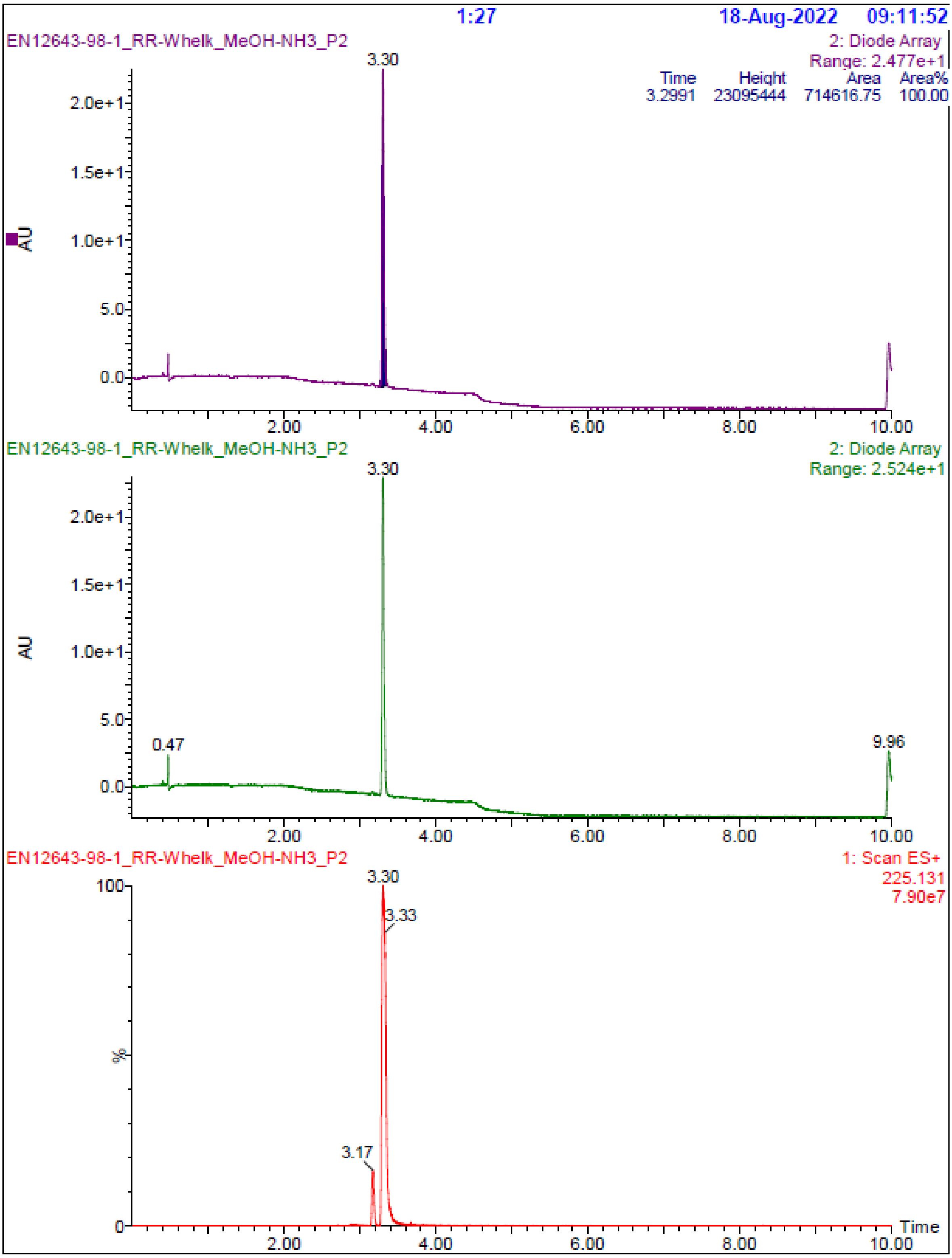

Supplementary Figure 8. Prep chiral SFC traces for peak 2

H. C–H Functionalisation of the Pyridine Ring of THN 13i at C(6)

Preparation of *tert*-butyl 6′-chloro-3′,4′-dihydro-1′*H*-spiro[piperidine-4,2′-[1,8]naphthyridine]-1-carboxylate (19)

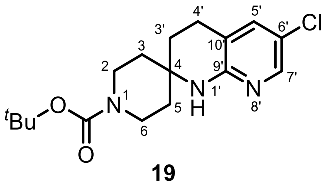

19

A 10-mL microwave vial equipped with a magnetic stirrer bar was charged with **13i** (100 mg, 0.33 mmol, 1.0 equiv), NCS (44.0 mg, 0.33 mmol, 1.0 equiv), and MeCN (1.5 mL). The vial was then capped with an aluminium crimp seal and was stirred at 80 °C in a silicone oil bath for 16 h. H_2O (5 mL) and EtOAc (15 mL) were added and the mixture was washed sequentially with sat. $NaHCO_3$ (15 mL) and sat. brine (15 mL), then dried over Na_2SO_4, and concentrated *in vacuo*. Purification *via* automated flash column chromatography on SiO_2 gel (12 g) in 40–60 °C petroleum ether (5 CV) then 100:0→0:100 40–60 °C petroleum ether–EtOAc (over 20 CV) then EtOAc (5 CV) gave **19** as a colourless, crystalline solid (100 mg, 90%).

Data for **19**:

mp: 133–134 °C

^{1}H NMR: (400 MHz, $CDCl_3$)

7.83 (d, *J* = 1.7 Hz, 1H, C(7′)*H*), 7.21 (d, *J* = 1.7 Hz, 1H, C(5′)*H*), 5.24 (s, 1H, N*H*), 3.73–3.63 (m, 2H, C(2)H_A, C(6)H_A), 3.37–3.25 (m, 2H, C(2)H_B, C(6)H_B), 2.74 (t, *J* = 6.7 Hz, 2H, C(4′)H_2), 1.74 (t, *J* = 6.7 Hz, 2H, C(3′)H_2), 1.65–1.58 (m, 4H, C(3)H_2, C(5)H_2), 1.46 (s, 9H, OC(CH_3)$_3$)

^{13}C NMR: (101 MHz $CDCl_3$)

154.9 (*C*=O), 153.6 (*C*(9′)), 144.5 (*C*(7′)), 136.1 (*C*(5′)), 119.6 (*C*(6′)), 116.6 (*C*(10′)), 79.9 (O*C*(CH_3)$_3$), 50.2 (*C*(4)), 39.7 (br s, *C*(2), *C*(6)), 36.9 (*C*(3), *C*(5)), 31.4 (*C*(3′)), 28.6 (OC(*C*H_3)$_3$), 22.6 (*C*(4′))

IR: (neat)

3401 (w), 3041 (w), 2991 (w), 2976 (w), 2934 (w) 2782 (w), 1681 (s), 1589 (m), 1574 (w), 1486 (s), 1463 (m), 1435 (s), 1424 (s), 1411 (m), 1389 (w), 1363 (m), 1278 (m), 1266 (m), 1252 (s), 1208 (w), 1168 (m), 1154 (s), 1121 (m), 1093 (m), 1078 (m), 1040 (w), 1022 (m), 992 (m), 981 (m), 957 (w), 944 (w), 921 (w), 912 (m), 886 (w), 862 (m), 825 (w), 766 (m), 756 (m), 722 (w), 674 (m), 608 (w), 581 (m), 561 (w), 551 (m)

HRMS: (ESI$^+$)

Calcd for $C_{17}H_{24}ClN_3O_2$: 337.1557, found: 337.1564

Preparation of *tert*-butyl 6′-chloro-3′,4′-dihydro-1′*H*-spiro[piperidine-4,2′-[1,8]naphthyridine]-1-carboxylate (20)

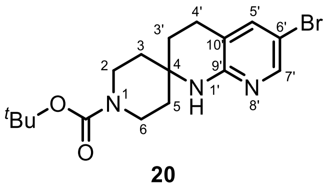

20

A 10-mL microwave vial equipped with a magnetic stirrer bar was charged with **13i** (100 mg, 0.33 mmol, 1.0 equiv), NBS (62.2 mg, 0.35 mmol, 1.06 equiv), and MeCN (2.0 mL). The vial was then capped with an aluminium crimp seal and was stirred at rt for 16 h. H_2O (5 mL) and EtOAc (15 mL) were added and the mixture was washed sequentially with sat. $NaHCO_3$ (15 mL) and sat. brine (15 mL), then dried over Na_2SO_4, and concentrated *in vacuo*. Purification *via* automated flash column chromatography on SiO_2 gel (12 g) in 40–60 °C petroleum ether (5 CV) then 100:0→0:100 40–60 °C petroleum ether–EtOAc (over 20 CV) then EtOAc (5 CV) gave **20** as a colourless, crystalline solid (115 mg, 92%).

Data for **20**:

mp: 147–148 °C

^{1}H NMR: (400 MHz, $CDCl_3$)

7.92 (d, J = 2.3 Hz, 1H, C(7′)*H*), 7.31 (d, J = 2.3 Hz, 1H, C(5′)*H*), 5.15 (s, 1H, N*H*), 3.73–3.61 (m, 2H, C(2)H_A, C(6)H_A), 3.35–3.24 (m, 2H, C(2)H_B, C(6)H_B), 2.73 (t, J = 6.7 Hz, 2H, C(4′)H_2), 1.74 (t, J = 6.7 Hz, 2H, C(3′)H_2), 1.65–1.58 (m, 4H, C(3)H_2, C(5)H_2), 1.46 (s, 9H, OC(CH_3)$_3$)

^{13}C NMR: (101 MHz $CDCl_3$)

154.9 (*C*=O), 153.6 (*C*(9′)), 146.8 (*C*(7′)), 138.6 (*C*(5′)), 117.2 (*C*(10′)), 106.9 (*C*(6′)), 79.9 (O*C*(CH_3)$_3$), 50.2 (*C*(4)), 39.6 (br s, *C*(2), *C*(6)), 36.9 (*C*(3), *C*(5)), 31.4 (*C*(3′)), 28.6 (OC(*C*H_3)$_3$), 22.6 (*C*(4′))

IR: (neat)

3396 (w), 3036 (w), 2989 (w), 2974 (w), 2931 (w), 1680 (s), 1585 (m), 1572 (m), 1485 (s), 1461 (w), 1435 (s), 1423 (s), 1406 (m), 1388 (m), 1335 (w), 1287 (s), 1266 (m), 1251 (s), 1208 (w), 1166 (m), 1153 (s), 1123 (m), 1093 (m), 1077 (s), 1039 (w), 1021

(m), 990 (m), 979 (m), 956 (w), 946 (w), 923 (m), 913 (w), 861 (m), 825 (w), 765 (m), 756 (m), 722 (w), 665 (m), 602 (m), 581 (m), 568 (w)

HRMS: (ESI$^+$)

Calcd for $C_{17}H_{24}BrN_3O_2$: 381.1052, found: 381.1041

Preparation of *tert*-butyl 5′-(4,4,5,5-tetramethyl-1,3,2-dioxaborolan-2-yl)-3′,4′-dihydro-1′*H*-spiro[piperidine-4,2′-[1,8]naphthyridine]-1-carboxylate (21)

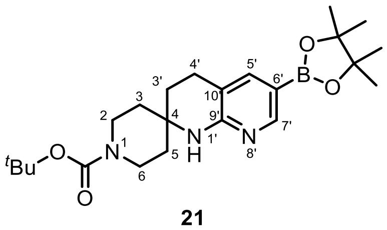

21

In a nitrogen purge box, a 10-mL microwave vial equipped with a magnetic stirrer bar was charged with **13i** (303 mg, 1.00 mmol, 1.0 equiv), HBpin (154 mg, 1.20 mmol, 1.2 equiv), and THF (1.0 mL). Still inside the purge box, the mixture was stirred for 1 h, followed by the addition of $[Ir(OMe)COD]_2$ (10.0 mg, 0.015 mmol, 1.5 mol%), 3,4,7,8-tetramethyl-1,10-phenanthroline (7.1 mg, 0.030 mmol, 3.0 mol%) and B_2pin_2 (127 mg, 0.5 mmol, 0.5 equiv). The mixture turned black when all reagents had been added. The vial was then capped with an aluminium crimp seal and removed from the purge box, then stirred at 80 °C in a silicone oil bath for 16 h. The reaction mixture was then exposed to air and quenched with MeOH (5 mL). After concentration *in vacuo*, the residue was redissolved in CH_2Cl_2 (10 mL). The mixture was passed through a 5 cm SiO_2 pad and washed with CH_2Cl_2:MeOH:aq NH_4OH (90:9:1) (75 mL). Following concentration *in vacuo*, purification *via* automated flash column chromatography on SiO_2 gel (12 g) in 99:1 40–60 °C petroleum ether–Et_3N (5 CV) then 99:0:1→0:99:1 40–60 °C petroleum ether–EtOAc–Et_3N (over 15 CV) then 99:1 EtOAc–Et_3N (5 CV) and drying *in vacuo* at 60 °C for 48 h gave **20** as colourless, amorphous solid (240 mg, 56%).

Data for **20**:

^{1}H NMR: (400 MHz, $CDCl_3$)

8.28 (d, J = 1.7 Hz, 1H, C(7′)*H*), 7.58 (d, J = 1.7 Hz, 1H, C(5′)*H*), 5.35 (s, 1H, N*H*), 3.75–3.62 (m, 2H, C(2)H_A, C(6)H_A), 3.35–3.23 (m, 2H, C(2)H_B, C(6)H_B), 2.73 (t, J = 6.6 Hz, 2H, C(4′)H_2), 1.74 (t, J = 6.6 Hz, 2H, C(3′)H_2), 1.65–1.55 (m, 4H, C(3)H_2, C(5)H_2), 1.46 (s, 9H, OC(CH_3)$_3$), 1.32 (s, 12H, –OC(CH_3)$_2$–)

<u>^{13}C NMR:</u> (125 MHz $CDCl_3$)

156.9 (*C*=O), 154.9 (*C*(9′)), 153.6 (*C*(7′)), 142.4 (*C*(5′)), 114.4 (*C*(10′)), 83.6 (–O*C*(CH_3)$_2$–)), 79.9 (O*C*(CH_3)$_3$), 50.2 (*C*(4)), 39.5 (br s, *C*(2), *C*(6)), 37.0 (*C*(3), *C*(5)), 31.9 (*C*(3′)), 28.6 (OC(CH$_3$)$_3$), 24.9 (–OC(*C*H$_3$)$_2$–), 22.4 (*C*(4′))

<u>^{11}B NMR:</u> (160 MHz)

30 (br s)

<u>IR:</u> (neat)

3304 (w), 2973 (w), 2928 (w),1686 (m), 1604 (m), 1561 (w), 1509 (w), 1475 (w), 1423 (m), 1396 (m), 1384 (m), 1356 (m), 1308 (m), 1283 (m), 1248 (m), 1213 (w), 1155 (m), 1141 (s), 1111 (w), 1082 (m), 1032 (w), 997 (w), 979 (w), 966 (w), 926 (w), 859 (m), 830 (w), 772 (m), 697 (w), 678 (m), 659 (w), 608 (w), 599 (w), 579 (w), 558 (w)

<u>HRMS:</u> (ESI$^+$)

Calcd for $C_{23}H_{36}BN_3O_4$: 428.2835, found: 428.2842

I. Access to Other Halogenated THN Isomers 23 and 25

Preparation of *tert*-butyl 7′-chloro-3′,4′-dihydro-1′*H*-spiro[piperidine-4,2′-[1,8]naphthyridine]-1-carboxylate (23)

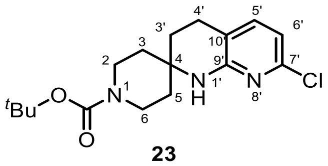

23

Following the **General Procedure** (section B), 5 mL of reagent feed A [2,6-dichloro-3-vinylpyridine **22** (261 mg, 1.50 mmol, 1.0 equiv) and 3DPA2FBN (9.6 mg, 15.0 μmol, 1 mol%) in anhydrous DMF], 5 mL of reagent feed B [*tert*-butyl 4-aminopiperidine-1-carboxylate **10i** (300 mg, 1.50 mmol, 1.0 equiv) and NaN_3 (19.5 mg, 300 μmol, 20 mol%) in anhydrous DMF], and 10 mL of reagent feed C [DIPEA (291 mg, 2.25 mmol, 1.5 equiv) in anhydrous DMF] were reacted in flow, setting the high-temperature tube reactor to 200 °C. The steady state mixture (10 mL) was collected and concentrated *in vacuo* on an Asynt spiral evaporator. Purification *via* automated flash column chromatography on SiO_2 gel (12 g) in 40–60 °C petroleum ether (5 CV) then 100:0→0:100 40–60 °C petroleum ether–EtOAc (over 20 CV) then EtOAc (5 CV) gave **23** as colourless, crystalline solid (172 mg, 68%, 1.62 mmol h^{-1}).

Data for **23**:

mp: 136–138 °C

^{1}H NMR: (400 MHz, $CDCl_3$)

7.12 (d, 1H, J = 7.2, C(5′)H), 6.51 (dd, J = 7.2, 1H, C(6′)H), 5.14 (s, 1H, NH), 3.75–3.67 (m, 2H, C(2)H_A, C(6)H_A), 3.27–3.22 (m, 2H, C(2)H_B, C(6)H_B), 2.69 (t, J = 6.4 Hz, 2H, C(4′)H_2), 1.73 (t, J = 6.4 Hz, 2H, C(3′)H_2), 1.64–1.56 (m, 4H, C(3)H_2, C(5)H_2), 1.46 (s, 9H, OC(CH_3)$_3$)

^{13}C NMR: (101 MHz $CDCl_3$)

154.9 (C=O), 154.8 (C(9′)), 147.3 (C(7′)), 138.8 (C(5′)), 113.4 (C(10′)), 112.0 (C(6′)), 79.9 (OC(CH_3)$_3$), 50.1 (C(4)), 39.5 (br s, C(2), C(6)), 36.9 (C(3), C(5)), 31.8 (C(3′)), 28.5 (OC(CH$_3$)$_3$), 22.0 (C(4′))

IR: (neat)

3403 (w), 3044 (w), 2992 (w), 2975 (w), 2929 (w), 2782 (w), 1682 (s), 1588 (m), 1570 (w), 1482 (s), 1463 (m), 1435 (s), 1422 (s), 1411 (m), 1389 (w), 1370 (m), 1278 (m), 1266 (m), 1253 (s), 1209 (w), 1167 (m), 1158 (s), 1121 (m), 1092 (m), 1079 (m), 1041 (w). 1022 (m), 992 (m), 981 (m), 955 (w), 947 (w), 921 (w), 912 (m), 887 (w), 825 (w), 766 (m), 758 (m), 722 (w), 674 (m), 581 (m), 561 (w), 552 (m)

HRMS: (ESI$^+$)

Calcd for $C_{17}H_{24}ClN_3O_2$: 337.1557, found: 337.1562.

Preparation of *tert*-butyl 5′-chloro-3′,4′-dihydro-1′*H*-spiro[piperidine-4,2′-[1,8]naphthyridine]-1-carboxylate (25)

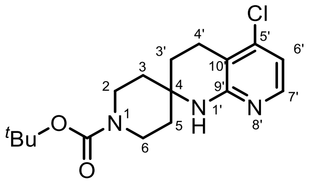

25

Following the **General Procedure** (section B), 5 mL of reagent feed A [4-chloro-2-fluoro-3-vinylpyridine **24** (236 mg, 1.50 mmol, 1.0 equiv) and 3DPA2FBN (9.6 mg, 15.0 μmol, 1 mol%) in anhydrous DMF], 5 mL of reagent feed B [*tert*-butyl 4-aminopiperidine-1-carboxylate **10i** (300 mg, 1.50 mmol, 1.0 equiv) and NaN_3 (19.5 mg, 300 μmol, 20 mol%) in anhydrous DMF], and 10 mL of reagent feed C [DIPEA (291 mg, 2.25 mmol, 1.5 equiv) in anhydrous DMF] were reacted in flow, setting the high-temperature tube reactor to 200 °C. The steady state mixture (10 mL) was collected and concentrated *in vacuo* on an Asynt spiral evaporator. Purification *via* automated flash column chromatography on SiO_2 gel (12 g) in 40–60 °C petroleum ether (5 CV) then 100:0→0:100 40–60 °C petroleum ether–EtOAc (over 20 CV) then EtOAc (5 CV) gave **25** as a pale yellow, amorphous solid (77 mg, 31%, 0.74 mmol h^{-1}).

Data for **25**:

^{1}H NMR: (400 MHz, $CDCl_3$)

7.77 (d, *J* = 5.4, 1H, C(7′)*H*), 6.59 (d, *J* = 5.4 Hz, 1H, C(6′)*H*), 5.18 (s, 1H, N*H*), 3.70–3.65 (m, 2H, C(2)H_A, C(6)H_A), 3.37–3.23 (m, 2H, C(2)H_B, C(6)H_B), 2.78 (t, *J* = 6.4 Hz, 2H, C(4′)H_2), 1.77 (t, *J* = 6.4 Hz, 2H, C(3′)H_2), 1.62–1.57 (m, 4H, C(3)H_2, C(5)H_2), 1.46 (s, 9H, OC(CH_3)$_3$)

^{13}C NMR: (101 MHz $CDCl_3$)

156.0 (*C*(9′)), 154.7 (*C*=O), 145.5 (*C*(7′)), 143.4 (*C*(5′)), 114.0 (*C*(10′)), 113.3 (*C*(6′)), 79.8 (O*C*(CH_3)$_3$), 49.9 (*C*(4)), 39.4 (br s, *C*(*2*), *C*(*6*)), 36.4 (*C*(3), *C*(5)), 31.3 (*C*(3′)), 28.5 (OC(*C*H_3)$_3$), 20.4 (*C*(4′))

IR: (neat)

3240 (w), 3096 (w), 2998 (w), 2935 (w),1688 (s), 1604 (m), 1583 (m), 1522 (m), 1470 (w), 1455 (m), 1426 (m), 1382 (w), 1366 (m), 1322 (w), 1290 (m), 1250 (m), 1232 (m),

1168 (m), 1123 (m), 1103 (m), 1074 (m), 1027 (w), 992 (m), 985 (w), 950 (w), 936 (w), 915 (w), 906 (w), 882 (w), 867 (w), 825 (w), 761 (s), 716 (w), 682 (w)

HRMS: (ESI$^+$)

Calcd for $C_{17}H_{24}ClN_3O_2$: 337.1557, found: 337.1563.

J. Stepwise Synthesis of 1,7- & 1,5-THNs 28 and 31

Preparation of *tert*-butyl-7′-methyl-3′,4′-dihydro-1′*H*-spiro[pyrrolidine-3,2′[1,8]naphthyridine]-1-carboxylate (28)

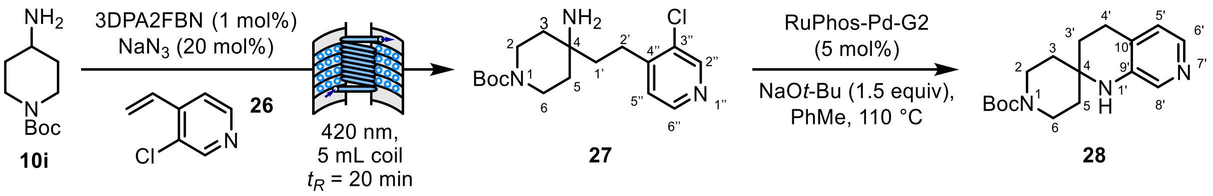

Following **General Procedure** (Section B), 5 mL of feed A [3-chloro-4-vinylpyridine **26** (231 mg, 1.50 mmol, 1.0 equiv), 3DPA2FBN (9.6 mg, 15.0 µmol, 1 mol%) in anhydrous DMF], and 5 mL of feed B [*tert*-butyl 4-aminopiperidine-1-carboxylate **10i** (300 mg, 1.50 mmol, 1.0 equiv) and NaN_3 (19.5 mg, 300 µmol, 20 mol%) in anhydrous DMF] were reacted in flow. The steady state mixture (5.0 mL) was collected and concentrated *in vacuo* on an Asynt spiral evaporator. Purification *via* automated flash column chromatography on SiO_2 gel (12 g) in 40–60 °C petroleum ether (5 CV) then 100:0:0:0→0:66:32:2 40–60 °C petroleum ether–EtOAc–EtOH–aq. NH_4OH (over 20 CV) then 0:66:32:2 40–60 °C petroleum ether–EtOAc–EtOH–aq. NH_4OH (5 CV), followed by automated flash column chromatography on C_{18} reversed-phase silica gel (50 g) in H_2O (3 CV) then 100:0→0:100 H_2O–MeOH (over 25 CV) then MeOH (10 CV) gave **27** as a brown oil (63 mg, 25%).

Data for **27**:

^{1}H NMR: (400 MHz, $CDCl_3$)

8.50 (s, 1H, C(2″)*H*), 8.36 (d, 1H, *J* = 5.0 Hz, C(6″)*H*), 7.15 (d, 1H, *J* = 5.0 Hz, C(5″)*H*), 3.74–3.60 (m, 2H, C(2)H_A, C(6)H_A), 3.34–3.25 (m, 2H, C(2)H_B, C(6)H_B), 2.82–2.73 (m, 2H, C(2′)*H2*), 1.67–1.60 (m, 2H C(1′)H_2), 1.58–1.37 (m, 13H, C(3)H_2, C(5)H_2, OC(CH_3)$_3$).

^{13}C NMR: (101 MHz $CDCl_3$)

155.0 (*C*=O), 149.6 (*C*(2″)), 149.0 (*C*(4″)), 148.0 (*C*(6″)), 132.1 (*C*(3″)), 124.9 (*C*(5″)), 79.6 (O*C*(CH$_3$)$_3$), 49.6 (*C*(4)), 42.6 (*C*(1′)), 40.1 (*C*(2), *C*(6)), 37.7 (*C*(3), *C*(5)), 28.6 (OC(*C*H$_3$)$_3$), 26.9 (*C*(2′)).

IR: (neat)

3362 (w), 2985 (w), 2930 (w),1680 (m), 1596 (w), 1552 (w), 1477 (w), 1456 (m), 1400 (s), 1366 (m), 1254 (w), 1220 (w), 1164 (m), 1120 (m), 1085 (m), 985 (w), 886 (m), 832 (w), 777 (m), 736 (w), 680 (w), 640 (w), 615 (w), 585 (w), 575 (w).

HRMS: (ESI$^+$)

Calcd for $C_{17}H_{26}ClN_3O_2$: 339.1714, found: 339.1720

In a nitrogen purge box, a 10-mL microwave vial equipped with a magnetic stirrer bar was charged with **27** (63 mg, 190 µmol, 1.0 equiv), NaO*t*-Bu (27 mg, 280 µmol, 1.5 equiv), RuPhos-Pd-G2 (7.2 mg, 9.3 µmol, 5 mol%), and anhydrous PhMe (1.9 mL). The vial was then capped with an aluminium crimp seal and removed from the purge box, then stirred at 110 °C in a silicone oil bath for 16 h. After allowing the mixture to cool to rt, it was diluted with EtOAc (10 mL), filtered through a 2-cm pad of Celite®, and concentrated *in vacuo*. Purification *via* automated flash column chromatography on SiO_2 gel (12 g) in 40–60 °C petroleum ether (5 CV) then 100:0→0:100 40–60 °C petroleum ether–EtOAc (over 20 CV) then EtOAc (5 CV) gave *tert*-butyl **28** as colourless, crystalline solid (47 mg, 84%).

Data for **28**:

mp: 130–132 °C

^{1}H NMR: (400 MHz, $CDCl_3$)

7.88 (s, 1H, C(8′)*H*), 7.81 (d, *J* = 5.0 Hz, 1H, C(6′)*H*), 6.85 (d, *J* = 5.0 Hz, 1H, C(5′)*H*), 4.10 (s, 1H, N*H*), 3.66–3.54 (m, 2H, C(2)H_A, C(6)H_A), 3.38–3.29 (m, 2H, C(2)H_A, C(6)H_A), 2.72 (t, *J* = 6.8 Hz, 2H, C(4′)H_2), 1.75 (t, *J* = 6.8 Hz, 2H, C(4′)H_2), 1.64–1.52 (m, 4H, C(3)H_2, C(5)H_2), 1.45 (s, 9H, OC(CH_3)$_3$)

^{13}C NMR: (101 MHz $CDCl_3$)

154.9 (*C*=O), 140.2 (*C*(10′)), 138.4 (*C*(6′)), 136.8 (*C*(8′)), 128.2 (*C*(9′)), 123.7 (*C*(5′)), 79.9 (O*C*(CH_3)$_3$), 49.1 (*C*(4)), 39.7 (br s, *C*(2), *C*(6)), 36.4 (*C*(3), *C*(5)), 31.1 (*C*(3′)), 28.4 (OC(*C*H_3)$_3$), 22.6 (*C*(4′))

IR: (neat)

3232 (w), 2932 (w), 1670 (m), 1603 (m), 1510 (m), 1477 (w), 1422 (m), 1364 (m), 1340 (w), 1302 (w), 1277 (m), 1263 (m), 1249 (m), 1213 (w), 1152 (s), 1079 (m), 1054 (m), 1025 (m), 998 (w), 981 (w), 959 (w), 908 (w), 873 (m), 822 (m), 748 (m), 728 (m), 694 (w), 643 (m), 609 (w), 601 (w), 585 (w), 578 (m), 562 (m)

HRMS: (ESI+)

Calcd for $C_{17}H_{25}N_3O_2$: 303.1947, found: 303.1950

Preparation of *tert*-butyl 3′,4′-dihydro-1′*H*-spiro[piperidine-4,2′[1,5]naphthyridine]-1-carboxylate (31)

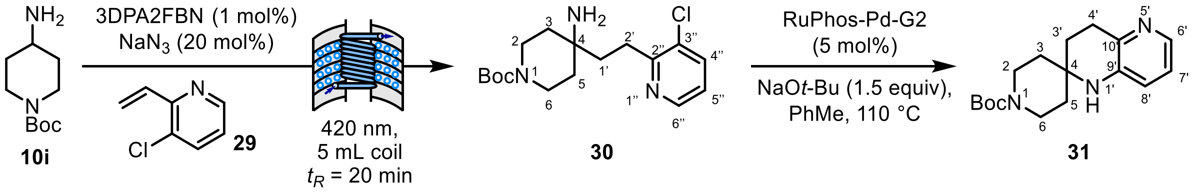

Following **General Procedure** (Section B), 5 mL of feed A [3-chloro-2-vinylpyridine (**29**) (209 mg, 1.50 mmol, 1.0 equiv), 3DPA2FBN (9.6 mg, 15.0 µmol, 1 mol%) in anhydrous DMF], and 5 mL of feed B [*tert*-butyl 4-aminopiperidine-1-carboxylate **10i** (300 mg, 1.50 mmol, 1.0 equiv) and NaN_3 (19.5 mg, 300 µmol, 20 mol%) in anhydrous DMF] were reacted in flow. The steady state mixture (5.0 mL) was collected and concentrated *in vacuo* on an Asynt spiral evaporator. Purification *via* automated flash column chromatography on SiO_2 gel (12 g) in 40–60 °C petroleum ether (5 CV) then 100:0:0:0→0:66:32:2 40–60 °C petroleum ether–EtOAc–EtOH–aq. NH_4OH (over 20 CV) then 0:66:32:2 40–60 °C petroleum ether–EtOAc–EtOH–aq. NH_4OH (5 CV), followed by automated flash column chromatography on C_{18} reversed-phase silica gel (50 g) in H_2O (3 CV) then 100:0→0:100 H_2O–MeOH (over 25 CV) then MeOH (10 CV) gave **30** as a yellow oil (87 mg, 50%).

Data for **30**:

^{1}H NMR: (400 MHz, $CDCl_3$)

8.40 (d, *J* = 4.6 Hz, 1H, C(6″)*H*), 7.62 (d, *J* = 8.0 Hz, 1H, C(4″)*H*), 7.09 (dd, *J* = 8.0, 4.6 Hz, 1H, C(5″)*H*), 3.75–3.55 (m, 2H, C(2)H_A, C(6)H_A), 3.29 (ddd, *J* = 13.6, 10.1, 3.3 Hz, 2H, C(2)H_B, C(6)H_B), 3.03–2.93 (m, 2H, C(2′)H_2), 1.84–1.72 (m, 2H, C(1′)H_2), 1.62–1.51 (m, 2H,), 1.46–1.38 (m, 11H, C(3)H_2, C(5)H_2, OC(CH_3)$_3$).

^{13}C NMR: (101 MHz $CDCl_3$)

159.4 (*C*(2″)), 155.0 (*C*=O), 147.4 (*C*(6″)), 137.1 (*C*(4″)), 131.2 (*C*(3″)), 122.4 (*C*(5″)), 79.5 (O*C*(CH_3)$_3$), 49.6 (*C*(4)), 41.6 (*C*(1′)), 40.0 (br, *C*(2), *C*(6)), 37.7 (*C*(3), *C*(5)), 29.5 (*C*(2′)), 28.6 (OC(*C*H_3)$_3$).

IR: (neat)

2934 (w), 2349 (w), 2301 (w), 2011 (w), 1688 (s), 1425 (s), 1365 (m), 1247 (m), 1167 (m), 769 (w)

HRMS: (ESI^+)

Calcd $[M+H]^+$ for $C_{17}H_{26}ClN_3O_2$: 340.1786, found: 340.1795

In a nitrogen purge box, a 10-mL microwave vial equipped with a magnetic stirrer bar was charged with **30** (71 mg, 0.21 mmol, 1.0 equiv), NaO*t*-Bu (30 mg, 0.32 mol, 1.5 equiv), RuPhos-Pd-G2 (8.1 mg, 11 µmol, 5 mol%), and anhydrous PhMe (2.1 mL). The vial was then capped with an aluminium crimp seal and removed from the purge box, then stirred at 110 °C in a silicone oil bath for 16 h. After allowing the mixture to cool to rt, it was diluted with EtOAc (10 mL), filtered through a 2-cm pad of Celite®, and concentrated *in vacuo*. Purification *via* automated flash column chromatography on SiO_2 gel (12 g) in 40–60 °C petroleum ether (5 CV) then 100:0→0:100 40–60 °C petroleum ether–EtOAc (over 20 CV) then EtOAc (10 CV) gave **31** as a pale yellow, crystalline solid (59 mg, 93%).

Data for **31**:

mp: 129–130 °C

^1H NMR: (400 MHz, $CDCl_3$)

7.89 (dd, *J* = 4.6, 1.5 Hz, 1H, C(6′)*H*), 6.91 (dd, *J* = 8.1, 4.6 Hz, 1H, C(7′)*H*), 6.76 (dd, 1H, *J* = 8.1, 1.5 Hz, C(8′)*H*) , 3.83 (s, 1H, N*H*), 3.64–3.53 (m, 2H, C(2)H_A, C(6)H_A), 3.43–3.32 (m, 2H, C(2)H_B, C(6)H_B), 2.93 (t, *J* = 6.8 Hz, 2H, C(4′)H_2), 1.87 (t, *J* = 6.8 Hz, 2H, C(4′)H_2), 1.69–1.53 (m, 4H, C(3)H_2, C(5)H_2), 1.46 (s, 9H, OC(CH_3)$_3$).

^{13}C NMR: (101 MHz $CDCl_3$)

155.0 (*C*=O), 142.1 (*C*(10′)), 139.2 (*C*(9′)), 138.5 (*C*(6′)), 122.1 (*C*(7′)), 120.9 (*C*(8′)), 79.9 (O*C*(CH_3)$_3$), 49.2 (*C*(4)), 39.8 (br s, *C*(2), *C*(6)), 36.5 (*C*(3), *C*(5)), 31.3 (*C*(3′)), 28.5 (OC(*C*H_3)$_3$), 26.5 (*C*(4′)).

IR: (neat)

2970 (w), 2933 (w),1668 (m), 1580 (m), 1456 (w), 1421 (w), 1395 (s), 1364 (m), 1284 (w), 1249 (w), 1170 (m), 1152 (m), 1110 (m), 1075 (m), 1033 (w), 879 (w), 796 (w), 769 (m), 730 (w), 668 (w).

HRMS: (ESI^+)

Calcd $[M+H]^+$ for $C_{17}H_{25}N_3O_2$: 304.2020, found: 304.2035

K. Synthesis of Core (35) of Melanocortin MC4 Receptor Antagonist 5

Preparation of *tert*-butyl-7′-methyl-3′,4′-dihydro-1′*H*-spiro[pyrrolidine-3,2′[1,8]naphthyridine]-1-carboxylate (35)

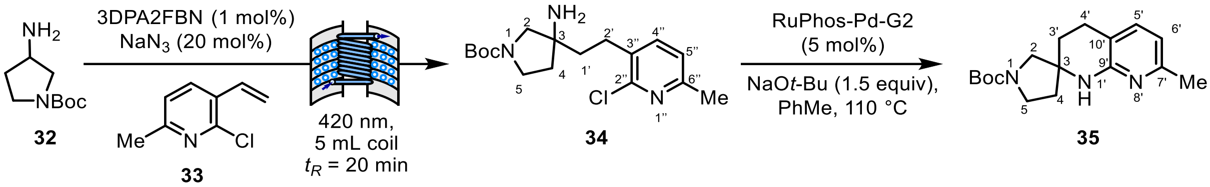

Following **General Procedure** (Section B), 10 mL of feed A [2-chloro-6-methyl-3-vinylpyridine (**33**) (461 mg, 3.0 mmol, 1.0 equiv), 3DPA2FBN (19.2 mg, 30.0 µmol, 1 mol%) in anhydrous DMF], and 5 mL of feed B [*tert*-butyl 3-aminopyrrolidine-1-carboxylate **32** (558 mg, 3.0 mmol, 1.0 equiv) and NaN_3 (39.1 mg, 600 µmol, 20 mol%) in anhydrous DMF] were reacted in flow. The steady state mixture (10.7 mL) was collected and concentrated *in vacuo* on an Asynt spiral evaporator. Purification *via* automated flash column chromatography on SiO_2 gel (12 g) in 40–60 °C petroleum ether (5 CV) then 100:0:0:0→0:66:32:2 40–60 °C petroleum ether–EtOAc–EtOH–aq. NH_4OH (over 20 CV) then 0:66:32:2 40–60 °C petroleum ether–EtOAc–EtOH–aq. NH_4OH (5 CV), followed by automated flash column chromatography on C_{18} reversed-phase silica gel (50 g) in H_2O (3 CV) then 100:0→0:100 H_2O–MeOH (over 25 CV) then MeOH (10 CV) gave **34** as a brown oil (427 mg, 79%).

Data for **34**:

^{1}H NMR: (400 MHz, $CDCl_3$)

7.37 (d, *J* = 7.6 Hz, 1H, C(4″)*H*), 6.96 (d, 1H, *J* = 7.6 Hz, C(5″)*H*), 3.50–3.36 (m, 2H, C(5)H_2), 3.50–3.36 (m, 2H, C(2)H_2), 2.79–2.63 (m, 2H, C(2′)H_2), 2.43 (s, 3H, CH_3), 1.89–1.66 (m, 4H, C(4)H_2, C(1′)H_2), 1.39 (s, 9H, OC(CH_3)$_3$)

^{13}C NMR: (101 MHz $CDCl_3$)

157.1 (*C*(6′)), 154.9 (*C*=O), 150.2 (*C*(2″)), 139.1 (*C*(4″)), 132.7 (*C*(3″)), 112.4 (*C*(5″)), 79.5 (O*C*(CH$_3$)$_3$), [59.0, 58.9 (*C*(3))], 58.4 (*C*(2)), [44.8, 44.3 (*C*(5))], [40.1, 39.9 (*C*(4)], [38.8, 38.3 (*C*(1′)], 28.7 (OC(*C*H$_3$)$_3$), 28.3 (*C*(2′)), 23.8 (*C*H$_3$)

Carbon signals in square brackets are assigned to two different rotamers

IR: (neat)

3366 (w), 2973 (w), 2929 (w),1682 (m), 1595 (w), 1551 (w), 1476 (w), 1454 (m), 1401 (s), 1364 (m), 1252 (w), 1225 (w), 1165 (m), 1121 (m), 1070 (m), 981 (w), 880 (m), 833 (w), 771 (m), 733 (w), 681 (w), 645 (w), 615 (w), 585 (w)

HRMS: (ESI^+)

Calcd for $C_{17}H_{26}ClN_3O_2$: 339.1714, found: 339.1718

In a nitrogen purge box, a 10-mL microwave vial equipped with a magnetic stirrer bar was charged with **34** (68 mg, 200 μmol, 1.0 equiv), NaO*t*-Bu (28.8 mg, 300 μmol, 1.5 equiv), RuPhos-Pd-G2 (7.8 mg, 10 μmol, 5 mol%), and anhydrous PhMe (2.0 mL). The vial was then capped with an aluminium crimp seal and removed from the purge box, then stirred at 110 °C in a silicone oil bath for 16 h. After allowing the mixture to cool to rt, it was diluted with EtOAc (10 mL), filtered through a 2-cm pad of Celite®, and concentrated *in vacuo*. Purification *via* automated flash column chromatography on SiO_2 gel (12 g) in 40–60 °C petroleum ether (5 CV) then 100:0→0:100 40–60 °C petroleum ether 100:0→0:100 40–60 °C petroleum ether–EtOAc (over 20 CV) then EtOAc (5 CV) to give **35** as a colourless, crystalline solid (50.9 mg, 84%).

Data for **35**:

mp: 110–112 °C

^{1}H NMR: (400 MHz, $CDCl_3$)

7.12 (d, *J* = 7.2 Hz, 1H, C(5′)*H*), 6.42 (d, 1H, *J* = 7.2 Hz, C(6′)*H*) , 4.95 (s, 1H, N*H*), 3.59–3.24 (m, 4H, C(2)H_2, C(5)H_2), 2.79–2.63 (m, 2H, C(4′)H_2), 2.32 (s, 3H, CH_3), 2.01–1.75 (m, 4H, C(3′)H_2, C(4)H_2), [1.46, 1.44, s, 9H, OC(CH_3)$_3$)]

^{13}C NMR: (101 MHz $CDCl_3$)

[155.0, 154.8 (*C*=O)], 154.6 (*C*(9′)), 154.3 (*C*(7′)), 137.0 (*C*(5′)), 112.7 (*C*(6′)), [118.0, 111.7 (*C*(10′))], 79.7 (O*C*(CH_3)$_3$), [59.6, 58.8 (*C*(3))], [57.7, 57.1 (*C*(2)], [44.2, 43.8 (*C*(5)], [38.1, 37.4 (*C*(4))], 29.7 (*C*(3′)), 28.6 (OC(*C*H_3)$_3$), 23.9 (*C*H_3), 23.8 (*C*(4′))

Carbon signals in square brackets are assigned to two different rotamers

IR: (neat)

3323 (w), 2972 (w), 2926 (w),1690 (m), 1600 (m), 1585 (m), 1465 (w), 1396 (s), 1364 (m), 1284 (w), 1243 (w), 1169 (m), 1152 (m), 1112 (m), 1100 (m), 1075 (m), 1032 (w), 981 (m), 907 (w), 879 (w), 796 (w), 769 (m), 730 (w), 681 (w), 618 (w), 593 (w), 576 (w), 554 (w)

HRMS: (ESI^+)

Calcd for $C_{17}H_{25}N_3O_2$: 303.1947, found: 303.1951

Supplementary References

1. Askey, H. E. et al. Photocatalytic hydroaminoalkylation of styrenes with unprotected primary alkylamines. *J. Am. Chem. Soc.* **143**, 15936–15945 (2021).

2. Jobelius, H. H. & Scharff, H.-D. Hydrazoic Acid and Azides. Ullmann's Encyclopedia of Industrial Chemistry (Wiley-VCH, 2000).

3. Plutschack, M. B., Pieber, B., Gilmore, K. & Seeberger, P. H. The hitchhiker’s guide to flow chemistry. *Chem. Rev.* **117**, 11796–11893 (2017).

4. Chen, S., Yang, L., Shang, Y., Mao, J. & Walsh, P. J. Base-promoted tandem synthesis of 2-azaaryl tetrahydroquinolines. *Org. Lett.* **23**, 1594–1599 (2021).

5. Li, M. et al. Unimolecular anion-binding catalysts for selective ring-opening polymerization of *o*-carboxyanhydrides. *Angew. Chem. Int. Ed.* **60**, 6003–6012 (2021).

6. Vermeulen, N. A. et al. Aromatizing olefin metathesis by ligand isolation inside a metal–organic framework. *J. Am. Chem. Soc.* **135**, 14916–14919 (2013).

7. Kong, M. et al. Catalytic reductive cross coupling and enantioselective protonation of olefins to construct remote stereocenters for azaarenes. *J. Am. Chem. Soc.* **143**, 4024–4031 (2021).

8. Tsoung, J., Panteleev, J., Tesch, M. & Lautens, M. Multicomponent-multicatalyst reactions $(MC)^2R$: Efficient dibenzazepine synthesis. *Org. Lett.* **16**, 110–113 (2014).

9. Xiong, B. et al. Ruthenium-catalyzed straightforward synthesis of 1,2,3,4-tetrahydronaphthyridines via selective transfer hydrogenation of pyridyl ring with alcohols. *Org. Lett.* **17**, 4054–4057 (2015).
