# Supplementary material for: Modular, automated synthesis of spirocyclic tetrahydronaphthyridines from primary alkylamines
Source: Commun Chem. 2023 Oct 4;6:215. doi: 10.1038/s42004-023-01012-2 (PMC10550966; doi:10.1038/s42004-023-01012-2)
Supplement: Supplementary file 2 — Description of Additional Supplementary Files [file 42004_2023_1012_MOESM2_ESM.pdf]

Description of Additional Supplementary Files

File name: Supplementary Data 1

Description: NMR Spectra
