# Supplementary material for: Modular, automated synthesis of spirocyclic tetrahydronaphthyridines from primary alkylamines
Source: Commun Chem. 2023 Oct 4;6:215. doi: 10.1038/s42004-023-01012-2 (PMC10550966; doi:10.1038/s42004-023-01012-2)
Supplement: Supplementary file 3 — Supplementary Data 1 [file 42004_2023_1012_MOESM3_ESM.docx]

**^1^H and {^1^H}^13^C NMR Spectra**

**^1^H NMR (400 MHz, CDCl_3_)**

**{^1^H}****^13^C NMR (101 MHz, CDCl_3_)**

**^1^H NMR (400 MHz, CDCl_3_)**

**{^1^H}****^13^CNMR (101 MHz, CDCl_3_)**

**^1^H NMR (400 MHz, CDCl_3_)**

**{^1^H}^13^C NMR (101 MHz, CDCl_3_)**

**^1^H NMR (400 MHz, CDCl_3_)**

**{^1^H}****^13^C NMR (101 MHz, CDCl_3_)**

**^1^H NMR (400 MHz, CDCl_3_)**

**{^1^H}^13^C NMR (101 MHz, CDCl_3_)**

**^1^H NMR (400 MHz, CDCl_3_)**

**{^1^H}^13^C NMR (101 MHz, CDCl_3_)**


**^1^H NMR (400 MHz, CDCl_3_)**

**{^1^H}^13^C NMR (101 MHz, CDCl_3_)**


**^1^H NMR (400 MHz, CDCl_3_)**

**{^1^H}****^13^C NMR (101 MHz, CDCl_3_)**

**^1^H NMR (400 MHz, CDCl_3_)**

**{^1^H}^13^C NMR (101 MHz, CDCl_3_)**

**^1^H NMR (400 MHz, CDCl_3_)**

**{^1^H}^13^C NMR (101 MHz, CDCl_3_)**

**^1^H NMR (400 MHz, CDCl_3_)**

**{^1^H}****^13^C NMR (101 MHz, CDCl_3_)**

**^1^H NMR (400 MHz, CDCl_3_)**

**{^1^H}^13^C NMR (101 MHz, CDCl_3_)**

**^1^H NMR (400 MHz, CDCl_3_)**

**{^1^H}^13^C NMR (101 MHz, CDCl_3_)**

**^1^H NMR (400 MHz, CDCl_3_)**

**{^1^H}^13^C NMR (101 MHz, CDCl_3_)**


**^1^H NMR (400 MHz, CDCl_3_)**

**{^1^H}^13^C NMR (101 MHz, CDCl_3_)**

**^1^H NMR (400 MHz, CDCl_3_)**

**{^1^H}^13^C NMR (101 MHz, CDCl_3_)**

**^1^H NMR (400 MHz, CDCl_3_)**

**{^1^H}^13^C NMR (101 MHz, CDCl_3_)**

**^1^H NMR (400 MHz, CDCl_3_)**

**{^1^H}****^13^C NMR (101 MHz, CDCl_3_)**

**^1^H NMR (400 MHz, CDCl_3_)**

**{^1^H}^13^C NMR (101 MHz, CDCl_3_)**

**^1^H NMR (400 MHz, CDCl_3_)**

**{^1^H}^13^C NMR (101 MHz, CDCl_3_)**

**^1^H NMR (400 MHz, CDCl_3_)**

**{^1^H}^13^C NMR (101 MHz, CDCl_3_)**


**^1^H NMR (400 MHz, CDCl_3_)**

**{^1^H}****^13^C NMR (101 MHz, CDCl_3_)**

**^1^H NMR (400 MHz, CDCl_3_)**

**{^1^H}^13^C NMR (101 MHz, CDCl_3_)**

**^1^H NMR (400 MHz, CDCl_3_)**

**{^1^H}^13^C NMR (101 MHz, CDCl_3_)**

**^1^H NMR (400 MHz, CDCl_3_)**

**{^1^H}^13^C NMR (125 MHz, CDCl_3_)**

**^1^H NMR (400 MHz, CDCl_3_)**

**{^1^H}^13^C NMR (101 MHz, CDCl_3_)**

**^1^H NMR (400 MHz, CDCl_3_)**

**{^1^H}^13^C NMR (101 MHz, CDCl_3_)**

**^1^H NMR (400 MHz, CDCl_3_)**

**^13^C{^1^H} NMR (101 MHz, CDCl_3_)**

**^1^H NMR (400 MHz, CDCl_3_)**

**{^1^H}^13^C NMR (101 MHz, CDCl_3_)**

**^1^H NMR (400 MHz, CDCl_3_)**

**{^1^H}^13^C NMR (101 MHz, CDCl_3_)**

**^1^H NMR (400 MHz, CDCl_3_)**

**{^1^H}^13^C NMR (126 MHz, CDCl_3_)**

**^1^H NMR (400 MHz, CDCl_3_)**

**{^1^H}^13^C NMR (126 MHz, CDCl_3_)**

**^1^H NMR (400 MHz, CDCl_3_)**

**{^1^H}^13^C NMR (101 MHz, CDCl_3_)**
